# Impact of Plant-Based Drinks on Cardiometabolic Outcomes: A Systematic Review and Network Meta-analysis

**DOI:** 10.1016/j.advnut.2026.100595

**Published:** 2026-02-04

**Authors:** Sabina Wallerer, Julia Stadelmaier, Maria Petropoulou, Eva Kiesswetter, Jaqueline Beck, Elida Sina, Toni Meier, Kathrin Sedlmaier, Martin Kussmann, Hans Hauner, Lukas Schwingshackl

**Affiliations:** 1 Institute for Evidence in Medicine, Medical Center—University of Freiburg/Medical Faculty—University of Freiburg, Freiburg, Germany; 2 Institute of Medical Biometry and Statistics, Medical Center—University of Freiburg/Medical Faculty—University of Freiburg, Freiburg, Germany; 3 Institute for Sustainable Agriculture and Food Economics (INL e.V.) Halle (Saale), Germany; 4 Competence Center for Nutrition (KErn), Bavarian Research Organisation for Agriculture (LfL), Freising, Germany; 5 School of Life Science, Technical University of Munich, Munich, Germany; 6 Institute of Nutritional Medicine, Else Kröner Fresenius Center for Nutritional Medicine, School of Medicine and Health, Technical University of Munich, Munich, Germany

**Keywords:** plant-based drinks, milk, anthropometry, cardiometabolic risk, network meta-analysis, soy milk

## Abstract

The global demand for plant-based milk alternatives is rising, but their health effects compared with cow milk remain uncertain. Therefore, we conducted a systematic review and network meta-analysis (NMA) to compare different plant-based drinks with each other and with cow’s milk on cardiometabolic outcomes. A systematic search was conducted in 3 electronic databases (MEDLINE, Cochrane CENTRAL, and Web of Science) and 2 trial registries. Randomized controlled trials (RCTs) with a minimum duration of 3 wk comparing different plant-based drinks (e.g., soy, rice, and oat) with another or with cow milk were included. We rated risk of bias using RoB 2.0 tool. Anthropometric outcomes, blood lipids, fasting glucose, and blood pressure were pooled using mean differences (MDs). NMAs were performed using a random-effects model. The certainty of evidence was assessed using the Grading of Recommendations Assessment, Development and Evaluation approach. Fourteen RCTs involving 543 participants were included, comparing soy-, rice-, oat drinks, and cow milk. Overall, few differences were observed comparing plant-based drinks with another or with cow milk on cardiometabolic outcomes. Replacing 500 mL/d of cow’s milk with soy drink may reduce low-density lipoprotein (LDL) cholesterol [MD: –0.47 mmol/L (–0.85; –0.10); low certainty], but showed no effect for high-density lipoprotein (HDL) cholesterol [MD: 0.01 mmol/L (–0.03; 0.05); moderate certainty]. Oat drink may slightly reduce total cholesterol compared with cow milk and rice drink [MD: –0.12 mmol/L (–0.24; –0.01); MD: –0.23 mmol/L (–0.40; –0.05)], but the evidence is very uncertain. Replacing 500 mL/d of cow milk with soy drink may reduce systolic and diastolic blood pressure [MD: –8.23 mmHg (–10.90; –5.55); MD: –7.82 mmHg (–13.61; –2.02); low certainty]. Our findings suggest that evidence for cardiometabolic differences between plant-based drinks and cow milk is limited. However, soy drink may lower blood pressure and LDL cholesterol compared with cow milk. The certainty of evidence was mainly low, highlighting the need for high-quality RCTs.

This study was registered at PROSPERO as CRD42025638028.

**Statement of significance**

This network meta-analysis investigated the effects of different plant-based drinks on anthropometric outcomes, blood lipids, and glycemic control in adults. The findings suggest that soy and oat drinks may provide cardiometabolic benefits compared with cow milk. Substituting soy drink for cow milk may also reduce blood pressure.

## Introduction

Cow milk is a traditional and important component of Western diets and remains a major source of protein, calcium, vitamin B12, and riboflavin [1]. Dietary guidelines for adults in many countries recommend 2–3 servings of cow milk or dairy products/d [2]. In several high-income regions, cow milk consumption has plateaued or declined, whereas the demand for plant-based milk alternatives has risen sharply [3]. Soy, oat, rice, and almond drinks are now widely available, with global sales showing annual growth rates exceeding 10% [3]. Consumers often choose these products for perceived health benefits, environmental sustainability, or ethical reasons [4].

The nutritional profiles of plant-based drinks vary substantially. Soy drinks generally provide a similar protein content to cow milk, whereas oat and rice drinks are typically lower in protein and differ in carbohydrate quality [5]. Fortification, added sugars, and processing levels further increase variability [6]. These compositional differences raise questions about their effects on cardiometabolic outcomes, including adiposity, lipid metabolism, glycemic control, and blood pressure.

Observational studies indicate that plant-based diets may be linked to reduced risk of obesity, type 2 diabetes, and cardiovascular disease (CVD) [7]. Yet, specific evidence on plant-based drinks is limited. A few randomized controlled trials (RCTs) suggest that substituting cow milk with soy drinks can lower cholesterol concentrations [8,9], but findings are inconsistent and evidence for other drinks such as oat or rice is scarce [10,11]. Existing systematic reviews of RCTs have primarily examined pairwise comparisons of soy drinks with cow milk, but no review has simultaneously compared different plant-based drinks [12,13]. Traditional pairwise meta-analyses are restricted to direct comparisons, limiting their ability to evaluate multiple interventions simultaneously [14]. In contrast, network meta-analysis (NMA) allows integration of both direct and indirect evidence, enabling comparisons across several plant-based drinks and ranking their relative effects [14].

To date, no NMA has compared the effects of different plant-based drinks with each other and with cow milk on cardiometabolic markers. Therefore, the present study aimed to systematically identify and synthesize RCT evidence on soy, oat, rice, and other plant-based drinks compared with cow milk or with each other, focusing on anthropometric outcomes, blood lipids, glycemic control, and blood pressure.

## Methods

We report this systematic review with NMA according to the PRISMA Extension for NMA (PRISMA-NMA) checklist [15], and the PRISMA Statement for Reporting Literature Searches in Systematic Reviews (PRISMA-S) [16]. The protocol of this study was predefined and registered in the International Prospective Register of Systematic Reviews (PROSPERO; registration number CRD42025638028).

### Systematic literature search

We conducted a comprehensive literature search in 3 electronic databases [MEDLINE (via OVID), Cochrane Central Register of Controlled Trials (via CRSO), and Web of Science (via Clarivate), search date: 6 January, 2025] and 2 trial registries (clinicaltrials.gov and WHO International Clinical Trials Registry Platform, search date: 10 January, 2025) from inception to January 2025. The search strategy combined search blocks on “dairy alternatives” and “study design” (i.e., RCTs) and was reviewed by an information search specialist (JS). No language filter was applied. The detailed search strategies can be found in Supplemental Appendix 1. In addition, we conducted backward citation tracking on systematic reviews, identified by our searches, and screened the reference lists of all included studies.

### Eligibility criteria

We included studies in this systematic review fulfilling the following eligibility criteria:

#### Population

We considered studies conducted in the general population (age ≥4 y). Studies focusing on pregnant females, malnourished patients, and patients with chronic diseases (e.g., nephropathy, rheumatism, cancer, CVD, and type 2 diabetes) were excluded, as well as studies focusing on populations with intervention-related allergies (e.g., casein and whey).

#### Intervention

Studies evaluating the intake of any plant-based drinks (e.g., soy-, oat-, or rice drink) were eligible to be included. Animal-based dairy alternatives (e.g., from sheep and goats), milk/protein isolates (e.g., whey or casein), formula, capsules, phytoestrogen fortified milk alternatives, and fermented milk products with additional microbiota strains added (beyond those naturally occurring) were not eligible.

#### Comparator

Any other plant-based drink or cow milk was considered as comparator. Cointerventions (e.g., physical activity and calorie restriction) were allowed as long as they were balanced across study arms within an RCT.

#### Outcomes

Any of the following health-related outcomes were considered eligible: anthropometric measures (body weight, fat mass, waist circumference, BMI, weight-for-age, and weight-for-height), cardiometabolic risk factors [blood pressure, blood glucose, glycosylated hemoglobin (HbA1c), total cholesterol, triglycerides, LDL cholesterol, and HDL cholesterol), bone health (bone mineral density (BMD)], all-cause mortality, CVD, type 2 diabetes, and cancer.

#### Study design

We included RCTs with a parallel or a cross-over design with a minimum duration of 3 wk.

Detailed eligibility criteria are displayed in Supplemental Table 1.

### Study selection

The search results were deduplicated using Systematic Review Accelerator (Bond University [17]) and Endnote 21 (Clarivate). Two reviewers from a group of 4 (EK, ES, JB, and SW) independently screened each title/abstract and full text of potentially eligible studies. All reasons for exclusion on the full text level were recorded. Any disagreements were resolved by discussion or with the help of a third reviewer (LS) if no agreement could be reached. The screening process was implemented using Covidence (Veritas Health Innovation) and was piloted with a set of 50 records.

### Data extraction

Two reviewers (JB and SW) independently extracted the data in a piloted data extraction sheet (Microsoft Excel). Conflicts were solved by discussion or with the help of a third reviewer (EK, JS, and LS) if no agreement could be reached. We extracted data on study characteristics [i.e., first author, publication year, study location (country), study design (parallel or cross-over), duration (study, intervention, wash-out period, and follow-up) and sample size (n randomly assigned)], participants’ characteristics [i.e., percentage of females, mean age, mean BMI, and health status (e.g., hypercholesterolemia, overweight or obesity)], intervention and comparator characteristics (i.e., type of plant-based drink or cow milk, dose, assessment and degree of adherence, any cointerventions), study funding, conflicts of interest, and outcomes. For all outcomes, we extracted (analysis of covariance-adjusted) mean postintervention or change scores as well as SD. If both postvalues and change scores were available, we preferred postvalues for the analysis [18,19]. In case a study did not report the SD, we calculated values from the corresponding SE [20,21]. If a study reported neither SD nor SE, we imputed the values by using the mean SD/SE of RCTs with similar Population, Intervention, Comparator, Outcome characteristics. If the relevant data were only displayed in figures, we used the "Web Plot Digitizer" [22] for extraction. For the analyses, data on blood lipids and fasting blood glucose given in mg/dL were converted to mmol/L [23,24]. If change scores were reported as percentage, we calculated absolute values from available data if possible. In case of insufficient or missing information, we made 2 attempts to contact the corresponding study authors by e-mail.

### Risk of bias assessment

Two reviewers out of a group of 3 (EK, JS, and SW) assessed the risk of bias (RoB) of each included study independently and any disagreements were resolved by consensus or with the help of a third reviewer (LS) if no agreement could be reached. We used the Cochrane Risk of Bias tool 2.0 (RoB 2) for parallel RCTs [25], and cross-over trials [26]. RoB 2 for parallel trials considers 5 domains: bias arising from the randomization process, bias due to deviations from the intended interventions, bias due to missing outcome data, bias in measurement of the outcome, and bias in selection of the reported results. For cross-over trials, an additional domain "bias arising from period and carryover effects" was assessed. We assessed the RoB for the effects of assignment to intervention and each domain as well as the overall RoB was judged as low RoB, some concerns or high RoB. Additional guidance for the RoB 2 assessment is provided in Supplemental Appendix 2 [27].

### Statistical analysis

The present systematic review represents a network of interventions. We performed pairwise and NMA to examine the intervention effects of substituting different plant-based drinks with another or cow milk as main analyses. All effect sizes, 95% confidence intervals (CIs), and SDs were standardized to a dose of 500 mL/d.

We initially performed pairwise meta-analyses to estimate all possible comparisons for each outcome of interest. If >2 interventions were available for an outcome and the network was connected, a frequentist NMA was performed [28,29]. Data were pooled using the generic inverse variance method with random-effects model and expressed as mean differences (MDs) with 95% CIs [19].

Results from the pairwise meta-analyses are presented in forest plots. Between-study variance (heterogeneity) was assessed using the $I^{2}$ statistic [30], and the magnitude of the heterogeneity ($\tau^{2}$) was estimated using the generalized DerSimonian and Laird estimator and the Q-profile approach [31,32].

Results from the NMA are presented using forest plots and league tables. The network structure for each outcome was illustrated using network plots. For each NMA, the transitivity assumption was assessed a priori. Transitivity implies that potential effect modifiers (study design, dietary setting, sex, and RoB) are balanced across available direct comparisons [33]. Additionally, we evaluated each network for global inconsistency using the random-effects design-by-treatment approach [34], and local inconsistency by splitting direct and indirect effects [35]. Interventions were ranked using *P*-scores, a frequentist version of the surface under the cumulative ranking curve [36]. *P*-scores are values between 0 and 1, where a value of 1 means that a treatment always ranks best and a value of 0 means that a treatment always ranks worst. When >10 studies were available for a given outcome, a comparison-adjusted funnel plot was generated to assess potential small study effects and publication bias [37,38].

Subgroup analyses were conducted by dietary setting (hypocaloric compared with eucaloric/ad libitum), sex, and study design (parallel compared with cross-over). If possible, we conducted sensitivity analyses excluding studies with a high RoB. Furthermore, we explored the robustness of effect estimates in sensitivity analyses using only postintervention values or only change scores. To gain additional insights into the associations between plant-based drinks and health-related outcomes, we performed nonlinear dose–response NMA. Several dose–response models were applied includingi.e., exponential, quadric polynomial, first-order fractional polynomials transformations with power p from the set {–2, –1, –0.5, 0, 0.5, 1, 2, 3}, and restricted cubic splines functions with knots placed at the 10%, 50% and 90% percentiles and at the 25%, 50% and 100% percentiles of the dose distribution. Model adequacy for each dose–response NMA was evaluated using heterogeneity measures and the Q/df ratio, reflecting the extent to which each model explains between-study variability without introducing excessive complexity [39]. Analyses were conducted using the R packages “metaphor” “netmeta,” and “netdose” [28,40,41].

### Certainty of evidence

We evaluated the certainty of the evidence according to the Grading of Recommendations Assessment, Development and Evaluation (GRADE) approach for NMA [42]. Two reviewers (JS, SW) independently rated the certainty of evidence in each of the direct, indirect, and network estimates for each outcome. Any disagreements were solved by discussion or with the help of a third reviewer (LS). Direct evidence was rated based on RoB, inconsistency, indirectness, and publication bias (if ≥10 studies were available). If the certainty of the direct evidence was *high* and its contribution was at least as much as that of the indirect evidence, we did not rate the indirect evidence [42]. If the rating of indirect evidence was needed, we used the certainty of direct estimates to inform indirect estimates considering the lowest of the ratings of the 2 direct comparisons forming the most dominant first-order loop. In the presence of serious intransitivity, we rated down the certainty of the indirect estimate. To address the certainty of network estimates, we compared the ratings for direct and indirect estimates. The estimate with the higher certainty was chosen and rated down if incoherence and/or imprecision were detected [42]. For the assessment of imprecision, we used outcome-specific thresholds (minimally important differences) [12,43,44]. If an NMA could not be conducted for a given outcome, we evaluated the certainty of the evidence from the pairwise meta-analysis based on RoB, inconsistency, indirectness, publication bias, and imprecision. All decisions to downgrade the certainty of evidence are given by informative footnotes. A more detailed guidance on rating of the certainty of the evidence can be found in Supplemental Appendix 3. Evidence profiles were created to summarize the evidence in a transparent and informative format [45].

## Results

**FIGURE 1:**
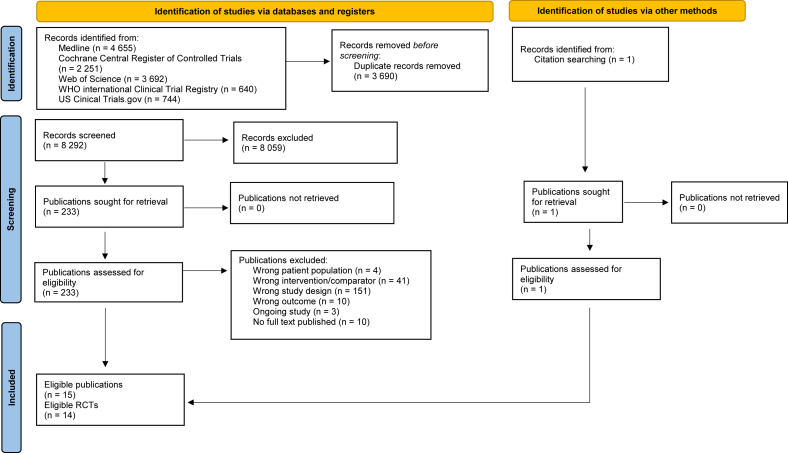
Flow chart of the process for study selection. RCT, randomized controlled trial.

The database searches resulted in 11,982 hits. After deduplication, we screened the eligibility of 8292 titles/abstracts and of 233 full texts. Finally, we included 14 RCTs (15 reports) [[8], [9], [10], [11],[46], [47], [48], [49], [50], [51], [52], [53], [54], [55], [56]]. The reasons for exclusion of full texts are given in Supplemental Table 2. The flow diagram of the search and screening process is depicted in Figure 1.

### Study and participant characteristics

Table 1 presents a summary of the main characteristics of included studies and participants; further information can be found in (Supplemental Table 3). Of the 14 included RCTs, 3 were conducted in Iran [46,48,50, 2[46,48,50], 2 each in the United States [8,11], Sweden [10,52], and Italy [54,55], and 1 each in Brazil [47], China [49], the United Kingdom [51], and Spain [53]. Eight RCTs had a cross-over design [8,46,47,50,52,[54], [55], [56], 5[8,46,47,50,52,54–56], 5 used a parallel design [11,48,49,51,53], and 1 used a mixed design (parallel with additional cross-over) [10]. The intervention duration ranged from 4 to 77 wk.

**TABLE 1:** Overview of included randomized controlled trials.

Study author (country, y)	Intervention	Study duration (wk)		Intervention duration (wk)	Sample size total	Female (%)	Age (mean, y)	BMI (mean, kg/m^2^)	Population
Parallel trials									

Beavers et al. (United States, 2010 [11])	Int. 1: soy drink Int. 2: cow milk	6.0		4.0	33	100	Soy: 53.9 Cow: 54.0	Soy: 25.4 Cow: 26.3	Generally healthy (postmenopausal)
Faghih et al. (Iran, 2011 [48])	Int. 1: soy drink Int. 2: cow milk	10.0		8.0	43	100	Soy: 37.5 Cow: 38.3	Soy: 31.1 Cow: 30.0	Generally healthy (premenopausal, overweight/obese)
Gui et al. (China, 2012 [49])	Int. 1: soy drink Int. 2: cow milk	77.4		77.4	100	100	Soy: 56.1 Cow: 55.8	Soy: 24.0 Cow: 24.9	Generally healthy (postmenopausal)
Mitchell et al. (United Kingdom, 1999 [51])	Int. 1: soy drink Int. 2: rice drink Int. 3: cow milk	6.0		4.0	10	0	Range: 20–50	—	Generally healthy
Önning et al.^1^ (Sweden, 1998 [10])	Int. 1: soy drink Int. 2: oat drink Int. 3: cow milk	11.0		4.0	24	50	31.5	—	Generally healthy
Rivas et al. (Spain, 2002 [53])	Int. 1: soy drink Int. 2: cow milk	16.9		12.9	40	37.5	Soy: 47.5 Cow: 49.4	—	Generally healthy (hypertension of degree 1 or 2)

Study author (country, y)	Intervention	Study duration (wk)	Wash-out period (wk)	Intervention duration (wk)	Sample size Total	Female (%)	Age (y)	BMI (kg/m^2^)	Population

Cross-over trials									

Azadbakht et al. (Iran, 2011 [46])	Int. 1: soy drink Int. 2: cow milk	18.0	3.0	6.0	33	100	22.1	28.1	Generally healthy (overweight/obese)
Bricarello et al. (Brazil, 2004 [47])	Int. 1: soy drink Int. 2: cow milk	18.0	0.0	6.0	60	75	56.0	24.9	Generally healthy (primary hypercholesterolemia, TC: 200–300 mg/dL)
Gardner et al. (United States, 2007 [8])	Int. 1: soy drink Int. 2: cow milk	24.0	2x4	4.0	31	79	52.0	26.0	Generally healthy (prestudy LDL cholesterol: 160–220 mg/dL)
Keshavarz et al. (Iran, 2012 [50])	Int. 1: soy drink Int. 2: cow milk	12.0	2.0	4.0	30	100	37.7	30.8	Generally healthy (premenopausal, overweight/obese)
Önning et al. (Sweden, 1999 [52])	Int. 1: oat drink Int. 2: rice drink	15.0	5.0	5.0	66	0	62.6	27.0	Generally healthy (moderate hypercholesterolemia)
Sirtori et al. (Italy, 2002 [55])	Int. 1: soy drink Int. 2: cow milk	20.5	4.0	4.0	20	80	59.5	24.2	Generally healthy (type II hypercholesterolemia)
Sirtori et al. (Italy, 1999 [54])	Int. 1: soy drink Int. 2: cow milk	24.6	4.0	4.0	21	62	51.9	24.4	Generally healthy (familial hypercholesterolemia)
Steele et al. (Australia, 1992 [56])	Int. 1: soy drink Int. 2: cow milk	8.0	0.0	4.0	37	53	42.2	—	Generally healthy

Abbreviations: Int, intervention arm; TC, total cholesterol.

1 Önning 1998 had a parallel design overall, but each study arm implemented 2 interventions in a cross-over design.

In total, the 14 RCTs included 543 participants. Sample size ranged from *n =* 10 [51], to *n =* 100 participants [49]. All studies included general adult (≥ 18 y) population with a mean age ranging from 20 to 64 y. Several studies focused on female participants only [9,11,46,[48], [49], [50]], whereas others enrolled mixed populations [8, 10, 47, [53], [54], [55], [56]]; 2 trials were conducted exclusively in males [51,52]. The mean BMI at baseline across studies ranged from 24 to 31 kg/m^2^. Information on funding or declarations of potential competing interests is detailed in Supplemental Table 3.

### Intervention characteristics

Most trials (*n =* 12) compared 2 intervention arms, whereas 2 RCTs [10,51] had a 3-arm design. The majority of RCTs investigated the substitution of cow milk with plant-based drinks, mostly soy (*n* = 13). Two trials compared oat drinks with cow milk [10] or rice drink [52]. One trial compared rice drink, with soy drink and cow’s milk.

In all but one study [48] cow milk and plant-based drinks were provided to the participants; for the remaining dietary intake, participants received dietary instructions [8,11,[46], [47], [48],^50^,[53], [54], [55], [56]]. Only 1 study provided all meals to participants [10], and 3 studies did not report any information on overall dietary intake [49,51,52]. In 4 studies, participants were instructed to restrict energy intake (200–500 kcal/d energy deficit) [11,46,48,50], whereas most studies had an eucaloric [54,55], or ad libitum [8,10,47,49,[51], [52], [53],^56^], dietary setting. Macronutrient compositions of the prescribed diets were similar: 50%–60% carbohydrates, 15%–20% protein, and <30% fat, low saturated fat and cholesterol intake. Dietary adherence was assessed via food records in most trials and indicated overall good compliance (>80% adherence) (Supplemental Table 3). Dietary fiber intake was comparable across study arms and trials, ranging from ∼15 to 20 g/d.

### Risk of bias

The results of the RoB assessment are provided in Supplemental Figures 1 and 2. A total of 67 RoB assessments were carried out with separate assessments for anthropometric outcomes (*n =* 13), blood lipids (*n =* 38), blood pressure (*n =* 8), glycemic outcomes (*n =* 5), and BMD (*n =* 3). Across all assessments, no RCT was judged to have an overall low RoB. For the majority of outcomes (76%), trials were rated as *high RoB*, whereas 24% were judged as *some concerns*. Reasons for a *high RoB* were inadequate randomization, analyses, or selective reporting. However, a *high RoB* was most frequently observed in cross-over trials, mainly due to inadequate wash-out periods (<4 wk). In contrast, the parallel trials were mostly judged as having *some concerns*.

### Anthropometric outcomes

**FIGURE 2:**
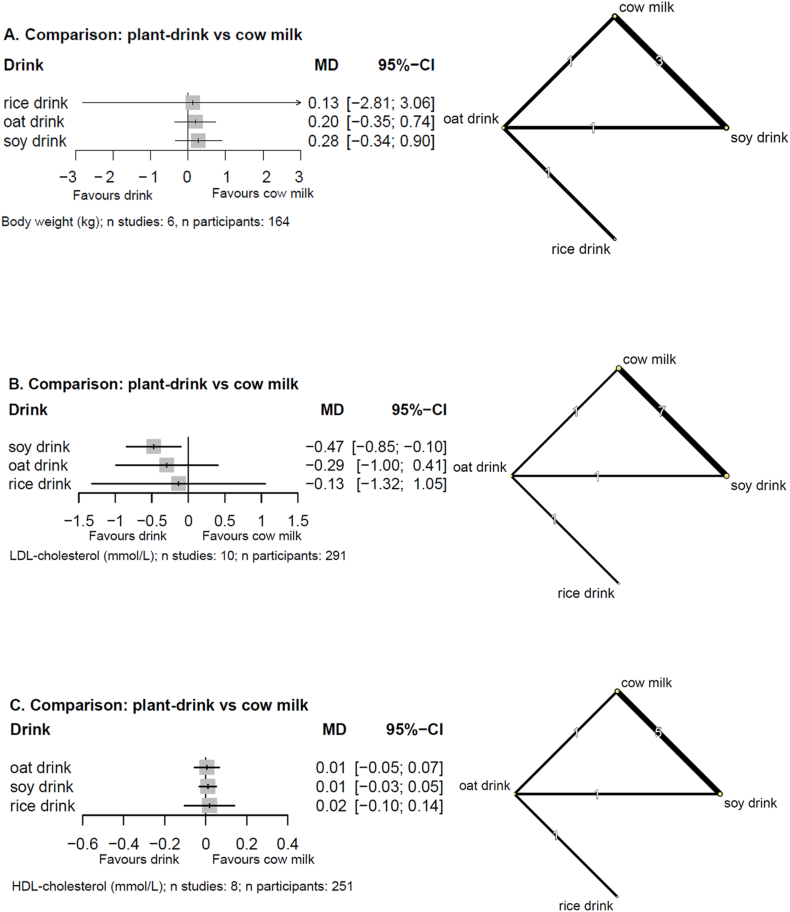

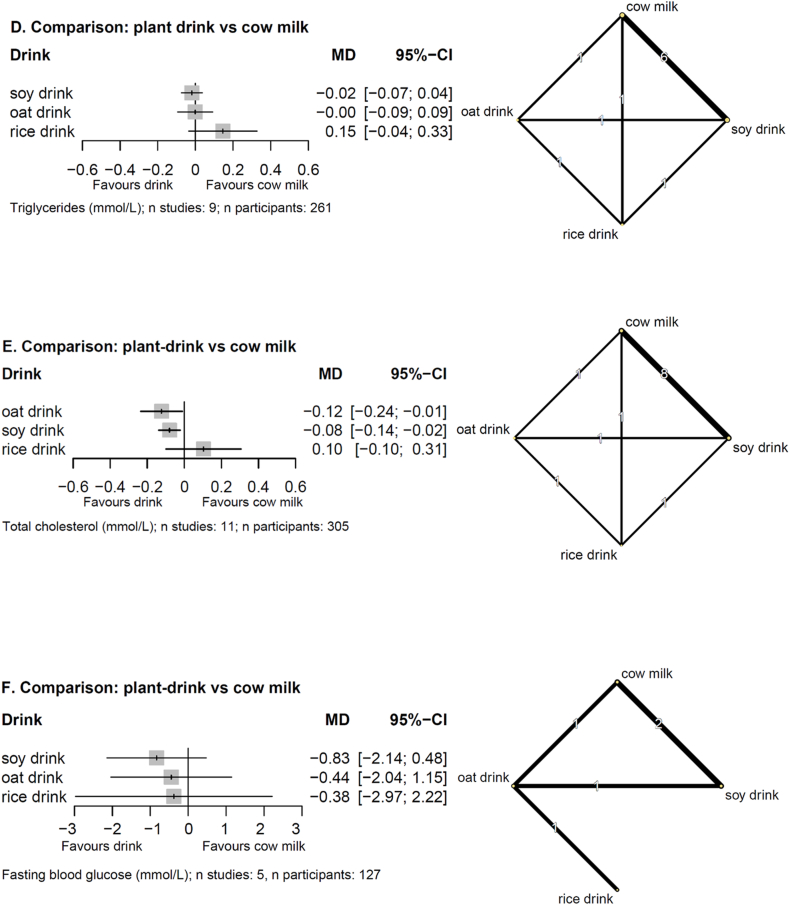
Forest plots of NMA estimates (MDs with 95% CIs) (left panels) and network plots (right panels) for (A) body weight, (B) LDL cholesterol, (C) HDL cholesterol, (D) triglycerides, (E) total cholesterol, (F) fasting blood glucose. Doses are harmonized per 500 mL/d. Network plots: line width corresponds to the number of studies; numbers: the number of studies directly comparing the treatments. 95% CI, 95% confidence interval; MD mean difference; NMA, network meta-analysis.

NMA results for body weight are presented in Figure 2A. Substituting any plant drink for another or any plant drink for cow milk may have little to no effect on body weight (low and very low certainty, Supplemental Figure 3 and Supplemental Tables 4 and 5).

For BMI, waist circumference, and fat mass, an NMA was not possible. Results from the pairwise meta-analyses suggest a slight reduction in BMI when substituting 500 mL of cow milk with soy drink [MD: –0.30 kg/m^2^ (95% CI: –0.57, –0.02 kg/m^2^), low certainty, Supplemental Table 6, Supplemental Figure 4]. Substituting 500 mL of cow milk with soy drink may result in little to no difference in waist circumference (low certainty, Supplemental Table 6, Supplemental Figure 5).

### Blood lipids

Results for blood lipids are reported in Figure 2B–E. Replacing 500 mL of cow milk with soy drink may reduce LDL cholesterol [MD: –0.47 mmol/L (95% CI: –0.85, –0.10 mmol/L), low certainty], whereas there is likely no effect on HDL cholesterol and triglycerides [MD: 0.01 mmol/L (95% CI: –0.03, 0.05 mmol/L), MD: –0.02 mmol/L (95% CI: –0.07, 0.04 mmol/L), moderate certainty, Supplemental Figures 6–8 and Supplemental Tables 7–12]. Substituting 500 mL of oat drink for either rice drink or cow milk may reduce total cholesterol, but the evidence is very uncertain [MD: –0.23 mmol/L (95% CI: –0.40, –0.05 mmol/L), MD: –0.12 mmol/L (95% CI: –0.24, –0.01 mmol/L), very low certainty, Supplemental Figure 9 and Supplemental Tables 13 and 14]. Results for all other comparisons on blood lipids were very imprecise and had low or very low certainty (Supplemental Tables 7–14).

### Glycemic control

The evidence is very uncertain about the effects on fasting glucose when replacing any plant-drink for each other or for cow milk. Results are presented in Figure 2F, Supplemental Figure 10, Supplemental Tables 15 and 16.

### Blood pressure

No NMA was conducted for systolic and diastolic blood pressure due to the network being disconnected. This implies that comparisons were available only between specific pairs of interventions (i.e., soy drink compared with cow milk), with no common comparator linking them to a third intervention (i.e., oat drink). The pairwise meta-analysis suggests replacing cow milk with soy drink results in a reduction of systolic and diastolic blood pressure [MD: –8.23 mmHg (95% CI: –10.9, –5.55 mmHg), MD: –7.82 mmHg (95% CI: –13.61, –2.02 mmHg), low certainty, Supplemental Table 6, Supplemental Figures 11 and 12].

### Bone mineral density

No meta-analysis or NMA could be conducted for BMD due to the inclusion of a single study [49]. This study reported cow milk as effective in preventing bone loss, whereas soy drink had no effect on BMD reduction.

### Other outcomes

No studies were found for any other eligible outcomes.

### *P*-score and rankings

P-scores are presented in Supplemental Table 17. We did not identify any intervention that ranked best across all outcomes.

### Inconsistency

Supplemental Table 18 provides the results of the design-by-treatment interaction model. There was statistically significant global inconsistency in the NMA for LDL cholesterol and fasting blood glucose outcomes (*P* value < 0.001). For both outcomes, local inconsistency was observed for the comparison between soy drink and cow milk (Supplemental Figures 6 and 10).

### Dissemination bias

Visual inspection of the comparison-adjusted funnel plot for LDL cholesterol and total cholesterol shows slight asymmetry (Supplemental Figures 13 and 14). However, the small number of studies limits the reliability of the visual assessment. Dissemination bias could not be assessed for other networks due to the limited number of studies.

### Additional analysis

#### Subgroup analyses

Subgroup analyses on body weight, blood lipids, and fasting glucose were conducted for study design, dietary setting (e.g., hypocaloric, eucaloric, and ad libitum), and sex, and confirmed the main findings (Supplemental Figures 15–20).

The effect estimates for most outcomes were robust in sensitivity analyses excluding RCTs with a high RoB. For total cholesterol, excluding studies with a high RoB revealed a stronger reduction when soy drinks replaced cow milk [MD: –0.36 mmol/L (95% CI: –0.56, –0.15 mmol/L) compared with MD: –0.08 mmol/L (95% CI: –0.14, –0.02 mmol/L)] (Supplemental Figures 21–25).

#### Sensitivity analyses

Sensitivity analyses using only postvalues or only change scores confirmed the main findings. In the sensitivity analysis of change scores, LDL cholesterol, fasting glucose, and diastolic blood pressure indicated smaller beneficial effects for soy drink compared with cow milk. This was likely driven by the harmonization of the intervention dose to 500 mL/d [Supplemental Tables 19–26, Supplemental Figures 26–35].

#### NMA with dose–response relationships

An overview of the selected dose–response models can be found in Supplemental Table 27 and the results of the analyses are provided in Supplemental Appendix 4 and Supplemental Figures 36–43. For most outcomes, these analyses confirmed the findings of the main NMA, while providing additional dose-dependent insights into the intervention effects. However, the dose–response curve for LDL cholesterol and fasting glucose indicated less pronounced effects with large CIs when comparing any plant-based drink with cow milk [Supplemental Figures 37A, B and 43A, B]. Of note, for systolic and diastolic blood pressure, the network was disconnected and no harmonized NMA could be conducted. Nevertheless, a dose–response NMA was possible as the dose–response NMA model can reconnect subnetworks when they contain common intervention agents [39]. The dose–response curve shows a consistent decline in systolic and diastolic blood pressure for all plant drinks compared with cow milk (Supplemental Figures 41–42).

## Discussion

To the best of our knowledge, this is the first NMA to evaluate the effects of different plant-based drinks such as soy, oat, and rice in relation to each other and to cow milk on cardiometabolic risk factors including anthropometric outcomes, blood lipids, glycemic control, and blood pressure. Fourteen RCTs with 543 participants were included. In summary, we observed no major differences between plant-based drinks when compared with each other. Compared with cow milk, soy drink consumption resulted in reductions in LDL cholesterol, whereas oat drink demonstrated favorable effects on total cholesterol compared with rice drink and cow milk. Rice drink did not affect any outcomes. No effects were observed for body weight and fasting glucose. Dose–response analyses confirmed most findings of the harmonized NMA, while providing additional dose-dependent insights into the intervention effects. Pairwise meta-analyses suggest a slight reduction in BMI when soy drink is compared with cow milk. Soy drink may also reduce blood pressure compared with cow milk. The certainty of evidence was mostly rated as low to very low, reflecting the limited number and size of included trials as well as methodological shortcomings.

### Comparison with other studies

#### Anthropometric outcomes

Our analyses found no effects of plant-based drinks on body weight, waist circumference, or fat mass. We did, however, observe a slight reduction in BMI when soy drink was compared with cow milk. A recent systematic review of soy drink compared with cow milk reported that the substitution had no effect on body weight, BMI, body fat, or waist circumference [12]. Similarly, a meta-analysis of 7 RCTs found no differences in body weight or BMI after soy drink consumption, but did observe a slight reduction in waist circumference [13].

Observational evidence on dairy products indicates likewise no association with overweight, obesity, or abdominal obesity [57]. To date, however, no observational studies have specifically addressed the association between plant-based drinks including soy drink and anthropometric outcomes.

#### Blood lipids

The majority of included trials in our NMA focused on blood lipid outcomes. Soy drink reduced LDL cholesterol by –0.47 mmol/L (–0.19 mmol/L in sensitivity analyses) compared with cow milk. Across the included trials, 5 involved participants with elevated LDL cholesterol concentrations (>3.0 mmol/L) [58], whereas 4 were within a low-risk range, yet the effect estimates were relatively consistent across studies. These results are in agreement with the findings of a recent systematic review, which also reported a modest reduction in LDL cholesterol (–0.19 mmol/L) and no effect on triglycerides and HDL cholesterol when substituting soy drink for cow milk [12]. In agreement, another meta-analysis of RCTs found that soy drink consumption reduced total cholesterol (–0.23 mmol/L) and LDL cholesterol (–0.24 mmol/L), while showing no effects on triglycerides or HDL cholesterol [13].

Beyond soy, also oat drinks, demonstrated favorable effects, lowering total cholesterol (–0.12 mmol/L) compared with cow milk, although no consistent effects were observed for other blood lipid parameters.

Evidence from observational studies is limited, but available data indicate that soy drink is mainly not associated with CVD risk such as coronary artery disease and stroke >30 y of follow-up [[59], [60], [61]], whereas milk intake has also shown little or no associations in large cohort studies [62].

#### Blood pressure

Our analysis found that consumption of soy drinks compared with cow milk led to a reduction of systolic (∼8 mmHg) and diastolic (∼8 mmHg in the main analysis, and ∼4 mmHg in the sensitivity analysis) blood pressure. This is in line with a recent meta-analysis of soy drink compared with cow milk which reported a moderate reduction in both systolic (∼8 mmHg) and diastolic blood pressure (∼5 mmHg) [12]. Although these findings were accompanied by substantial heterogeneity regarding the magnitude of effect and should therefore be interpreted with caution. Similarly, Sohouli et al. [13], pooled 5 RCTs and found decreases in systolic (∼7 mmHg) and diastolic blood pressure (∼4 mmHg) after soy drink consumption, again with very high statistical heterogeneity. Taken together, these meta-analyses suggest a potential blood pressure–lowering effect of soy drink, but inconsistency across trials limits firm conclusions regarding the magnitude of effect. To date, however, no observational studies have specifically examined the association between plant-based drinks, including soy drink and blood pressure. For milk, findings from large cohort studies consistently suggest trivial beneficial associations with hypertension risk >15 y of follow-up [62].

#### Glycemic control

Plant-based drinks, including soy, showed no clear effects compared with cow milk on fasting glucose in our analysis. This is consistent with the systematic review by Erlich et al. [12], who found that substituting soy drink for cow milk had no effect on HbA1c, fasting plasma glucose, 2-h plasma glucose, or fasting insulin, with no meaningful interactions by added sugars. Likewise, a meta-analysis by Sohouli et al. [13], detected no effects of soy drink on fasting glucose or fasting insulin across available RCTs, with little between-study heterogeneity. Observational evidence on glycemic outcomes remains inconsistent: although some cohort studies have reported an inverse association between soy drink consumption and type 2 diabetes risk [63,64], others found no relationship [65]. For cow milk, by contrast, a comprehensive umbrella review indicated an inverse association with type 2 diabetes incidence [62]. For oat and rice drinks, evidence on glycemic outcomes remains scarce, highlighting a major research gap.

### Possible biological mechanisms

#### Soy drinks

The lipid-lowering properties of soy drinks are thought to be driven by their protein fraction, particularly the 7S globulin, which appears to inhibit apolipoprotein B synthesis [66], and enhances hepatic LDL receptor expression and thereby promotes clearance of LDL particles from the circulation [67]. Isoflavones in soy products may have further cardiometabolic benefits through several mechanisms: altering lipid and apolipoprotein profiles, reducing inflammation, reducing oxidative stress, decreasing oxidation of LDL cholesterol particles, and inducing nitric oxide (NO) [68,69]. Other bioactive components of soy, such as fiber and specific fatty acids, may contribute additional modest effects on cholesterol metabolism and bile acid turnover [44,66,68]. The blood pressure–reducing effects from soy might be related to isoflavones and their metabolites. Genistein and equol showed diuretic and vasorelaxing activities in in vivo studies [70,71]. Additionally, there is evidence that soy isoflavones may interfere with renin–angiotensin–aldosterone system and could thereby have blood pressure–lowering effects. Another mechanism of soy isoflavones may involve the ability to activate endothelial NO-synthase and thereby enhance NO production [72]. Although the content of soy isoflavones in soy drinks varies depending on production techniques, they may contain relevant concentrations [73,74].

#### Oat drinks

The favorable lipid effects of oat drinks are largely attributable to their β-glucan content. By forming a viscous gel in the intestine, β-glucans reduce bile acid reabsorption and enhance fecal cholesterol excretion [75]. In addition, they slow carbohydrate digestion and absorption, which may improve postprandial glucose responses, although such effects were not consistently demonstrated in the limited RCTs available. Conflicting results may be explained by differences in β-glucan content. The amount present in oat-based drinks can vary substantially depending on processing methods and the water content [76,77].

#### Rice drinks

Rice drinks are generally low in protein and lack significant bioactive compounds. Their carbohydrate quality may even be less favorable compared with soy and oat drinks, explaining the absence of measurable metabolic benefits [78].

#### Cow milk compared with plant-based drinks

Cow milk provides high-quality protein and calcium, but also contains saturated fats which may unfavorably impact LDL cholesterol. However, a recent scoping review of systematic reviews indicated that milk consumption was associated with either decreased or neutral associations on cardiovascular outcomes [79]. Nevertheless, Kiesswetter et al. [80] reported an inverse association with CVD when dairy products are substituted with plant foods (e.g., whole grains, nuts, legumes, avocado, or olive oil), indicating a further benefit of plant-based foods. Only 1 study used full-fat cow milk, and its findings were consistent with those from trials using low-fat cow milk. We did not perform a sensitivity analysis by fat content of cow milk, as an NMA showed no detrimental effects of higher dairy intake on blood lipids and blood pressure irrespective of fat content [81]. Therefore, we would not expect different results from conducting such sensitivity analysis. Substituting cow milk with soy or oat drinks may therefore shift macronutrient intake toward a more favorable profile, particularly when the plant-based drinks are fortified with micronutrients such as calcium [82].

### Implications

Our findings have practical implications given the rapid increase in plant-based consumption. Consumers often choose these products for ethical, environmental, or health-related reasons. From a health perspective, soy drinks show beneficial effects on LDL cholesterol and blood pressure, whereas oat drinks may also contribute to cholesterol reduction. For rice drinks, evidence suggests little or no difference compared with cow milk, and no adverse effects were detected for anthropometric outcomes. Overall, this indicates that soy-, oat-, and rice drinks can serve as acceptable substitutes for cow milk without major health disadvantages.

However, heterogeneity among plant-based drinks must be emphasized. Nutritional profiles differ widely, particularly regarding protein content, fortification with calcium and vitamin B_12_, and the presence of added sugars [12]. Policymakers and health professionals should highlight that not all plant-based drinks are nutritionally equivalent.

Beyond health, environmental and sustainability aspects are key. A recent scoping review highlighted that plant-based drinks generally show lower greenhouse gas emissions and reduced resource use compared with dairy, reinforcing their role as viable alternatives when nutrient fortification is adequate [83].

### Strengths and limitations

Strengths of our systematic review and NMA include a comprehensive search strategy across multiple databases and trial registries, prespecified eligibility criteria, RoB assessment, and rating the certainty of evidence with the GRADE approach. The usage of NMA allowed integration of direct and indirect comparisons, enabling a simultaneous analysis of different plant-based drinks and cow milk. An additional strength is the usage of harmonized doses of 500 mL/d and the application of dose–response NMA, which improves the interpretability of the findings.

However, several limitations must be considered. First, the number and size of available RCTs were small, limiting statistical power. Second, intervention duration was short (mostly 4–6 wk), and although our protocol initially aimed to investigate also children and adolescents and clinical endpoints such as CVD, type 2 diabetes, and cancer, no such data were available. Third, dietary adherence was mostly assessed by food records which are prone to measurement error and could additionally influence the results. Fourth, RoB was generally rated as *high* or *some concerns*, reflecting methodological shortcomings including inadequate wash-out periods in cross-over trails, randomization and selective reporting. Fifth, only 3 types of plant-based drinks—soy, oat, and rice—could be included. The existing evidence was almost exclusively based on comparisons of soy compared with cow milk, with very few trials directly comparing different plant-based drinks with each other. Additionally, no data were available for other commonly consumed plant-based drinks such as almond, pea, coconut, or other nut-based alternatives. Sixth, nutritional composition of plant-based drinks varied between trials and often was not fully reported, making it difficult to distinguish between commercially available products. Additionally, the content of relevant bioactive compounds such as isoflavones and β-glucan varies substantially depending on production techniques.

In conclusions, in this systematic review and NMA, 14 RCTs including a total of 543 participants were analyzed. The results indicate limited evidence for cardiometabolic differences between plant-based drinks and cow milk. However, soy drink may lower blood pressure and LDL cholesterol, whereas oat drinks may slightly improve total cholesterol compared with cow milk. Rice drinks showed no effects. No adverse effects of plant-based drinks were observed across outcomes. With appropriate fortification, oat and soy drinks could serve as nutritionally adequate alternatives to cow milk. Notably, the certainty of evidence was low to very low. Larger and longer multiarm RCTs are required to examine also commercially available plant drinks (i.e., oat, rice, and almond), with clear differentiation between fortified and unfortified products. In addition, studies including children and adolescents and evaluating long-term clinical endpoints are needed to better clarify the health effects of plant drinks.

## Authors' contributions

The authors’ responsibilities were as follows – LS, SW: designed the research and are the guarantors; EK, ES, JB, JS, SW: conducted the literature search and literature screening; JB, SW: extracted the data; EK, JS, SW: assessed the risk of bias of the included publications; JS, SW: evaluated the certainty of evidence; LS, MP, SW: analyzed the data and wrote the first draft of the paper; and all authors: interpreted the data, read the manuscript, and approved the final version.

## Data availability

This manuscript makes use of publicly available data from published studies; therefore, no original data are available for sharing.

## Funding

This project is funded by the Bavarian State Ministry of Food, Agriculture, Forestry and Tourism [Bayerische Staatsministerium für Ernährung, Landwirtschaft, Forsten und Tourismus (StMELF)].

## Deviations from protocol

None.

## Conflict of interest

MP reports financial support was provided by German Research Foundation (grant number: 504730171). TM reports a relationship with Scientific Advisory Board for Senior Nutrition of the State Association for Health of Saxony-Anhalt that includes: board membership. HH reports a relationship with Oviva Germany AG that includes: board membership. HH is a member of the Food-based Dietary Guidelines working group of the German Nutrition Society. LS is an Associate Editor for *Advances in Nutrition* and a member of the Grading of Recommendations, Assessment, Development and Evaluations working group. All other authors report no conflicts of interest.
