# Supplementary material for: Impact of Plant-Based Drinks on Cardiometabolic Outcomes: A Systematic Review and Network Meta-analysis
Source: Adv Nutr. 2026 Feb 4;17(3):100595. doi: 10.1016/j.advnut.2026.100595 (PMC12955114; doi:10.1016/j.advnut.2026.100595)
Supplement: multimedia component 1 [file mmc1.docx]

**Impact of plant-based drinks on cardiometabolic outcomes: a systematic review and network meta-analysis**

Sabina Wallerer, Julia Stadelmaier, Maria Petropoulou, Eva Kiesswetter, Jaqueline Beck, Elida Sina, Toni Meier, Kathrin Sedlmaier, Martin Kussmann, Hans Hauner, Lukas Schwingshackl

**Data supplement**

Content

[Supplemental Table 1. Eligibility Criteria 5](#_Toc214535773)

[Supplemental Table 2. List of excluded studies 6](#_Toc214535774)

[Supplemental Table 3. Overview of interventions of included studies 7](#_Toc214535775)

[Supplemental Table 4. League table for the outcome body weight (kg) comparing all plant-drinks and cow’s milk 13](#_Toc214535776)

[Supplemental Table 5. GRADE assessment of direct, indirect and network estimates for the outcome body weight (kg) comparing all plant-drinks and cow’s milk 13](#_Toc214535777)

[Supplemental Table 6. GRADE assessments for pairwise meta-analyses comparing soy drink and cow’s milk 15](#_Toc214535778)

[Supplemental Table 7. League table for the outcome LDL-cholesterol (mmol/L) comparing all plant-drinks and cow’s milk 17](#_Toc214535779)

[Supplemental Table 8. GRADE assessment of direct, indirect and network estimates for the outcome LDL-cholesterol (mmol/L) comparing all plant-drinks and cow’s milk 17](#_Toc214535780)

[Supplemental Table 9. League table for the outcome HDL-cholesterol (mmol/L) comparing all plant-drinks and cow’s milk 19](#_Toc214535781)

[Supplemental Table 10. GRADE assessment of direct, indirect and network estimates for the outcome HDL-cholesterol (mmol/L) comparing all plant-drinks and cow’s milk 19](#_Toc214535782)

[Supplemental Table 11. League table for the outcome triglycerides (mmol/L) comparing all plant-drinks and cow’s milk 21](#_Toc214535783)

[Supplemental Table 12. GRADE assessment of direct, indirect and network estimates for the outcome triglycerides (mmol/L) comparing all plant-drinks and cow’s milk 21](#_Toc214535784)

[Supplemental Table 13. League table for the outcome total cholesterol (mmol/L) comparing all plant-drinks and cow’s milk 23](#_Toc214535785)

[Supplemental Table 14. GRADE assessment of direct, indirect and network estimates for the outcome total cholesterol (mmol/L) comparing all plant-drinks and cow’s milk 23](#_Toc214535786)

[Supplemental Table 15. League table for the outcome fasting blood glucose (mmol/L) comparing all plant-drinks and cow’s milk 25](#_Toc214535787)

[Supplemental Table 16. GRADE assessment of direct, indirect and network estimates for the outcome fasting blood glucose (mmol/L) comparing all plant-drinks and cow’s milk 25](#_Toc214535788)

[Supplemental Table 17. Relative ranking of plant-drinks and cow milk (P-scores*) 27](#_Toc214535789)

[Supplemental Table 18. Heterogeneity measures design-by-treatment statistics for NMA comparing different plant-drinks and cow’s milk 27](#_Toc214535790)

[Supplemental Table 19. League table for the sensitivity analysis with post values only 28](#_Toc214535791)

[Supplemental Table 20. League table for the sensitivity analysis with post values only 28](#_Toc214535792)

[Supplemental Table 21. League table for the sensitivity analysis with change scores only 28](#_Toc214535793)

[Supplemental Table 22. League table for the sensitivity analysis with change scores only 29](#_Toc214535794)

[Supplemental Table 23. League table for the sensitivity analysis with change scores only 29](#_Toc214535795)

[Supplemental Table 24. League table for the sensitivity analysis with change scores only 29](#_Toc214535796)

[Supplemental Table 25. League table for the sensitivity analysis with change scores only 30](#_Toc214535797)

[Supplemental Table 26. League table for the sensitivity analysis with change scores only 30](#_Toc214535798)

[Supplemental Table 27. Selected dose-response models for dose-response network meta-analyses 30](#_Toc214535799)

[Supplemental Figure 1. Risk of Bias assessment for parallel trial 31](#_Toc214535800)

[Supplemental Figure 2. Risk of Bias assessment for cross-over trials 31](#_Toc214535801)

[Supplemental Figure 3. Forest Plot summarizing mean differences with 95%CIs 32](#_Toc214535802)

[Supplemental Figure 4. Forest Plot summarizing mean differences with 95%CIs 32](#_Toc214535803)

[Supplemental Figure 5. Forest Plot summarizing mean differences with 95%CIs 33](#_Toc214535804)

[Supplemental Figure 6. Forest Plot summarizing mean differences with 95%CIs 33](#_Toc214535805)

[Supplemental Figure 7. Forest Plot summarizing mean differences with 95%CIs 34](#_Toc214535806)

[Supplemental Figure 8. Forest Plot summarizing mean differences with 95%CIs 35](#_Toc214535807)

[Supplemental Figure 9. Forest Plot summarizing mean differences with 95%CIs 36](#_Toc214535808)

[Supplemental Figure 10. Forest Plot summarizing mean differences with 95%CIs 37](#_Toc214535809)

[Supplemental Figure 11. Forest Plot summarizing mean differences with 95%CIs 38](#_Toc214535810)

[Supplemental Figure 12. Forest Plot summarizing mean differences with 95%CIs 38](#_Toc214535811)

[Supplemental Figure 13. Comparison adjusted funnel plot for LDL-cholesterol for the network meta-analysis comparing plant-drinks and cow’s milk 39](#_Toc214535812)

[Supplemental Figure 14. Comparison adjusted funnel plot for total cholesterol for the network meta-analysis comparing plant-drinks and cow’s milk 40](#_Toc214535813)

[Supplemental Figure 15. Subgroup Analyses from NMA comparing the effects different plant-drinks with cow’s milk on body weight (kg) 41](#_Toc214535814)

[Supplemental Figure 16. Subgroup Analyses from NMA comparing the effects different plant-drinks with cow’s milk on LDL-cholesterol (mmol/L) 42](#_Toc214535815)

[Supplemental Figure 17. Subgroup Analyses from NMA comparing the effects different plant-drinks with cow’s milk on HDL-cholesterol (mmol/L) 43](#_Toc214535816)

[Supplemental Figure 18. Subgroup Analyses from NMA comparing the effects different plant-drinks with cow’s milk on total cholesterol (mmol/L) 44](#_Toc214535817)

[Supplemental Figure 19. Subgroup Analyses from NMA comparing the effects different plant-drinks with cow’s milk on triglycerides (mmol/L) 45](#_Toc214535818)

[Supplemental Figure 20. Subgroup Analyses from NMA comparing the effects different plant-drinks with cow’s milk on fasting blood glucose (mmol/L) 46](#_Toc214535819)

[Supplemental Figure 21. Sensitivity analysis regarding risk of bias 47](#_Toc214535820)

[Supplemental Figure 22. Sensitivity analysis regarding risk of bias 47](#_Toc214535821)

[Supplemental Figure 23. Sensitivity analysis regarding risk of bias 48](#_Toc214535822)

[Supplemental Figure 24. Sensitivity analysis regarding risk of bias 48](#_Toc214535823)

[Supplemental Figure 25. Sensitivity analysis regarding risk of bias 49](#_Toc214535824)

[Supplemental Figure 26. Sensitivity analysis harmonized NMA with post values 49](#_Toc214535825)

[Supplemental Figure 27. Sensitivity analysis harmonized NMA with post values 50](#_Toc214535826)

[Supplemental Figure 28. Sensitivity analysis harmonized NMA with change scores only 50](#_Toc214535827)

[Supplemental Figure 29. Sensitivity analysis harmonized NMA with change scores only 51](#_Toc214535828)

[Supplemental Figure 30. Sensitivity analysis harmonized NMA with change scores only 51](#_Toc214535829)

[Supplemental Figure 31. Sensitivity analysis harmonized NMA with change scores only 51](#_Toc214535830)

[Supplemental Figure 32. Sensitivity analysis harmonized NMA with change scores only 52](#_Toc214535831)

[Supplemental Figure 33. Sensitivity analysis harmonized NMA with change scores only 52](#_Toc214535832)

[Supplemental Figure 34. Sensitivity analysis pairwise meta-analysis with change scores only 52](#_Toc214535833)

[Supplemental Figure 35. Sensitivity analysis pairwise meta-analysis with change scores only 53](#_Toc214535834)

[Supplemental Figure 36. Dose-response curve from dose-response network meta-analysis for body weight 54](#_Toc214535835)

[Supplemental Figure 37. Dose-response curve from dose-response network meta-analysis for LDL-Cholesterol 56](#_Toc214535836)

[Supplemental Figure 38. Dose-response curve from dose-response network meta-analysis for HDL-Cholesterol 58](#_Toc214535837)

[Supplemental Figure 39. Dose-response curve from dose-response network meta-analysis for total cholesterol 60](#_Toc214535838)

[Supplemental Figure 40. Dose-response curve from dose-response network meta-analysis for triglycerides 62](#_Toc214535839)

[Supplemental Figure 41. Dose-response curve from dose-response network meta-analysis for systolic blood pressure^1^ 64](#_Toc214535840)

[Supplemental Figure 42. Dose-response curve from dose-response network meta-analysis for diastolic blood pressure^1^ 66](#_Toc214535841)

[Supplemental Figure 43. Dose-response curve from dose-response network meta-analysis for fasting blood glucose 68](#_Toc214535842)

[Supplemental Appendix 1 Search Strategy 70](#_Toc214535843)

[Supplemental Appendix 2: Additional description and decision criteria for each domain in ROB 2 assessment (for parallel and cross-over trials) 74](#_Toc214535844)

[Supplemental Appendix 3: Additional description and decision criteria for each domain in the GRADE assessment.^a^ 79](#_Toc214535845)

[Supplemental Appendix 4: Additional description of dose-response patterns for all outcomes for dose-response network meta-analysis 82](#_Toc214535846)

[References 83](#_Toc214535847)

# Supplemental Table 1. Eligibility Criteria

|  | **Inclusion criteria** | **Exclusion criteria** |
| --- | --- | --- |
| **P** (population) | Generally healthy adults (≥ 18 years of age), children and adolescents (≥ 4 – 18 years of age). We included children aged ≥4 years, as nutritional behaviours and dietary intake patterns tend to be more stable and comparable than in earlier years. In addition, intervention settings (i.e., kindergarten, schools) become relevant at this age, enabling also more comparable study designs (1). Younger children (0–3 years) undergo rapid developmental changes that substantially affect diet and nutrient requirements, which would make synthesis across studies less reliable. | Studies focusing on pregnant women, malnourished patients, and patients with chronic diseases (e.g., nephropathy, rheumatism, cancer, cardiovascular disease, type 2 diabetes) or related allergies (e.g., casein, whey) will be excluded. |
| **I/E** (intervention/exposure)  **C** (comparison) | Intake/consumption of plant-based drinks (e.g., oat, soy, almond, rice)  Other plant-based drink, cow`s milk;  We allow for the presence of co-interventions (e.g., physical activity) as long as they are balanced across study arms within the respective RCT. | Other animal-based dairy alternatives (e.g., from sheep, goats), milk/protein isolates (e.g., casein, whey), formula, capsules, phytoestrogen fortified milk alternatives, and fermented milk products with additional microbiota strains added (beyond those naturally occurring) will be excluded.  Studies will be excluded if energy intakes are not similar between groups. |
| **O** (outcome) | Any of the following health-related outcomes:  - All-cause mortality;  - Cardiovascular diseases;  - Type 2 diabetes;  - Cancer;  - Anthropometric measurements: body  weight, fat mass, waist circumference,  BMI, weight-for-age, weight-for-height;  - Cardiometabolic risk factors: blood  pressure, blood glucose, HbA1c, total  cholesterol, triglycerides, LDL-  cholesterol, HDL-cholesterol;  - Bone health: bone mineral density |  |
| **S** (study design) | Randomized controlled trials (parallel and cross-over) with an intervention duration of minimum 3 weeks. |  |

BMI body mass index; HbA1c glycated hemoglobin; HDL high density lipoprotein; LDL low density lipoprotein; RCT randomized controlled trials;

# Supplemental Table 2. List of excluded studies

| **Reason for exclusion** | **References** |
| --- | --- |
| Wrong patient population (n = 4) | (2-5) |
| Wrong intervention/comparator (n = 41) | (6-46) |
| Wrong study design (n = 151) | (47-197) |
| Wrong outcome (n = 10) | (198-207) |
| Ongoing study (n = 3) | (208-210) |
| No full text published (n = 10) | (211-220) |

# Supplemental Table 3. Overview of interventions of included studies

| **Study Author**  **(Country, Year)** | **Interventions** | | | **Type of diet** | **Adherence to intervention** | **Degree of adherence** | **Outcomes** | **Study Funding / COI** |
| --- | --- | --- | --- | --- | --- | --- | --- | --- |
|  | **Arm 1** | **Arm 2** | **Arm 3** |  |  |  |  |  |
| Parallel Trials | | | | | | | |  |
| Beavers  (USA, 2010 (221)) | Cow´s milk  Dose: 3 servings/d (~700 ml/d) | Soy drink  Dose: 3 servings/d (~700 ml/d) | NA | Instructed to avoid dairy milk and limit dairy servings to two per day and to avoid isoflavone-containing soy products. Soy drink and cow’s milk were provided. ^a^ | 4-day dietary record at baseline and during the second half of the intervention period; compliance log of daily milk ingestion | Overall compliance rate of 98% (excluding one participant with < 80% compliance) | TG, TC, HDL-C, LDL-C | Study funding by WhiteWave Foods Inc. |
|  | Nutritional content (per 100 ml):  Energy: 51 kcal; FAT 1.9 g;  CHO 5.1 g;  PRO 3.4 g | Nutritional content (per 100 ml):  Energy: 55 kcal; FAT 1.7 g;  CHO 8.0 g;  PRO 2.5 g |  |  |  |  |  |  |
| Faghih  (Iran, 2011 (222)) | Cow´s milk  Dose: 3 servings/d (~660 ml/d); | Soy drink  Dose: 3 servings/d (~660 ml/d); | NA | Both diets provided a 500 kcal/d energy deficit. The diets were prescribed to contain: 55% CHO, 18% PRO and 27% FAT. Total Ca content of the milk diet and the soy  milk diet was between 1200 and 1300 mg/d. ^b^ | 24-h dietary recall | / | BW, BMI, WC, FM | Study funding by the National Nutrition and Food Technology Research Institute. |
|  | Nutritional content (per 100 ml):  FAT 1.5 g | Nutritional content (per 100 ml):  not specified;  Ca-fortified |  |  |  |  |  |  |
| Gui  (China, 2012 (223)) | Cow´s milk  Dose: 250 ml/d | Soy drink  Dose: 250 ml/d | NA | No statement regarding diet. Soy drink and cow’s milk were provided. | Participants recorded their milk or soy drink intake per day on compliance calendars, which were collected and checked every 3 months. | Overall mean reported milk compliance was 93.1% for cow’s milk and 82.5% for soy drink. | BMD | This study was supported by the International Atom Energy Agency (RAS6041). Bright Dairy & Food Co., Ltd. provided the milk and calcium-fortified soymilk used in the study. |
|  | Nutritional content (per 100 ml):  FAT 4 g,  lactose 4.9 g; casein 2.6 g;  Ca 100 mg | Nutritional content (per 100 ml):  FAT 1 g;  lactose 1 g;  soy-PRO 2.6 g; Ca 100 mg (fortified);  soy isoflavones 1.8 mg |  |  |  |  |  |  |
| Mitchell  (UK, 1999 (224)) | Cow´s milk  Dose: 1000 ml/d | Soy drink  Dose: 1000 ml/d | Rice drink  Dose: 1000 ml/d | No statement regarding diet. Soy drink and cow’s milk were provided. | / | | TC, TG | This study was supported by the Scottish Office Agriculture Environment and Fisheries Department and the Ministry of Agriculture, Fisheries and Food. |
|  | Nutritional content (per 100 ml):  Not specified | Nutritional content (per 100 ml): Not specified | Nutritional content (per 100 ml): Not specified |  |  |  |  |  |
| Önning  (Sweden, 1998 (225)) | Cow´s milk  Dose: 750 ml/d (women); 1000 ml/d (men) | Soy drink  Dose: 750 ml/d (women); 1000 ml/d (men) | Oat drink  Dose: 750 ml/d (women); 1000 ml/d (men)  Nutritional content (per 1000 ml): 421 kcal; FAT 15 g; CHO 54 g; PRO 15 g; dietary fiber 6.6 g; β-glucan 4.5 g | Frozen dishes (Findus, Nestlé, Bjuv, Sweden) were provided for dinner 5 days per week. The subjects were also asked to maintain their usual pattern of activity and try to keep a stable body weight.  Soy drink, Oat drink and cow’s milk were provided. | Detailed food record over the first period. Participants were then asked to repeat this food consumption as much as possible during the second period, so that the test milk would be the main dietary difference between the test periods. | / | BW, TC, HDL-C, LDL-C, TG, FBG | This study was supported by the Scanian Farmers` Research Foundation. B. Åkesson was supported by the Swedish Research Council for Forestry and Agriculture and I. Lundquist by the Swedish Medical Research Council (14X-4286). |
|  | Nutritional content (per 100 ml):  Energy 48 kcal; FAT 1.7 g;  CHO 4.9 g;  PRO 3.4 g | Nutritional content (per 100 ml):  Energy 45 kcal; FAT 2.0 g;  CHO 4.0 g;  PRO 3.0 g;  dietary fiber 0.6 g |  |  |  |  |  |  |
| Rivas  (Spain, 2002 (226)) | Cow´s milk  Dose: 1000 ml/d | Soy drink  Dose: 1000 ml/d | NA | Subjects were asked not to change dietary habits and consume a Mediterranean diet. Soy drink and cow’s milk were provided. | / | / | SBP, DBP | This study was partly supported by the Direccion General de Investigacion Cientifica y Tecnica of Spain (PM96-0086). |
|  | Nutritional content (per 100 ml):  Energy 42 kcal; FAT 0.2 g;  CHO 2.5 g;  PRO 1.6 g;  Ca 60 mg | Nutritional content (per 100 ml):  Energy 53 kcal; FAT 1.1 g;  CHO 1.4 g;  PRO 1.8 g;  Ca 60 mg; genistein 8 mg; daidzein 6 mg |  |  |  |  |  |  |
| **Study Author**  **(Country, Year)** | **Interventions** | | | **Type of diet** | **Adherence to intervention** | **Degree of adherence** | **Outcomes** | **Study Funding / COI** |
|  | **Arm 1** | **Arm 2** | **Arm 3** |  |  |  |  |  |
| Cross-Over Trials | | | | | | | |  |
| Azadbakht  (Iran, 2011 (227)) | Cow´s milk  Dose: 240 ml/d | Soy drink  Dose: 240 ml/d | NA | Energy restriction of 200 to 500 kcal/d for each participant based on BMI range. Both diets included macronutrient composition of 50-60% CHO, 15-20% PRO, < 30% FAT, and < 5% of caloric intake from simple sugars.  Diet was prescribed, only soy drinks were provided. ^c^ | Patient adherence was assessed in terms of attendance at every two-week visit and through analysis of the 3-d food diaries. | No significant difference between the prescribed. amount and amount consumed. | BW, BMI, WC, SBP, DBP | / |
|  | Nutritional content (per 100 ml):  Not specified | Nutritional content (per 100 ml):  FAT: 1.0 g;  CHO: 3.5 g;  Sugar 2 g;  Protein 2 g;  Ca 40 mg;  Na 40 mg; |  |  |  |  |  |  |
| Bicarello  (Brazil, 2004 (228)) | Cow´s milk  Dose: 1000 ml/d | Soy drink  Dose: 1000 ml/d | NA | Before the first intervention period all patients were asked to consume a TLC diet (Therapeutic Lifestyle Changes); consists of 15% PRO, 50% to 60% CHO, less than 7% SFA, no more than 10% PUFA, no more than 20% MUFA, less than 200 mg/d of chol and 20 to 30 g/d of viscous (soluble) fiber.  Diet was prescribed; soy drink and cow’s milk were provided. ^d^ | Patients were carefully monitored with regard to diet and milk consumption and were asked to bring the label with a code written at the top of each 1000 mL of tetra-pack milk. Dietary records were collected during the last 3 d (including 1 d of the weekend) of each treatment period (at 0, 6, and 12 wk). | Analysis of dietary questionnaires showed good overall compliance with the recommended diets. | TG, TC, HDL-C, LDL-C | One researcher was supported by a CAPES grant. Unilever Bestfoods of Brazil provided the cow`s milk and soy drink used in the study. |
|  | Nutritional content (per 100 ml):  Energy 28 kcal;  FAT < 0.01 g;  CHO 4.3 g;  PRO 2.8 g;  fiber < 0.01 g;  chol < 1 mg;  Ca 89 mg;  Fe < 0.1 mg;  Vit. A < 1.5 µg;  Na 28 mg | Nutritional content (per 100 ml):  Energy 30 kcal; FAT 1.8 g;  CHO 0.5 g;  PRO 3.0 g;  fiber 0.13 g;  chol 0 g;  Ca 9 mg;  Fe 0.3 mg;  Vit. A 60 µg;  Na 43 mg; genistein 5 mg; daidzein 3.4 mg; glycetein 0.5 mg |  |  |  |  |  |  |
| Gardner^1^  (USA, 2007 (229)) | Cow´s milk  Dose: 547 ml/d | Soy drink  Dose: 946 ml/d | NA | Instructions to follow a heart-healthy diet based on American Heart Association guidelines.  Soy drink and cow’s milk were provided. ^e^ | Participants kept daily milk consumption logs and submitted them weekly during each of the three 4-wk milk consumption phases. Three-day food records were completed at five time points during the study: baseline (prior to starting the run-in phase), end of the 4-wk run-in phase, and once in the middle of each of the three milk phases. | Ninety percent or more of the total assigned milk intake was consumed by 100% and 89% of the participants during the soy drink and cow’s milk phases, respectively. | TC, HDL-C, LDL-C, TG, FBG | This study was supported by unrestricted gift funds from WhiteWave Foods Inc., by the National Cancer Institute and the National Institutes of Health (CA71789), and by the Human Health Service (M01-RR00070). M. Messina has provided consulting services to WhiteWave Foods Inc. and C.D. Gardner received the unrestricted gift funds that were used for this trial. |
|  | Nutritional content (per 100 ml):  Energy 55 kcal; FAT 1.1 g;  CHO 6.4 g;  sugar 6.4 g;  PRO 4.6 g;  chol 4.2 mg;  fiber 0 g; isoflavones 0 g | Nutritional content (per 100 ml): Energy 42 kcal; FAT 1.4 g;  CHO 4.2 g;  sugar 3.0 g;  PRO 2.6 g;  chol 0 mg;  fiber 0.4 g; isoflavones 13.2 mg |  |  |  |  |  |  |
| Keshavarz  (Iran, 2012 (230)) | Cow´s milk  Dose: 240 ml/d | Soy drink  Dose: 240 ml | NA | Energy restriction of 200 to 500 kcal/day; both diets should consist of macronutrient composition as follows: 50‑60% CHO, 15‑20% PRO, <30% FAT.  Diet was prescribed; Soy drinks and cow’s milk were provided. ^f^ | Patients were visited every two weeks and their adherence was checked by analysis of the three‑day food diaries at baseline and end of each trial. Macronutrients and servings of different food group intake separately in each dietary period measurement. | There were no differences  between the prescribed amount and reported  dietary intake. | BW, BMI, WC, SBP, DBP, FBG, TG, TC, HDL-C, LDL-C | Study funding by the Teheran University of Medical Sciences and Food Security Research Center and the Isfahan University of Medical Sciences (289249). |
|  | Nutritional content (per 100 ml):  FAT 1.5 g;  CHO 4.9 g;  PRO 3.3 g;  Na 50 mg;  K 156 m;  P 120 mg;  Ca 100 mg | Nutritional content (per 100 ml):  FAT 1 g;  CHO 3.5 g;  PRO 2.5 g;  Na 40 mg;  K 110 mg;  P 53 mg;  Ca 40 mg |  |  |  |  |  |  |
| Önning  (Sweden, 1999 (231)) | Oat drink  Dose: 750 ml/d | Rice drink  Dose: 750 ml/d | NA | Participants were asked to consume their usual diet. Oat and rice drinks were provided. | / | / | BW, TC, HDL-C, LDL-C, TG, FBG | This study was supported by the Scanian Farmers` Research Foundation. |
|  | Nutritional content (per 100 ml):  Energy 63 kcal; FAT 1.3 g;  CHO 12 g;  PRO 0.7 g;  fiber 1.1 g;  maltose 3.9 g; glucose 0.6 g | Nutritional content (per 100 ml):  Energy 61 kcal; FAT 0.9 g;  CHO 13 g;  PRO 0.2 g;  fiber 0.2 g; maltose 5.9 g; glucose 0.8 g |  |  |  |  |  |  |
| Sirtori  (Italy, 2002 (232)) | Cow´s milk  Dose: 500 ml/d | Soy drink  Dose: 500 ml/d | NA | Controlled diet (prescribed), Soy drink and cow’s milk were provided. | The food intake was assessed by 24-h recordings, using a computerized system adjusted to the Italian dietary intake (Dietosystem for Windows; Dietosystem, Milan, Italy). | The patients did not generally deviate by more than 10–15% in their 3-day recordings. | TC, LDL-C | This study was partially supported by the Consiglio Nazionale delle Ricerche of Italy and by ALPRO. |
|  | Nutritional content (per 100 ml):  Not specified | Nutritional content (per 100 ml):  Energy 54 kcal; FAT 2.9 g;  CHO 1.9 g;  sugar 1.6 g;  PRO 5 g;  fiber 2.9 g;  Na 10 mg;  P 80 mg;  Ca 12 mg;  Vit. A 120 µg;  Vit. C 9 mg;  Vit. E 1.5 mg |  |  |  |  |  |  |
| Sirtori  (Italy, 1999 (233)) | Cow´s milk  Dose: 500 ml/d | Soy drink  Dose: 500 ml/d | NA | For 1400 kcal/d: Diet provided (% energy): CHO 56.7, FAT 25.1 (P:S 2.75 for soya milk, 2.65 for cows’ milk), PRO 18.2.  For 2000 kcal/d: Diet provided (% energy): CHO 59.9, FAT 24.3 (P:S 2.75 for soya milk, 2.65 for cows’ milk), PRO 15.8.  Controlled diet (prescribed), Soy drink and cow’s milk were provided. | Daily food records, indicating the consumption of test diet foods and any food items that were not part of the therapeutic diet, along with the number of soya or cows’ milk cartons taken daily. Dietary records were collected during the last 3 d of each treatment period, and were analysed for nutrient intake by the Dietosystem for Windows Program (DIETOSYSTEM, Milan, Italy), specifically tailored for the Italian food items. | The patients did not generally deviate by more than 10–15% in their 3-day recordings. | TC, LDL-C | This study was partly supported by the Consiglio Nazionale delle Ricerche of Italy. |
|  | Nutritional contents (per 100 ml):  Energy 54 kcal; FAT 4.4 g; digestible sugars 3.0 g;  soluble indigestible sugars 5.5 g;  PRO 14 g (fortified) | Nutritional content (per 100 ml):  Energy 54 kcal; FAT 4.4 g; digestible sugars 3.0 g;  soluble indigestible sugars 5.5 g;  PRO 14 g (fortified); genistein 8 mg/l;  daidzein 4 mg/l;  glycetin 2.7 mg/d; total isoflavones 6 mg/l |  |  |  |  |  |  |
| Steele  (Australia, 1992 (234)) | Cow´s milk  Dose: 660 ml/d (group A); 774 ml/d (group B) | Soy drink  Dose: 622 ml/d (group A); 787 ml/d (group B) | NA | The subjects were requested to avoid the use of purchased foods containing milk products such as cheese, ice cream, desserts, soups, dressings, for the duration of the study. Modified free choice diet (vegan, vegetarian, Omnivore).  Only soy drink was provided. | Milk or soy drink record was supplied to each subject to determine compliance | Recommended consumption of > 500 ml/d was achieved. | TC, HDL-C, LDL-C, TG | Study funding by Sanitarium Health Food Company. |
|  | Nutritional content (per 100 ml):  Energy 34 kcal; FAT 0.1 g;  CHO 4.7 g;  PRO 3.3 g;  Ca 119 mg;  P 90 mg;  chol 3 mg | Nutritional content (per 100 ml):  Energy 62 kcal; FAT 3.4 g;  CHO 4.7 g;  PRO 3.4 g;  Ca 116 mg;  P 92 mg;  Vit. A 40 mg;  fortified with vitamins, minerals and oil; |  |  |  |  |  |  |

^1^ Gardner et al. assigned intervention doses to achieve equal protein content.

^a^ Macronutrients consumed during the trial (mean ± SD): Arm 1: fat: 77.2 ± 25 g, CHO: 216.5 ± 67 g, fiber: 16 ± 6 g, PRO: 90.9 ± 22 g, Arm 2: fat: 67.3 ± 23 g, CHO: 208.9 ± 55 g, fiber: 26.7 ± 5 g, PRO: 94.7 ± 23 g; ^b^ Macronutrients consumed during the trial (mean ± SD): Arm 1: fat: 27.4 ± 3.2%, CHO: 55.0 ± 4.1%, fiber: 13.8 ± 2.4 g, PRO: 17.6 ± 2.2%, Arm 2: fat: 26.7 ± 2.2%, CHO: 55.0 ± 2.2%, fiber: 14.7 ± 2.2 g, PRO: 17.8 ± 1.1%; ^c^ Macronutrients consumed during the trial (mean ± SE): Arm 1: fat: 32.7 ± 1.2 %, CHO: 53.3 ± 1.1%, fiber: 20 ± 0.6 g, PRO: 14 ± 0.3%, Arm 2: fat: 31.7 ± 1.2%, CHO: 54.2 ± 1.1%, fiber: 17.5 ± 0.1 g, PRO: 14.1 ± 0.3%; ^d^ Macronutrients consumed during the trial (mean ± SE): Arm 1: fat: 25.0 ± 1.0%, CHO: 53.7 ± 0.9%, fiber: 13 ± 1.0 g, PRO: 20.9 ± 0.5%, Arm 2: fat: 34 ± 1.0%, CHO: 45.0 ± 1.1%, fiber: 16.0 ± 1.0 g, PRO: 20.7 ± 0.6 g; ^e^ Macronutrients consumed during the trial (mean ± SD): Arm 1: fat: 71 ± 23 g, CHO: 237 ± 71 g, fiber: 18 ± 7 g, PRO: 100 ± 18 g, Arm 2: fat: 79 ± 29 g, CHO: 234 ± 72 g, fiber: 22 ± 7 g, PRO: 96 ± 20 g; ^f^ Macronutrients consumed during the trial (mean ± SE): Arm 1: fat: 30.0 ± 1.3 %, CHO: 52.6 ± 1.3%, fiber: 16.8 ± 1.0 g, PRO: 16.8 ± 0.6%, Arm 2: fat: 31.0 ± 1.1%, CHO: 51.9 ± 1.2%, fiber: 17.6 ± 1.0 g, PRO: 16.9 ± 0.6%;

/ not stated; BMD bone mineral density; BMI body mass index; BW body weight; Ca calcium; CHO carbohydrates; chol cholesterol; COI conflicts of interest; d day; DBP diastolic blood pressure; FAT fat; FBG fasting blood glucose; Fe iron; FM fat mass; h hour; HDL-C high density lipoprotein cholesterol; K potassium; LDL-C low density lipoprotein cholesterol; NA not applicable; Na sodium; P phosphorus; PRO protein; SBP systolic blood pressure; SE standard error; SD standard deviation; TC total cholesterol; TG triglycerides; Vit. A vitamin A; Vit. C vitamin C; Vit. E vitamin E; WC waist circumference; wk week;

# Supplemental Table 4. League table for the outcome body weight (kg) comparing all plant-drinks and cow’s milk

| **Soy drink** | -0.06 [-0.69, 0.58] | NA | 0.53 [-0.32, 1.39] |
| --- | --- | --- | --- |
| 0.08 [-0.46, 0.63] | **Oat drink** | 0.07 [-2.82, 2.95] | 0.06 [-0.58, 0.69] |
| 0.15 [-2.79, 3.08] | 0.07 [-2.82, 2.95] | **Rice drink** | NA |
| 0.28 [-0.34, 0.90] | 0.20 [-0.35, 0.74] | 0.13 [-2.81, 3.06] | **Cow’s milk** |

Mean differences (MD) are computed from the main network meta-analysis (NMA) with harmonized doses (500 ml/d) using post- and change scores. The upper triangle describes the MD and 95% CI from direct comparison; the lower triangle displays the MD and 95% CI computed from the NMA. For the upper triangle, MDs < 0 favor the row-defining treatment. For the lower triangle, MDs < 0 favor the column-defining treatment.

The heterogeneity standard deviation (τ) was estimated at 0.

# Supplemental Table 5. GRADE assessment of direct, indirect and network estimates for the outcome body weight (kg) comparing all plant-drinks and cow’s milk

|  |  | **Direct evidence** | | | **Indirect evidence** | | **Network Meta-Analysis** | |
| --- | --- | --- | --- | --- | --- | --- | --- | --- |
| **Comparison** | **N studies** | **Proportion direct evidence** | **MD (95%CI)** | **Certainty of evidence** | **MD (95%CI)** | **Certainty of evidence** | **MD (95%CI)** | **Certainty of evidence** |
| **Soy drink vs. oat drink** | 1 | 74 | -0.06 [-0.69, 0.58] | ⨁⨁◯◯^1^ | 0.47 [-0.59, 1.54] | ⨁⨁◯◯ | 0.08 [-0.46, 0.63] | ⨁◯◯◯^2^ |
| **Soy drink vs. rice drink** | 0 | 0 | NA | NA | 0.15 [-2.79, 3.08] | ⨁⨁◯◯ | 0.15 [-2.79, 3.08] | ⨁◯◯◯^3^ |
| **Soy drink vs. cow’s milk** | 3 | 52 | 0.53 [-0.32, 1.39] | ⨁⨁⨁◯^4^ | 0.00 [-0.90, 0.90] | ⨁⨁◯◯ | 0.28 [-0.34, 0.90] | ⨁⨁◯◯^2^ |
| **Oat drink vs. rice drink** | 1 | 100 | 0.07 [-2.82, 2.95] | ⨁⨁◯◯^1^ | NA | NA | 0.07 [-2.82, 2.92] | ⨁◯◯◯^3^ |
| **Oat drink vs. cow’s milk** | 1 | 74 | 0.06 [-0.58, 0.69] | ⨁⨁◯◯^1^ | 0.59 [-0.48, 1.65] | ⨁⨁◯◯ | 0.20 [-0.35, 0.74] | ⨁◯◯◯^2^ |
| **Rice drink vs. cow’s milk** | 0 | 0 | NA | NA | 0.13 [-2.81, 3.06] | ⨁⨁◯◯ | 0.13 [-2.81, 3.06] | ⨁◯◯◯^3^ |

95%CI 95% confidence interval, MD mean difference, MID minimally important difference; NA not applicable, RoB risk of bias, ⨁⨁⨁⨁ high, ⨁⨁⨁◯ moderate; ⨁⨁◯◯ low, ⨁◯◯◯ very low

Explanations

^1^downgraded by two levels for RoB: More than 2/3 of the studies (and their contributing weight) were rated with a high RoB. No subgroup analysis for RoB could be conducted to test to robustness of the effect estimates. Specified for the present comparison: 1/1 study with high RoB.

^2^downgraded by one level for imprecision: The point estimate suggests a trivial effect, but the 95% CI includes the possibility of an important harm. (MID for body weight: ±0.5 kg)

^3^downgraded by one level for imprecision: The point estimate suggests a trivial effect, but the 95% CI includes the possibility of both important benefit and important harm. (MID for body weight: ±0.5 kg)

^4^downgraded by one level for RoB: less than 2/3 of the studies (and their contributing weight) were rated with a low RoB; 2/3 of the studies (but less than 2/3 of their contributing weight) were rated with a high RoB. Specified for the present comparison: 2/3 studies with high RoB (1.1% high RoB).

# Supplemental Table 6. GRADE assessments for pairwise meta-analyses comparing soy drink and cow’s milk

| **Certainty assessment** | | | | | | | | **№ of patients** | | **Effect** | | **Certainty** |
| --- | --- | --- | --- | --- | --- | --- | --- | --- | --- | --- | --- | --- |
| **№ of studies** | **Study design** | **Risk of bias** | **Inconsistency** | **Indirectness** | **Imprecision** | **Other considerations** | | **Soy drink** | **Cow drink** | **Relative (95%CI)** | **Absolute (95% CI)** |  |
| **BMI** | | | | | | |  |  |  |  |  |  |
| 3 | randomised trials | serious^1^ | not serious | not serious | serious^2^ | none | | 85 | 84 | - | MD **0.3 kg/m² lower** (0.57 lower to 0.02 lower) | ⨁⨁◯◯ Low^1,2^ |
| **Waist Circumference** | | | | | | |  |  |  |  |  |  |
| 3 | randomised trials | serious^3^ | not serious | not serious | serious^4^ | none | | 85 | 84 | - | MD **0.34 cm higher** (0.59 lower to 1.27 higher) | ⨁⨁◯◯ Low^3,4^ |
| **Systolic Blood Pressure** | | | | | | |  |  |  |  |  |  |
| 3 | randomised trials | serious^5^ | not serious | not serious | serious^6^ | none | | 83 | 83 | - | MD **8.23 mmHg lower** (10.9 lower to 5.55 lower) | ⨁⨁◯◯ Low^5,6^ |
| **Diastolic Blood Pressure** | | | | | | |  |  |  |  |  |  |
| 3 | randomised trials | serious^7^ | not serious | not serious | serious^6^ | none | | 83 | 83 | - | MD **7.82 mmHg lower** (13.61 lower to 2.02 lower) | ⨁⨁◯◯ Low^6,7^ |

95%CI 95% confidence interval, MD mean difference, MID minimally important difference; OIS optimal information size; RoB risk of bias, ⨁⨁⨁⨁ high, ⨁⨁⨁◯ moderate; ⨁⨁◯◯ low, ⨁◯◯◯ very low

**Explanations**

1. downgraded by 1 level for RoB: less than 2/3 of the studies (and their contributing weight) were rated with a low RoB; 2/3 of the studies (but less than 2/3 of their contributing weight) were rated with a high RoB. Specified for the present comparison: 2/3 studies with high RoB (1.1% high RoB).

2. downgraded by 1 level for imprecision, as the 95% confidence interval overlaps the MID for BMI, which was set at ±0.2 kg/m2.

3. downgraded by 1 level for RoB: less than 2/3 of the studies (and their contributing weight) were rated with a low RoB; 2/3 of the studies (but less than 2/3 of their contributing weight) were rated with a high RoB. Specified for the present comparison: 2/3 studies with high RoB (1.7% high RoB).

4. downgraded by 1 level for imprecision, the confidence interval does not cross any thresholds however, the optimal information size of ≥400 participants is not met. (MID for waist circumference: ±2 cm)

5. downgraded by 1 level for RoB: less than 2/3 of the studies (and their contributing weight) were rated with a low RoB; 2/3 of the studies (but less than 2/3 of their contributing weight) were rated with a high RoB. Specified for the present comparison: 2/3 studies with high RoB (10.4% high RoB).

6. downgraded by 1 level due to imprecision. The effect is large and the 95%CI does not cross the threshold for MID however, the optimal information size of participants (OIS ≥ 400 per group) is not met. (MID for blood pressure: ±2 mmHg)

7. downgraded by 1 level for RoB: less than 2/3 of the studies (and their contributing weight) were rated with a low RoB; 2/3 of the studies (but less than 2/3 of their contributing weight) were rated with a high RoB. Specified for the present comparison: 2/3 studies with high RoB (58.7% high RoB).

# Supplemental Table 7. League table for the outcome LDL-cholesterol (mmol/L) comparing all plant-drinks and cow’s milk

| **Soy drink** | 0.06 [-0.90; 1.01] | NA | -0.51 [-0.90; -0.12] |
| --- | --- | --- | --- |
| -0.18 [-0.88; 0.52] | **Oat drink** | -0.16 [-1.12; 0.80] | -0.06 [-1.01; 0.90] |
| -0.34 [-1.53; 0.85] | -0.16 [-1.12; 0.80] | **Rice drink** | NA |
| -0.47 [-0.85; -0.10] | -0.29 [-1.00; 0.41] | -0.13 [-1.32; 1.05] | **Cow’s milk** |

Mean differences (MD) are computed from the main network meta-analysis (NMA) with harmonized doses (500 ml/d) using post- and change scores. The upper triangle describes the MD and 95% CI from direct comparison; the lower triangle displays the MD and 95% CI computed from the NMA. For the upper triangle, MDs < 0 favor the row-defining treatment. For the lower triangle, MDs < 0 favor the column-defining treatment.

The heterogeneity standard deviation (τ) was estimated at 0.48.

# Supplemental Table 8. GRADE assessment of direct, indirect and network estimates for the outcome LDL-cholesterol (mmol/L) comparing all plant-drinks and cow’s milk

|  |  | **Direct evidence** | | | **Indirect evidence** | | **Network Meta-Analysis** | |
| --- | --- | --- | --- | --- | --- | --- | --- | --- |
| **Comparison** | **N studies** | **Proportion direct evidence** | **MD (95%CI)** | **Certainty of evidence** | **MD (95%CI)** | **Certainty of evidence** | **MD (95%CI)** | **Certainty of evidence** |
| **Soy drink vs. oat drink** | 1 | 54 | 0.06 [-0.90; 1.01] | ⨁⨁◯◯^1^ | -0.46 [-1.49; 0.58] | ⨁⨁◯◯ | -0.18 [-0.88; 0.52] | ⨁◯◯◯^2^ |
| **Soy drink vs. rice drink** | NA | 0 | NA | NA | -0.34 [-1.53; 0.85] | ⨁⨁◯◯ | -0.34 [-1.53; 0.85] | ⨁◯◯◯^2^ |
| **Soy drink vs. cow’s milk** | 7 | 92 | -0.51 [-0.90; -0.12] | ⨁⨁⨁◯^3^ | 0.00 [-1.35; 1.35] | ⨁⨁◯◯ | -0.47 [-0.85; -0.10] | ⨁⨁◯◯^4^ |
| **Oat drink vs. rice drink** | 1 | 100 | -0.16 [-1.12; 0.80] | ⨁⨁◯◯^1^ | NA | NA | -0.16 [-1.12; 0.80] | ⨁◯◯◯^5^ |
| **Oat drink vs. cow’s milk** | 1 | 54 | -0.06 [-1.01; 0.90] | ⨁⨁◯◯^1^ | -0.57 [-1.60; 0.46] | ⨁⨁◯◯ | -0.29 [-1.00; 0.41] | ⨁◯◯◯^5^ |
| **Rice drink vs. cow’s milk** | 0 | 0 | NA | NA | -0.13 [-1.32; 1.05] | ⨁⨁◯◯ | -0.13 [-1.32; 1.05] | ⨁◯◯◯^5^ |

95%CI 95% confidence interval, MD mean difference, MID minimally important difference; NA not applicable, RoB risk of bias, ⨁⨁⨁⨁ high, ⨁⨁⨁◯ moderate; ⨁⨁◯◯ low, ⨁◯◯◯ very low

Explanations

^1^downgraded by two levels for RoB: More than 2/3 of the studies (and their contributing weight) were rated with a high RoB. No subgroup analysis for RoB could be conducted to test to robustness of the effect estimates. Specified for the present comparison: 1/1 study with high RoB.

^2^downgraded by two levels for imprecision: The point estimate suggests a small benefit, but the 95% CI includes the possibility of an important benefit, trivial effect, and an important harm. (MID for LDL-cholesterol: ±0.1 mmol/L)

^3^downgraded by one level for RoB: Less than 2/3 of the studies (and their contributing weight) were rated with a high RoB. The subgroup analysis excluding high RoB could be conducted to test the robustness of the effect estimates. Specified for the present comparison: 4/7 studies with high RoB (56%). The subgroup analysis shows a robust estimate (95% CIs overlap to a high degree).

^4^downgraded by one level for imprecision: The point estimate suggests a moderate benefit, but the 95% CI includes the possibility of trivial effect. (MID for LDL-cholesterol: ±0.1 mmol/L)

^5^downgraded by one level for imprecision: The point estimate suggests an important benefit, but the 95% CI includes the possibility of an important harm, which would be a reason to downgrade by two levels however, the certainty is already very low. (MID for LDL-cholesterol: ±0.1 mmol/L)

# Supplemental Table 9. League table for the outcome HDL-cholesterol (mmol/L) comparing all plant-drinks and cow’s milk

| **Soy drink** | 0.00 [-0.08; 0.08] | NA | 0.01 [-0.03; 0.05] |
| --- | --- | --- | --- |
| 0.01 [-0.05; 0.07] | **Oat drink** | -0.01 [-0.12; 0.09] | 0.00 [-0.08; 0.08] |
| -0.01 [-0.13; 0.11] | -0.01 [-0.12; 0.09] | **Rice drink** | NA |
| 0.01 [-0.03; 0.05] | 0.01 [-0.05; 0.07] | 0.02 [-0.10, 0.14] | **Cow’s milk** |

Mean differences (MD) are computed from the main network meta-analysis (NMA) with harmonized doses (500 ml/d) using post- and change scores. The upper triangle describes the MD and 95% CI from direct comparison; the lower triangle displays the MD and 95% CI computed from the NMA. For the upper triangle, MDs > 0 favor the row-defining treatment. For the lower triangle, MDs > 0 favor the column-defining treatment.

The heterogeneity standard deviation (τ) was estimated at 0.03.

# Supplemental Table 10. GRADE assessment of direct, indirect and network estimates for the outcome HDL-cholesterol (mmol/L) comparing all plant-drinks and cow’s milk

|  |  | **Direct evidence** | | | **Indirect evidence** | | **Network Meta-Analysis** | |
| --- | --- | --- | --- | --- | --- | --- | --- | --- |
| **Comparison** | **N studies** | **Proportion direct evidence** | **MD (95%CI)** | **Certainty of evidence** | **MD (95%CI)** | **Certainty of evidence** | **MD (95%CI)** | **Certainty of evidence** |
| **Soy drink vs. oat drink** | 1 | 58 | 0.00 [-0.08; 0.08] | ⨁⨁◯◯^1^ | 0.01 [-0.08; 0.11] | ⨁⨁◯◯ | 0.01 [-0.05; 0.07] | ⨁⨁◯◯ |
| **Soy drink vs. rice drink** | 0 | 0 | NA | NA | -0.01 [-0.13; 0.11] | ⨁⨁◯◯ | -0.01 [-0.13; 0.12] | ⨁◯◯◯^2^ |
| **Soy drink vs. cow’s milk** | 5 | 86 | 0.01 [-0.03; 0.05] | ⨁⨁⨁◯^3^ | 0.00 [-0.11, 0.11] | ⨁⨁◯◯ | 0.01 [-0.03; 0.05] | ⨁⨁⨁◯ |
| **Oat drink vs. rice drink** | 1 | 100 | -0.01 [-0.12; 0.09] | ⨁⨁◯◯^1^ | NA | NA | -0.01 [-0.12; 0.09] | ⨁◯◯◯^4^ |
| **Oat drink vs. cow’s milk** | 1 | 56 | 0.00 [-0.08; 0.08] | ⨁⨁◯◯^1^ | 0.01 [-0.08; 0.10] | ⨁⨁◯◯ | 0.01 [-0.05; 0.07] | ⨁⨁◯◯ |
| **Rice drink vs. cow’s milk** | 0 | 0 | NA | NA | 0.02 [-0.10; 0.14] | ⨁⨁◯◯ | 0.02 [-0.10, 0.14] | ⨁◯◯◯^2^ |

95%CI 95% confidence interval, MD mean difference, MID minimally important difference; NA not applicable, RoB risk of bias, ⨁⨁⨁⨁ high, ⨁⨁⨁◯ moderate; ⨁⨁◯◯ low, ⨁◯◯◯ very low

Explanations

^1^downgraded by two levels for RoB: More than 2/3 of the studies (and their contributing weight) were rated with a high RoB. No subgroup analysis for RoB could be conducted to test to robustness of the effect estimates. Specified for the present comparison: 1/1 study with high RoB.

^2^downgraded by one level for imprecision: The point estimate suggests a trivial effect, but the 95% CI includes the possibility of both important benefit and important harm. (MID for HDL-cholesterol: ±0.1 mmol/L)

^3^downgraded by one level for risk of bias: More than 2/3 of the studies (contributing weight 92.7%, 4/5 studies) are rated with high RoB. But the effect estimate remains robust in subgroup analysis when excluding studies with high risk of bias.

^4^downgraded by one level for imprecision: The point estimate suggests a trivial effect, but the 95% CI includes the possibility of an important benefit. (MID for HDL-cholesterol: ±0.1 mmol/L)

# Supplemental Table 11. League table for the outcome triglycerides (mmol/L) comparing all plant-drinks and cow’s milk

| **Soy drink** | 0.00 [-0.16; 0.16] | -0.17 [-0.52; 0.17] | -0.02 [-0.07; 0.04] |
| --- | --- | --- | --- |
| -0.02 [-0.12; 0.08] | **Oat drink** | -0.12 [-0.33; 0.09] | 0.00 [-0.12; 0.12] |
| -0.16 [-0.35; 0.02] | -0.15 [-0.32; 0.03] | **Rice drink** | 0.22 [-0.09; 0.53] |
| -0.02 [-0.07; 0.04] | 0.00 [-0.09; 0.09] | 0.15 [-0.04; 0.33] | **Cow’s milk** |

Mean differences (MD) are computed from the main network meta-analysis (NMA) with harmonized doses (500 ml/d) using post- and change scores. The upper triangle describes the MD and 95% CI from direct comparison; the lower triangle displays the MD and 95% CI computed from the NMA. For the upper triangle, MDs < 0 favor the row-defining treatment. For the lower triangle, MDs < 0 favor the column-defining treatment.

The heterogeneity standard deviation (τ) was estimated at 0.

# Supplemental Table 12. GRADE assessment of direct, indirect and network estimates for the outcome triglycerides (mmol/L) comparing all plant-drinks and cow’s milk

|  |  | **Direct evidence** | | | **Indirect evidence** | | **Network Meta-Analysis** | |
| --- | --- | --- | --- | --- | --- | --- | --- | --- |
| **Comparison** | **N studies** | **Proportion direct evidence** | **MD (95%CI)** | **Certainty of evidence** | **MD (95%CI)** | **Certainty of evidence** | **MD (95%CI)** | **Certainty of evidence** |
| **Soy drink vs. oat drink** | 1 | 38 | 0.00 [-0.16; 0.16] | ⨁⨁◯◯^1^ | -0.03 [-0.15; 0.10] | ⨁⨁◯◯ | -0.02 [-0.12; 0.08] | ⨁◯◯◯^2^ |
| **Soy drink vs. rice drink** | 1 | 28 | -0.17 [-0.52; 0.17] | ⨁⨁⨁◯^3^ | -0.16 [-0.38; 0.06] | ⨁⨁⨁◯ | -0.16 [-0.35; 0.02] | ⨁⨁◯◯^4^ |
| **Soy drink vs. cow’s milk** | 6 | 92 | -0.02 [-0.08; 0.04] | ⨁⨁⨁◯^5^ | 0.00 [-0.20; 0.20] | ⨁⨁◯◯ | -0.02 [-0.07; 0.04] | ⨁⨁⨁◯ |
| **Oat drink vs. rice drink** | 1 | 70 | -0.12 [-0.33; 0.09] | ⨁⨁◯◯^1^ | -0.21 [-0.52; 0.10] | ⨁⨁◯◯ | -0.15 [-0.32; 0.03] | ⨁◯◯◯^4^ |
| **Oat drink vs. cow’s milk** | 1 | 63 | 0.00 [-0.12; 0.12] | ⨁⨁◯◯^1^ | 0.00 [-0.16; 0.15] | ⨁⨁◯◯ | 0.00 [-0.09; 0.09] | ⨁⨁◯◯ |
| **Rice drink vs. cow’s milk** | 1 | 35 | 0.22 [-0.09; 0.53] | ⨁⨁⨁◯^3^ | 0.11 [-0.12; 0.33] | ⨁⨁⨁◯ | 0.15 [-0.04; 0.33] | ⨁⨁◯◯^6^ |

95%CI 95% confidence interval, MD mean difference, MID minimally important difference; NA not applicable, RoB risk of bias, ⨁⨁⨁⨁ high, ⨁⨁⨁◯ moderate; ⨁⨁◯◯ low, ⨁◯◯◯ very low

Explanations

^1^downgraded by two levels for RoB: More than 2/3 of the studies (and their contributing weight) were rated with a high RoB. No subgroup analysis for RoB could be conducted to test to robustness of the effect estimates. Specified for the present comparison: 1/1 study with high RoB.

^2^downgraded by one level for imprecision: The point estimate suggests a trivial effect, but the 95% CI includes the possibility of an important benefit. (MID for triglycerides: ±0.1 mmol/L)

^3^downgraded by one level for RoB: Less than 2/3 of the studies (and their contributing weight) were rated with a low RoB; Less than 2/3 of the studies (and less than 2/3 of their contributing weight) were rated with a high RoB. Specified for the present comparison: The included study was rated with “some concerns”.

^4^downgraded by one level for imprecision: The point estimate suggests an important benefit, but the 95% CI includes the possibility of a trivial effect. (MID for triglycerides: ±0.1 mmol/L)

^5^downgraded by 1 level for RoB: 2/3 of the studies (and more than 2/3 of their contributing weight) were rated with a high RoB. A subgroup analysis excluding high RoB could be conducted to test to robustness of the effect estimates. Specified for the present comparison: 4/6 studies with high RoB (92.4%). The subgroup analysis shows a robust estimate (95% CIs overlap to a high degree). ^6^downgraded by one level for imprecision: The point estimate suggests an important harm, but the 95% CI includes the possibility of a trivial effect. (MID for triglycerides: ±0.1 mmol/L)

# Supplemental Table 13. League table for the outcome total cholesterol (mmol/L) comparing all plant-drinks and cow’s milk

| **Soy drink** | 0.06 [-0.10; 0.22] | -0.33 [-0.92; 0.26] | -0.08 [-0.14; -0.02] |
| --- | --- | --- | --- |
| 0.04 [-0.07; 0.16] | **Oat drink** | -0.22 [-0.41; -0.03] | -0.11 [-0.27; 0.04] |
| -0.18 [-0.39; 0.02] | -0.23 [-0.40; -0.05] | **Rice drink** | 0.08 [-0.48; 0.63] |
| -0.08 [-0.14; -0.02] | -0.12 [-0.24; -0.01] | 0.10 [-0.10; 0.31] | **Cow’s milk** |

Mean differences (MD) are computed from the main network meta-analysis (NMA) with harmonized doses (500 ml/d) using post- and change scores. The upper triangle describes the MD and 95% CI from direct comparison; the lower triangle displays the MD and 95% CI computed from the NMA. For the upper triangle, MDs < 0 favor the row-defining treatment. For the lower triangle, MDs < 0 favor the column-defining treatment.

The heterogeneity standard deviation (τ) was estimated at 0.

# Supplemental Table 14. GRADE assessment of direct, indirect and network estimates for the outcome total cholesterol (mmol/L) comparing all plant-drinks and cow’s milk

|  |  | **Direct evidence** | | | **Indirect evidence** | | **Network Meta-Analysis** | |
| --- | --- | --- | --- | --- | --- | --- | --- | --- |
| **Comparison** | **N studies** | **Proportion direct evidence** | **MD (95%CI)** | **Certainty of evidence** | **MD (95%CI)** | **Certainty of evidence** | **MD (95%CI)** | **Certainty of evidence** |
| **Soy drink vs. oat drink** | 1 | 51 | 0.06 [-0.10; 0.22] | ⨁⨁◯◯^1^ | 0.03 [-0.13; 0.19] | ⨁⨁◯◯ | 0.04 [-0.07; 0.16] | ⨁◯◯◯^2^ |
| **Soy drink vs. rice drink** | 1 | 12 | -0.33 [-0.92; 0.26] | ⨁⨁◯◯^1^ | -0.16 [-0.38, 0.05] | ⨁⨁◯◯ | -0.18 [-0.39; 0.02] | ⨁◯◯◯^3^ |
| **Soy drink vs. cow’s milk** | 8 | 93 | -0.08 [-0.14; -0.02] | ⨁⨁◯◯^4^ | -0.06 [-0.28; 0.17] | ⨁⨁◯◯ | -0.08 [-0.14; -0.02] | ⨁◯◯◯^5^ |
| **Oat drink vs. rice drink** | 1 | 88 | -0.22 [-0.41; -0.03] | ⨁⨁◯◯^1^ | -0.28 [-0.80; 0.24] | ⨁⨁◯◯ | -0.23 [-0.40; -0.05] | ⨁◯◯◯^3^ |
| **Oat drink vs. cow’s milk** | 1 | 51 | -0.11 [-0.27; 0.04] | ⨁⨁◯◯^1^ | -0.13 [-0.30; 0.03] | ⨁⨁◯◯ | -0.12 [-0.24; -0.01] | ⨁◯◯◯^3^ |
| **Rice drink vs. cow’s milk** | 1 | 13 | 0.08 [-0.48; 0.63] | ⨁⨁⨁◯^6^ | 0.11 [-0.11; 0.33] | ⨁⨁◯◯ | 0.10 [-0.10; 0.31] | ⨁◯◯◯^7^ |

95%CI 95% confidence interval, MD mean difference, MID minimally important difference; NA not applicable, RoB risk of bias, ⨁⨁⨁⨁ high, ⨁⨁⨁◯ moderate; ⨁⨁◯◯ low, ⨁◯◯◯ very low

Explanations

^1^downgraded by two levels for RoB: More than 2/3 of the studies (and their contributing weight) were rated with a high RoB. No subgroup analysis for RoB could be conducted to test to robustness of the effect estimates. Specified for the present comparison: 1/1 study with high RoB.

^2^downgraded by one level for imprecision: The point estimate suggests a trivial effect, but the 95% CI includes the possibility of both important benefit and important harm. (MID for total cholesterol: ±0.1 mmol/L)

^3^downgraded by one level for imprecision: The point estimate suggests an important benefit effect, but the 95% CI includes the possibility of a trivial effect. (MID for total cholesterol: ±0.1 mmol/L)

^4^downgraded by two levels for risk of bias: More than 2/3 of the studies (contributing weight 78.5%, but 4/8 studies) are rated with high RoB. The estimates of subgroup analysis by RoB-rating suggest differing effect estimates (larger benefit in some concerns studies, CI do not overlap).

^5^downgraded by one level for imprecision: The point estimate suggests a trivial effect, but the 95% CI includes the possibility of an important benefit. (MID for total cholesterol: ±0.1 mmol/L)

^6^downgraded by one level for RoB: Less than 2/3 of the studies (and their contributing weight) were rated with a low RoB; Less than 2/3 of the studies (and less than 2/3 of their contributing weight) were rated with a high RoB. Specified for the present comparison: The included study was rated with “some concerns”.

^7^downgraded by two levels for imprecision: The point estimate suggests an important harm, but the 95% CI includes the possibility of an important benefit. (MID for total cholesterol: ±0.1 mmol/L)

# Supplemental Table 15. League table for the outcome fasting blood glucose (mmol/L) comparing all plant-drinks and cow’s milk

| **Soy drink** | 0.06 [-1.99; 2.11] | NA | -1.06 [-2.53; 0.41] |
| --- | --- | --- | --- |
| -0.39 [-1.98; 1.20] | **Oat drink** | -0.07 [-2.12; 1.98] | 0.00 [-2.05; 2.05] |
| -0.45 [-3.05; 2.14] | -0.07 [-2.12; 1.98] | **Rice drink** | NA |
| -0.83 [-2.14; 0.48] | -0.44 [-2.04; 1.15] | -0.38 [-2.97; 2.22] | **Cow’s milk** |

Mean differences (MD) are computed from the main network meta-analysis (NMA) with harmonized doses (500 ml/d) using post- and change scores. The upper triangle describes the MD and 95% CI from direct comparison; the lower triangle displays the MD and 95% CI computed from the NMA. For the upper triangle, MDs < 0 favor the row-defining treatment. For the lower triangle, MDs < 0 favor the column-defining treatment.

The heterogeneity standard deviation (τ) was estimated at 1.04.

# Supplemental Table 16. GRADE assessment of direct, indirect and network estimates for the outcome fasting blood glucose (mmol/L) comparing all plant-drinks and cow’s milk

|  |  | **Direct evidence** | | | **Indirect evidence** | | **Network Meta-Analysis** | |
| --- | --- | --- | --- | --- | --- | --- | --- | --- |
| **Comparison** | **N studies** | **Proportion direct evidence** | **MD (95%CI)** | **Certainty of evidence** | **MD (95%CI)** | **Certainty of evidence** | **MD (95%CI)** | **Certainty of evidence** |
| **Soy drink vs. oat drink** | 1 | 60 | 0.06 [-1.99; 2.11] | ⨁⨁◯◯^1^ | -1.06 [-3.58; 1.46] | ⨁⨁◯◯ | -0.39 [-1.98; 1.20] | ⨁◯◯◯^2^ |
| **Soy drink vs. rice drink** | 0 | 0 | NA | NA | -0.45 [-3.05; 2.14] | ⨁⨁◯◯ | -0.45 [-3.05; 2.14] | ⨁◯◯◯^2^ |
| **Soy drink vs. cow’s milk** | 2 | 80 | -1.06 [-2.53; 0.41] | ⨁⨁◯◯^3^ | 0.06 [-2.84; 2.96] | ⨁⨁◯◯ | -0.83 [-2.14; 0.48] | ⨁◯◯◯^4^ |
| **Oat drink vs. rice drink** | 1 | 100 | -0.07 [-2.12; 1.98] | ⨁⨁◯◯^1^ | NA | NA | -0.07 [-2.12; 1.98] | ⨁◯◯◯^2^ |
| **Oat drink vs. cow’s milk** | 1 | 60 | 0.00 [-2.05; 2.05] | ⨁⨁◯◯^1^ | -1.12 [-3.64; 1.40] | ⨁⨁◯◯ | -0.44 [-2.04; 1.15] | ⨁◯◯◯^2^ |
| **Rice drink vs. cow’s milk** | 0 | 0 | NA | NA | -0.38 [-2.97; 2.22] | ⨁⨁◯◯ | -0.38 [-2.97; 2.22] | ⨁◯◯◯^2^ |

95%CI 95% confidence interval, MD mean difference, MID minimally important difference; NA not applicable, RoB risk of bias, ⨁⨁⨁⨁ high, ⨁⨁⨁◯ moderate; ⨁⨁◯◯ low, ⨁◯◯◯ very low

Explanations

^1^downgraded by two levels for RoB: More than 2/3 of the studies (and their contributing weight) were rated with a high RoB. No subgroup analysis for RoB could be conducted to test to robustness of the effect estimates. Specified for the present comparison: 1/1 study with high RoB.

^2^downgraded by one level for imprecision: The point estimate suggests a trivial effect, but the 95% CI includes the possibility of both important benefit and important harm. (MID for fasting blood glucose: ±0.5 mmol/L)

^3^downgraded by two levels for RoB: More than 2/3 of the studies (and their contributing weight) were rated with a high RoB. No subgroup analysis for RoB could be conducted to test to robustness of the effect estimates. Specified for the present comparison: 2/2 study with high RoB.

^4^downgraded by one level for imprecision: The point estimate suggests an important benefit, but the 95% CI includes the possibility of a trivial effect. (MID for fasting blood glucose: ±0.5 mmol/L)

# Supplemental Table 17. Relative ranking of plant-drinks and cow milk (P-scores*)

| Outcome  Intervention | Body weight↓ | LDL-cholesterol↓ | HDL-cholesterol↑ | Triglycerides↓ | Total cholesterol↓ | Fasting blood glucose↓ |
| --- | --- | --- | --- | --- | --- | --- |
| Soy drink | 0.34 | **0.80** | 0.57 | **0.76** | 0.73 | **0.74** |
| Oat drink | 0.45 | 0.58 | 0.47 | 0.61 | **0.92** | 0.52 |
| Rice drink | 0.51 | 0.42 | **0.59** | 0.05 | 0.07 | 0.48 |
| Cow’s milk | **0.70** | 0.21 | 0.37 | 0.58 | 0.29 | 0.26 |

*P-scores were calculated and presented to obtain relative ranking of interventions. Higher P-score values indicate a greater benefit with a certain intervention. Bold are interventions identified as the best for the given outcome. (↓) = decrease is the effect of interest; (↑) = increase is the effect of interest

# Supplemental Table 18. Heterogeneity measures design-by-treatment statistics for NMA comparing different plant-drinks and cow’s milk

| **Outcome** | **Q** | **df** | **p-value** |
| --- | --- | --- | --- |
| **Body weight** | | | |
| Total | 1.20 | 3 | 0.75 |
| Within Designs | 0.50 | 2 | 0.78 |
| Between Designs | 0.71 | 1 | 0.40 |
| **LDL-Cholesterol** | | | |
| Total | 193.66 | 7 | <0.001 |
| Within Designs | 187.78 | 6 | <0.001 |
| Between Designs | 5.88 | 1 | 0.02 |
| **HDL-Cholesterol** | | | |
| Total | 10.12 | 5 | 0.07 |
| Within Designs | 9.73 | 4 | 0.05 |
| Between Designs | 0.39 | 1 | 0.53 |
| **Triglycerides** | | | |
| Total | 3.17 | 7 | 0.87 |
| Within Designs | 2.70 | 4 | 0.61 |
| Between Designs | 0.47 | 3 | 0.93 |
| **Total Cholesterol** | | | |
| Total | 8.84 | 9 | 0.45 |
| Within Designs | 8.35 | 6 | 0.21 |
| Between Designs | 0.49 | 3 | 0.92 |
| **Fasting Blood Glucose** | | | |
| Total | 161.75 | 2 | <0.001 |
| Within Designs | 40.84 | 1 | <0.001 |
| Between Designs | 120.91 | 1 | <0.001 |

HDL high-density lipoprotein; LDL low-density lipoprotein; NMA network meta-analysis, Q Cochran Q statistic, df degrees of freedom

# Supplemental Table 19. League table for the sensitivity analysis with post values only

**Harmonized NMA, outcome: triglycerides (mmol/L), comparing all plant-drinks and cow’s milk:**

| **Soy drink** | NA | -0.17 [-0.52; 0.17] | -0.01 [-0.07; 0.05] |
| --- | --- | --- | --- |
| -0.10 [-0.46; 0.27] | **Oat drink** | -0.12 [-0.33; 0.09] | NA |
| -0.22 [-0.52; 0.08] | -0.12 [-0.33; 0.09] | **Rice drink** | 0.22 [-0.09; 0.53] |
| -0.01 [-0.07; 0.05] | 0.08 [-0.28; 0.45] | 0.20 [-0.09; 0.50] | **Cow’s milk** |

Mean differences (MD) are computed from network meta-analysis (NMA) with harmonized doses (500 ml/d) using post values only. The upper triangle describes the MD and 95% CI from direct comparison; the lower triangle displays the MD and 95% CI computed from the NMA. For the upper triangle, MDs < 0 favor the row-defining treatment. For the lower triangle, MDs < 0 favor the column-defining treatment.

The heterogeneity standard deviation ($\hat{\tau}$) was estimated at 0.

# Supplemental Table 20. League table for the sensitivity analysis with post values only

**Harmonized NMA, outcome: total cholesterol (mmol/L), comparing all plant-drinks and cow’s milk:**

| **Soy drink** | NA | -0.33 [-0.92; 0.26] | -0.08 [-0.15; -0.01] |
| --- | --- | --- | --- |
| -0.01 [-0.55; 0.53] | **Oat drink** | -0.22 [-0.41; -0.03] | NA |
| -0.23 [-0.74; 0.28] | -0.22 [-0.41; -0.03] | **Rice drink** | 0.08 [-0.48; 0.63] |
| -0.08 [-0.15; -0.01] | -0.07 [-0.61; 0.47] | 0.15 [-0.36; 0.66] | **Cow’s milk** |

Mean differences (MD) are computed from network meta-analysis (NMA) with harmonized doses (500 ml/d) using post values only. The upper triangle describes the MD and 95% CI from direct comparison; the lower triangle displays the MD and 95% CI computed from the NMA. For the upper triangle, MDs < 0 favor the row-defining treatment. For the lower triangle, MDs < 0 favor the column-defining treatment.

The heterogeneity standard deviation ($\hat{\tau}$) was estimated at 0.

# Supplemental Table 21. League table for the sensitivity analysis with change scores only

**Harmonized NMA, outcome: body weight (kg), comparing all plant-drinks and cow’s milk:**

| **Soy drink** | -0.06 [-2.14; 2.02] | NA | -0.77 [-2.20; 0.66] |
| --- | --- | --- | --- |
| -0.37 [-1.97; 1.24] | **Oat drink** | 0.00 [-2.00; 2.00] | 0.06 [-2.02; 2.14] |
| -0.37 [-2.94; 2.20] | 0.00 [-2.00; 2.00] | **Rice drink** | NA |
| -0.62 [-1.91; 0.67] | -0.25 [-1.86; 1.35] | -0.25 [-2.82; 2.31] | **Cow’s milk** |

Mean differences (MD) are computed from network meta-analysis (NMA) with harmonized doses (500 ml/d) using change scores only. The upper triangle describes the MD and 95% CI from direct comparison; the lower triangle displays the MD and 95% CI computed from the NMA. For the upper triangle, MDs < 0 favor the row-defining treatment. For the lower triangle, MDs < 0 favor the column-defining treatment.

The heterogeneity standard deviation ($\hat{\tau}$) was estimated at 1.01.

# Supplemental Table 22. League table for the sensitivity analysis with change scores only

**Harmonized NMA, outcome: LDL-cholesterol (mmol/L), comparing all plant-drinks and cow’s milk:**

| **Soy drink** | 0.06 [-0.25; 0.37] | NA | -0.20 [-0.31; -0.09] |
| --- | --- | --- | --- |
| -0.04 [-0.26; 0.19] | **Oat drink** | -0.17 [-0.47; 0.13] | -0.06 [-0.37; 0.25] |
| -0.21 [-0.58; 0.17] | -0.17 [-0.47; 0.13] | **Rice drink** | NA |
| -0.19 [-0.30; -0.08] | -0.15 [-0.38; 0.07] | 0.01 [-0.36; 0.39] | **Cow’s milk** |

Mean differences (MD) are computed from network meta-analysis (NMA) with harmonized doses (500 ml/d) using change scores only. The upper triangle describes the MD and 95% CI from direct comparison; the lower triangle displays the MD and 95% CI computed from the NMA. For the upper triangle, MDs < 0 favor the row-defining treatment. For the lower triangle, MDs < 0 favor the column-defining treatment.

The heterogeneity standard deviation ($\hat{\tau}$) was estimated at 0.14.

# Supplemental Table 23. League table for the sensitivity analysis with change scores only

**Harmonized NMA, outcome: HDL-cholesterol (mmol/L), comparing all plant-drinks and cow’s milk:**

| **Soy drink** | 0.00 [-0.11; 0.11] | NA | 0.02 [-0.03; 0.07] |
| --- | --- | --- | --- |
| 0.01 [-0.07; 0.09] | **Oat drink** | -0.08 [-0.21; 0.05] | 0.00 [-0.11; 0.11] |
| -0.07 [-0.23; 0.08] | -0.08 [-0.21; 0.05] | **Rice drink** | NA |
| 0.02 [-0.03; 0.06] | 0.01 [-0.07; 0.09] | 0.09 [-0.07; 0.25] | **Cow’s milk** |

Mean differences (MD) are computed from network meta-analysis (NMA) with harmonized doses (500 ml/d) using change scores only. The upper triangle describes the MD and 95% CI from direct comparison; the lower triangle displays the MD and 95% CI computed from the NMA. For the upper triangle, MDs > 0 favor the row-defining treatment. For the lower triangle, MDs > 0 favor the column-defining treatment.

The heterogeneity standard deviation ($\hat{\tau}$) was estimated at 0.05.

# Supplemental Table 24. League table for the sensitivity analysis with change scores only

**Harmonized NMA, outcome: triglycerides (mmol/L), comparing all plant-drinks and cow’s milk:**

| **Soy drink** | 0.00 [-0.22; 0.22] | 0.08 [-0.13; 0.28] | -0.03 [-0.11; 0.05] |
| --- | --- | --- | --- |
| 0.01 [-0.13; 0.14] | **Oat drink** | -0.13 [-0.34; 0.09] | 0.00 [-0.19; 0.19] |
| -0.06 [-0.22; 0.09] | -0.07 [-0.23; 0.09] | **Rice drink** | 0.10 [-0.11; 0.31] |
| -0.03 [-0.10; 0.05] | -0.04 [-0.17; 0.10] | 0.04 [-0.12; 0.19] | **Cow’s milk** |

Mean differences (MD) are computed from network meta-analysis (NMA) with harmonized doses (500 ml/d) using change scores only. The upper triangle describes the MD and 95% CI from direct comparison; the lower triangle displays the MD and 95% CI computed from the NMA. For the upper triangle, MDs < 0 favor the row-defining treatment. For the lower triangle, MDs < 0 favor the column-defining treatment.

The heterogeneity standard deviation ($\hat{\tau}$) was estimated at 0.08.

# Supplemental Table 25. League table for the sensitivity analysis with change scores only

**Harmonized NMA, outcome: total cholesterol (mmol/L), comparing all plant-drinks and cow’s milk:**

| **Soy drink** | 0.06 [-0.23; 0.35] | -0.04 [-0.38; 0.29] | -0.19 [-0.29; -0.08] |
| --- | --- | --- | --- |
| 0.04 [-0.15; 0.23] | **Oat drink** | -0.24 [-0.52; 0.04] | -0.11 [-0.40; 0.17] |
| -0.11 [-0.35; 0.12] | -0.15 [-0.38; 0.07] | **Rice drink** | -0.22 [-0.60; 0.16] |
| -0.18 [-0.28; -0.07] | -0.22 [-0.41; -0.03] | -0.06 [-0.30; 0.17] | **Cow’s milk** |

Mean differences (MD) are computed from network meta-analysis (NMA) with harmonized doses (500 ml/d) using change scores only. The upper triangle describes the MD and 95% CI from direct comparison; the lower triangle displays the MD and 95% CI computed from the NMA. For the upper triangle, MDs < 0 favor the row-defining treatment. For the lower triangle, MDs < 0 favor the column-defining treatment.

The heterogeneity standard deviation ($\hat{\tau}$) was estimated at 0.12.

# Supplemental Table 26. League table for the sensitivity analysis with change scores only

**Harmonized NMA, outcome: fasting blood glucose (mmol/L), comparing all plant-drinks and cow’s milk:**

| **Soy drink** | 0.06 [-0.17; 0.29] | NA | -0.11 [-0.28; 0.05] |
| --- | --- | --- | --- |
| -0.01 [-0.19; 0.17] | **Oat drink** | -0.07 [-0.28; 0.15] | 0.00 [-0.23; 0.23] |
| -0.08 [-0.36; 0.20] | -0.07 [-0.28; 0.15] | **Rice drink** | NA |
| -0.08 [-0.23; 0.07] | -0.07 [-0.25; 0.11] | 0.00 [-0.28; 0.28] | **Cow’s milk** |

Mean differences (MD) are computed from network meta-analysis (NMA) with harmonized doses (500 ml/d) using change scores only. The upper triangle describes the MD and 95% CI from direct comparison; the lower triangle displays the MD and 95% CI computed from the NMA. For the upper triangle, MDs < 0 favor the row-defining treatment. For the lower triangle, MDs < 0 favor the column-defining treatment.

The heterogeneity standard deviation ($\hat{\tau}$) was estimated at 0.09.

# Supplemental Table 27. Selected dose-response models for dose-response network meta-analyses

| **Outcome** | **Selected dose-response model** |
| --- | --- |
| Body weight | Post and change scores: *Exponential* |
|  | Change scores (sensitivity analysis): *FP1 (p = 0)* |
| LDL-cholesterol | Post and change scores: *FP1 (p = 0)* |
|  | Change scores (sensitivity analysis): *FP1 (p = 0)* |
| HDL-cholesterol | Post and change scores: *Quadratic* |
|  | Change scores (sensitivity analysis): *RCS (0.25, 0.5, 1)* |
| Total cholesterol | Post and change scores: *FP1 (p = -0.5)* |
|  | Change scores (sensitivity analysis): *FP1 (p = -0.5)* |
| Triglycerides | Post and change scores: *FP1 (p = 2)* |
|  | Change scores (sensitivity analysis): *Exponential* |
| Systolic blood pressure | Post and change scores: *RCS (0.1, 0.5, 0.9)* |
|  | Change scores (sensitivity analysis): *Linear* |
| Diastolic blood pressure | Post and change scores: *FP1 (p = 0.5)* |
|  | Change scores (sensitivity analysis): *FP1 (p = 2)* |
| Fasting Glucose | Post and change scores: *FP1 (p = 2)* |
|  | Change scores (sensitivity analysis): *FP1 (p = -2)* |

HDL high-density lipoprotein; LDL-low-density lipoprotein

#
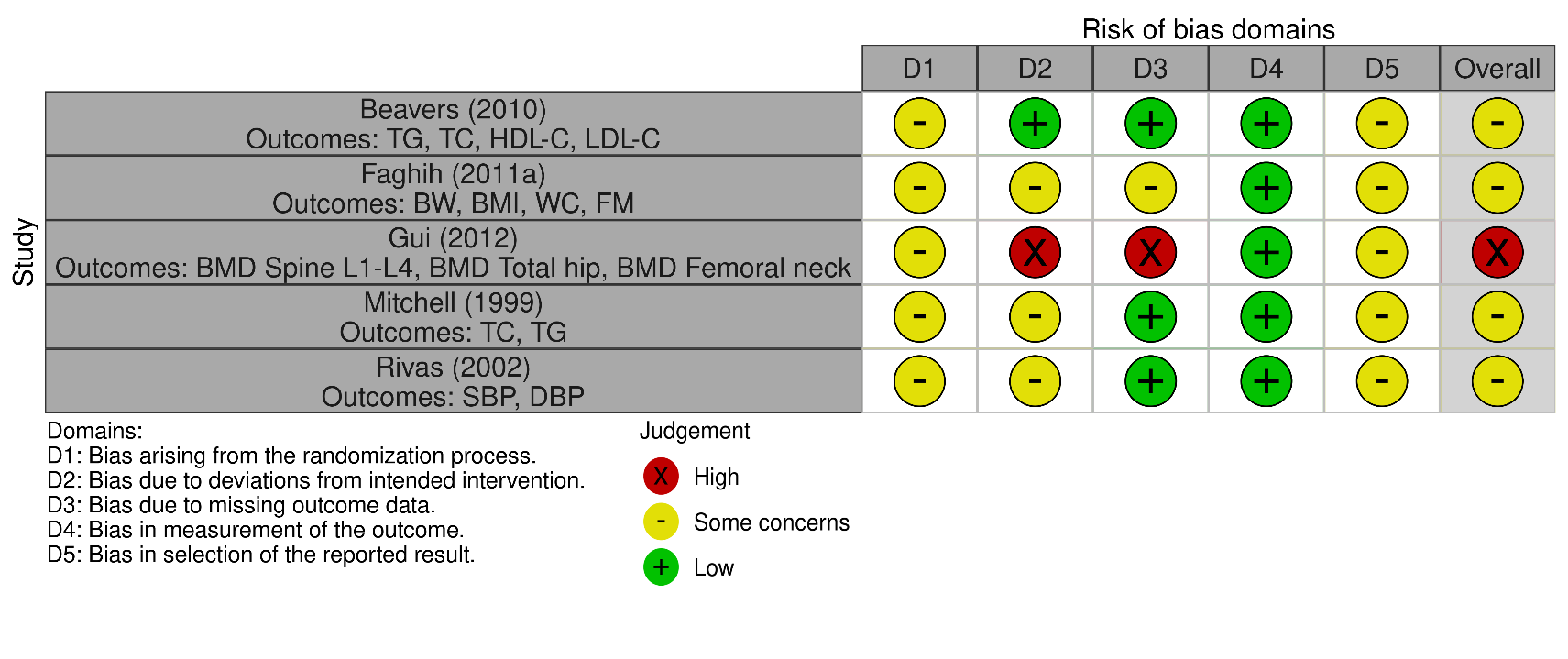
Supplemental Figure 1. Risk of Bias assessment for parallel trial

BMD bone density mass, BMI body mass index, BW Body weight, DBP diastolic blood pressure, FM fat mass, HDL-C high-density lipoprotein cholesterol, LDL-C low-density lipoprotein cholesterol, SBP systolic blood pressure, TC total cholesterol, TG triglycerides, WC waist circumference

#
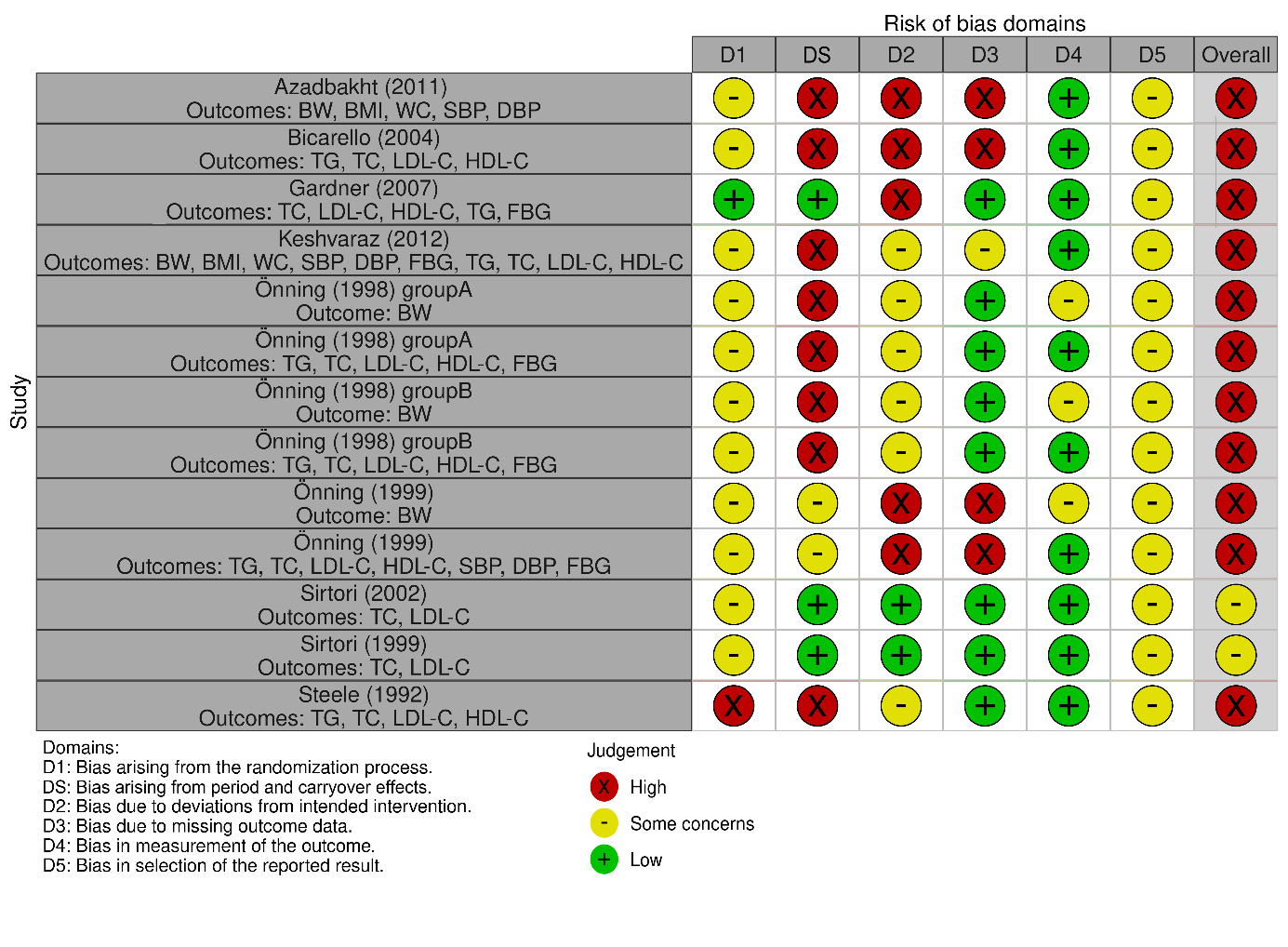
Supplemental Figure 2. Risk of Bias assessment for cross-over trials

BMI body mass index, BW Body weight, DBP diastolic blood pressure, FBG fasting blood glucose, FM fat mass, HDL-C high-density lipoprotein cholesterol, LDL-C low-density lipoprotein cholesterol, SBP systolic blood pressure, TC total cholesterol, TG triglycerides, WC waist circumference

Önning (1998) had a mixed design of parallel and cross-over trial, we assessed the risk of bias separately for group A and B (as cross-over trials), as this represented the way the study was reported and analysed.

# Supplemental Figure 3. Forest Plot summarizing mean differences with 95%CIs

**Harmonized NMA, outcome: body weight (kg), comparing all plant-drinks and cow’s milk**
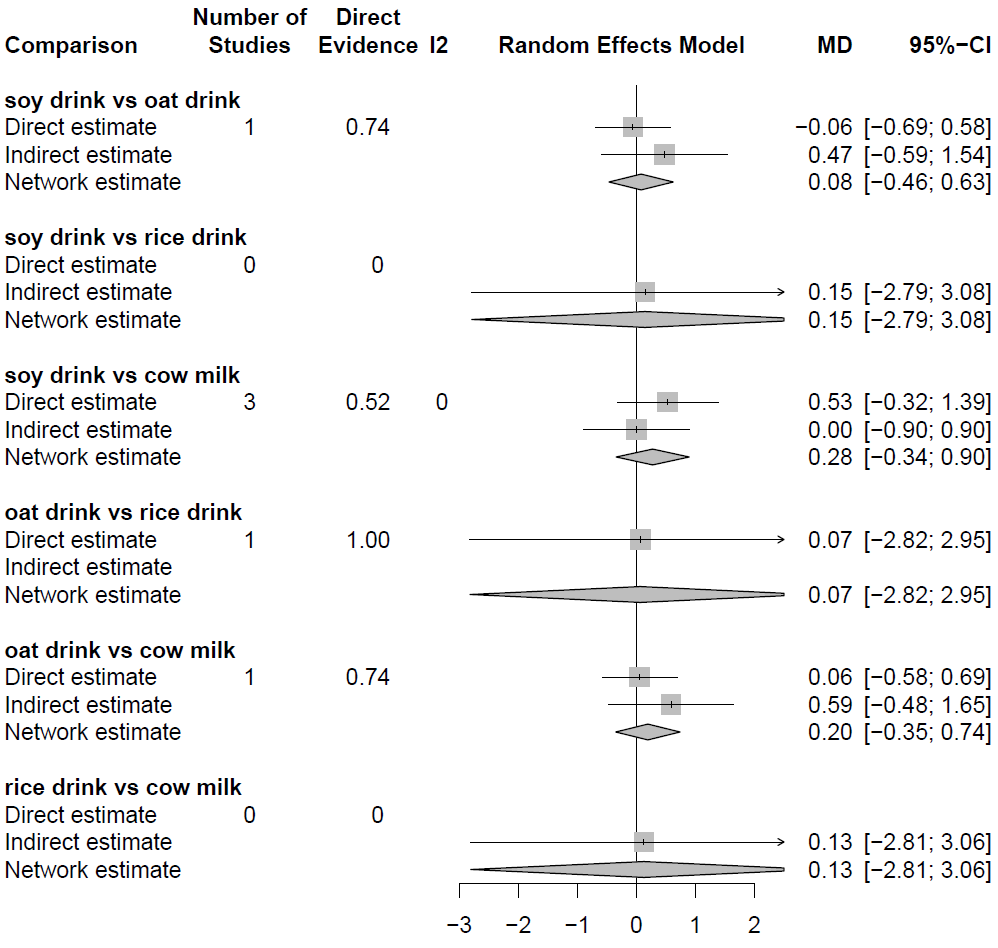


95%-CI 95% confidence interval, MD mean difference

# Supplemental Figure 4. Forest Plot summarizing mean differences with 95%CIs

**Pairwise meta-analysis, outcome: BMI (kg/m²), comparing soy drink and cow’s milk**


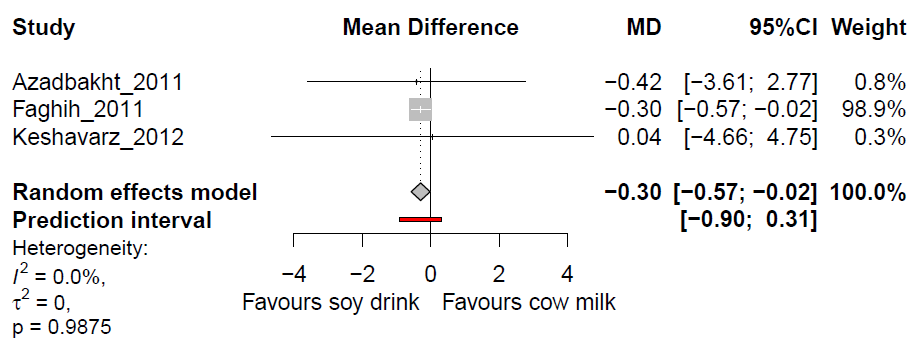


95%CI 95% confidence interval, BMI Body Mass Index, MD mean difference

# Supplemental Figure 5. Forest Plot summarizing mean differences with 95%CIs

**Pairwise meta-analysis, outcome: waist circumference (cm), comparing soy drink and cow’s milk**


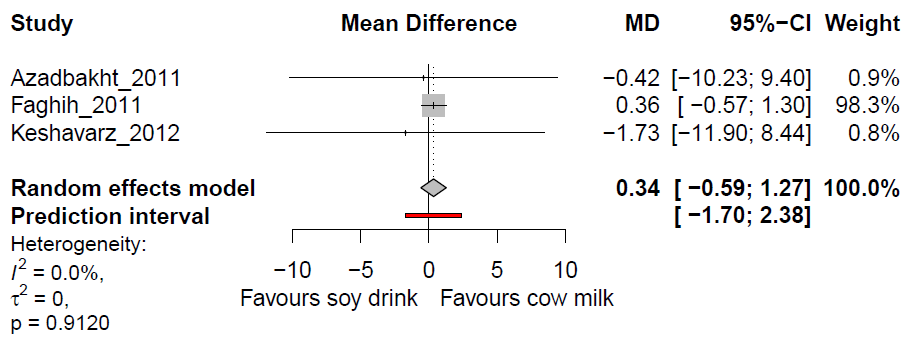


95%CI 95% confidence interval, MD mean difference

# Supplemental Figure 6. Forest Plot summarizing mean differences with 95%CIs

**Harmonized NMA, outcome: LDL-cholesterol (mmol/L), comparing all plant-drinks and cow’s milk**


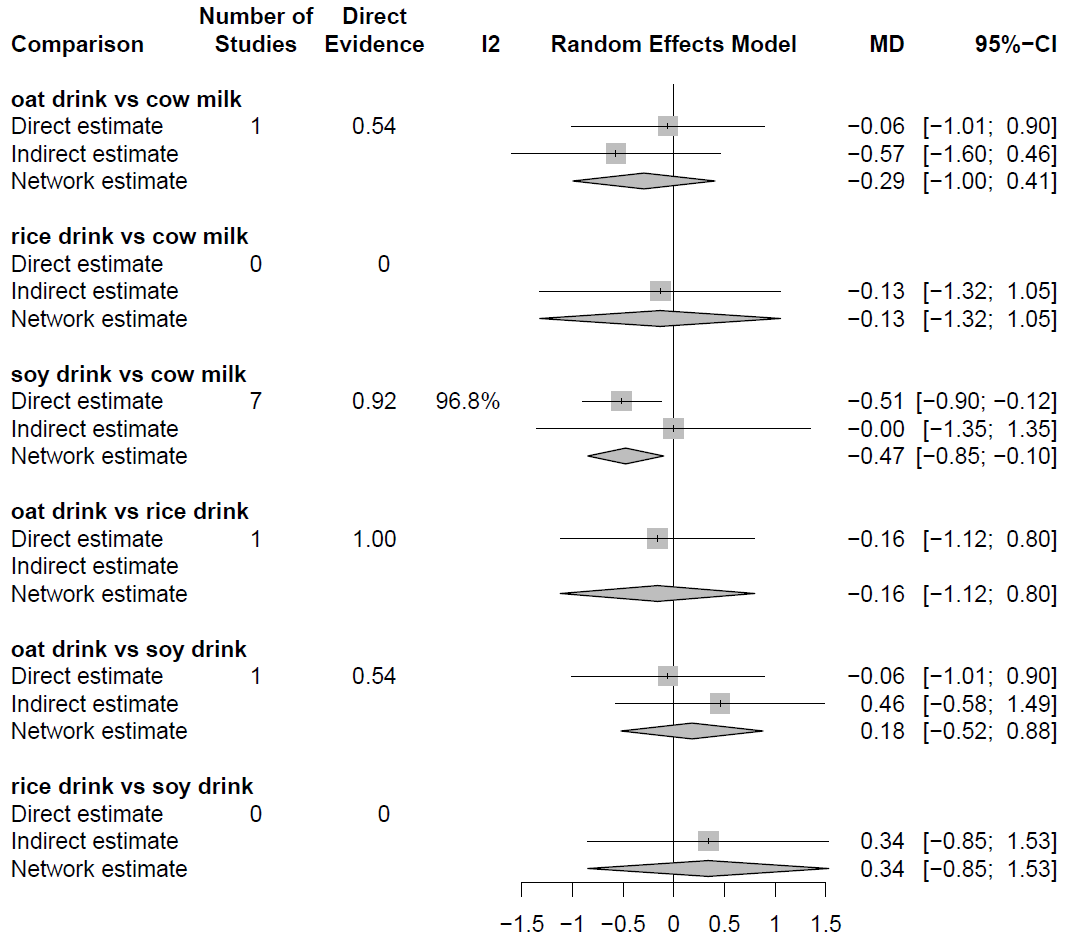


95%CI 95% confidence interval, LDL low-density lipoprotein, MD mean difference

# Supplemental Figure 7. Forest Plot summarizing mean differences with 95%CIs

**Harmonized NMA, outcome: HDL-cholesterol (mmol/L), comparing all plant-drinks and cow’s milk**


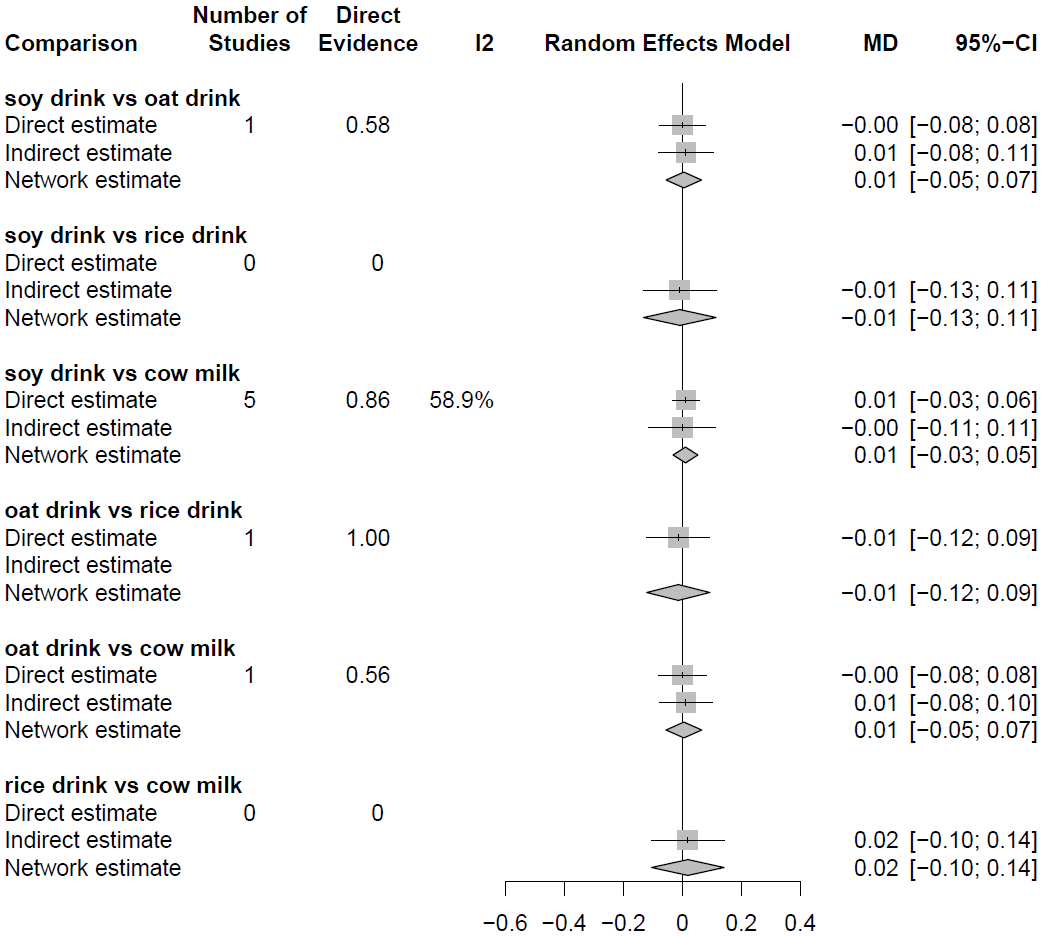


95%CI 95% confidence interval, HDL high-density lipoprotein, MD mean difference

# Supplemental Figure 8. Forest Plot summarizing mean differences with 95%CIs

**Harmonized NMA, outcome: triglycerides (mmol/L), comparing all plant-drinks and cow’s milk**


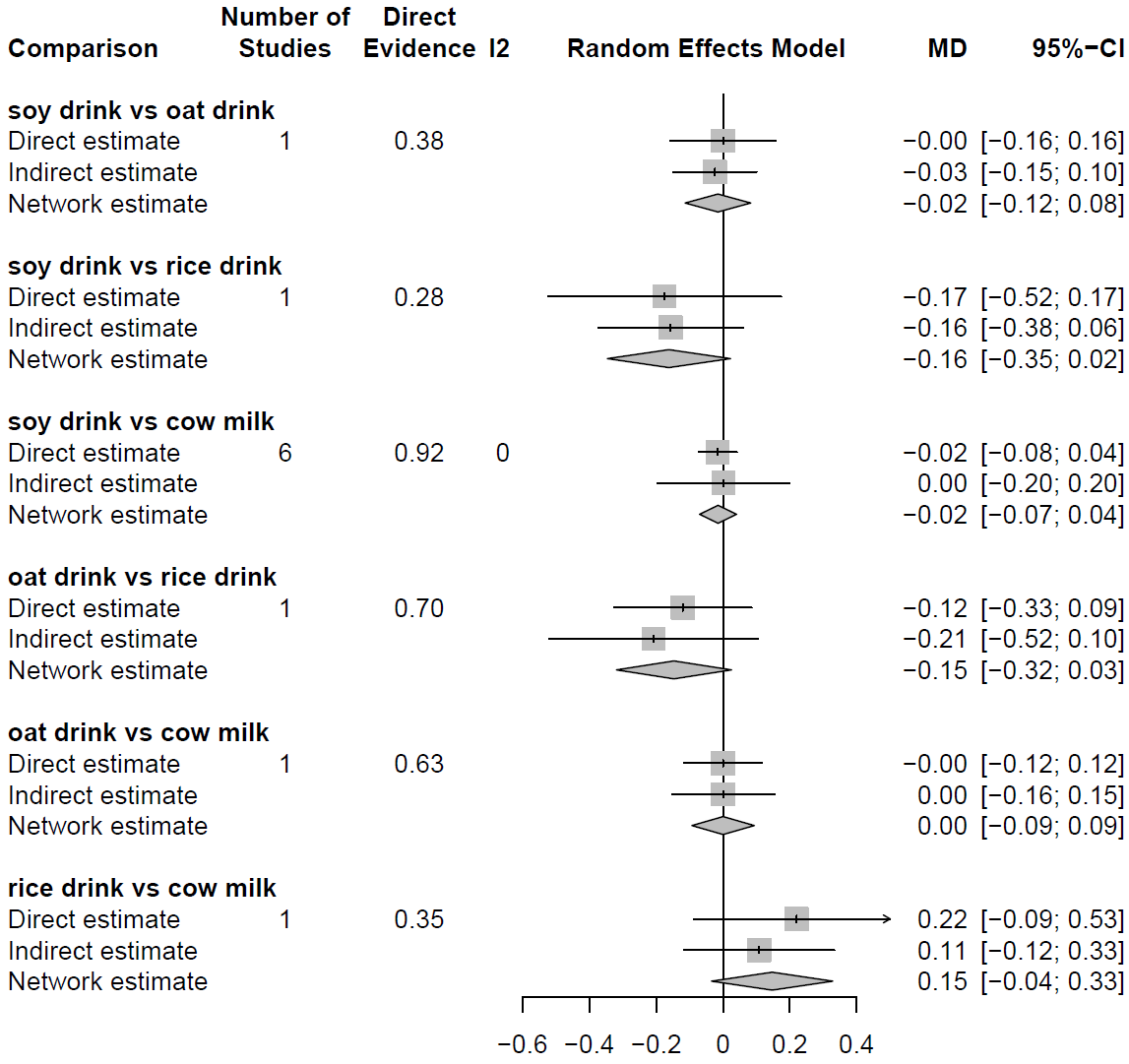


95%CI 95% confidence interval, MD mean difference

# Supplemental Figure 9. Forest Plot summarizing mean differences with 95%CIs

**Harmonized NMA, outcome: total-cholesterol (mmol/L), comparing all plant-drinks and cow’s milk**


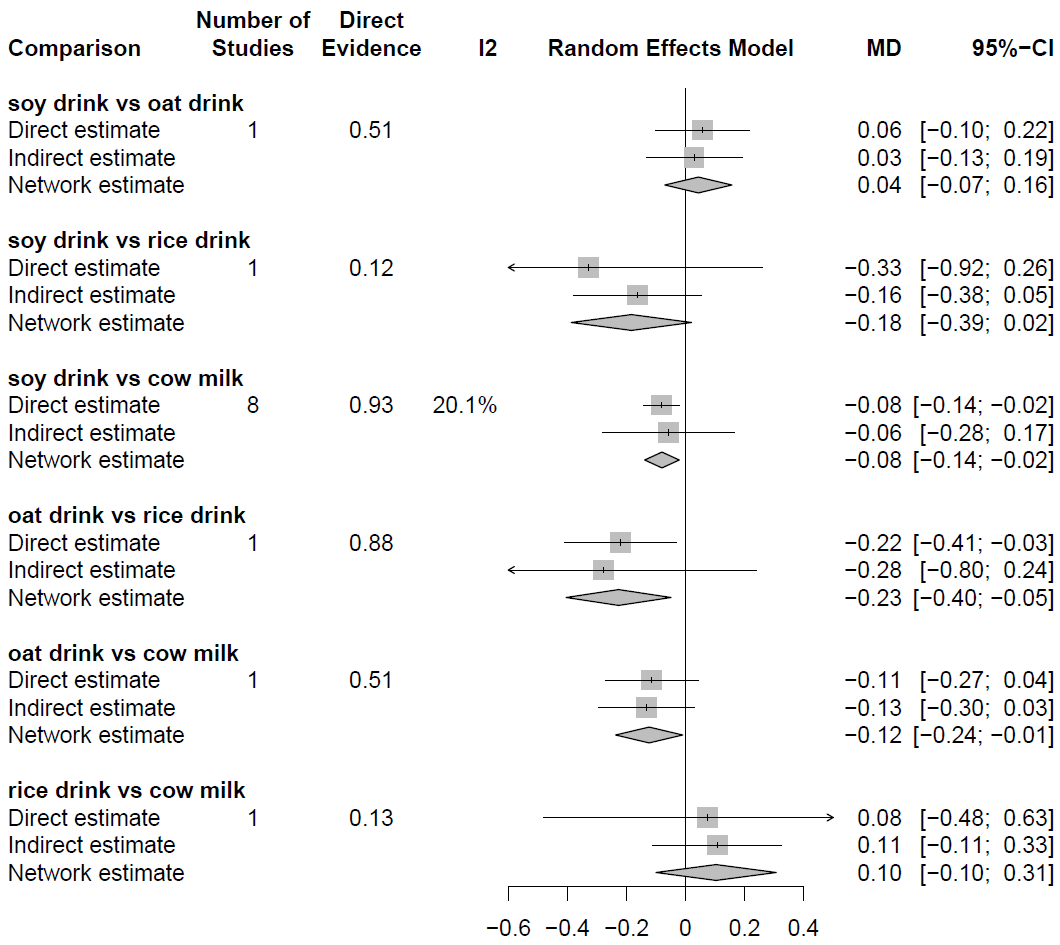


95%CI 95% confidence interval, MD mean difference

# Supplemental Figure 10. Forest Plot summarizing mean differences with 95%CIs

**Harmonized NMA, outcome: fasting blood glucose (mmol/L), comparing all plant-drinks and cow’s milk**


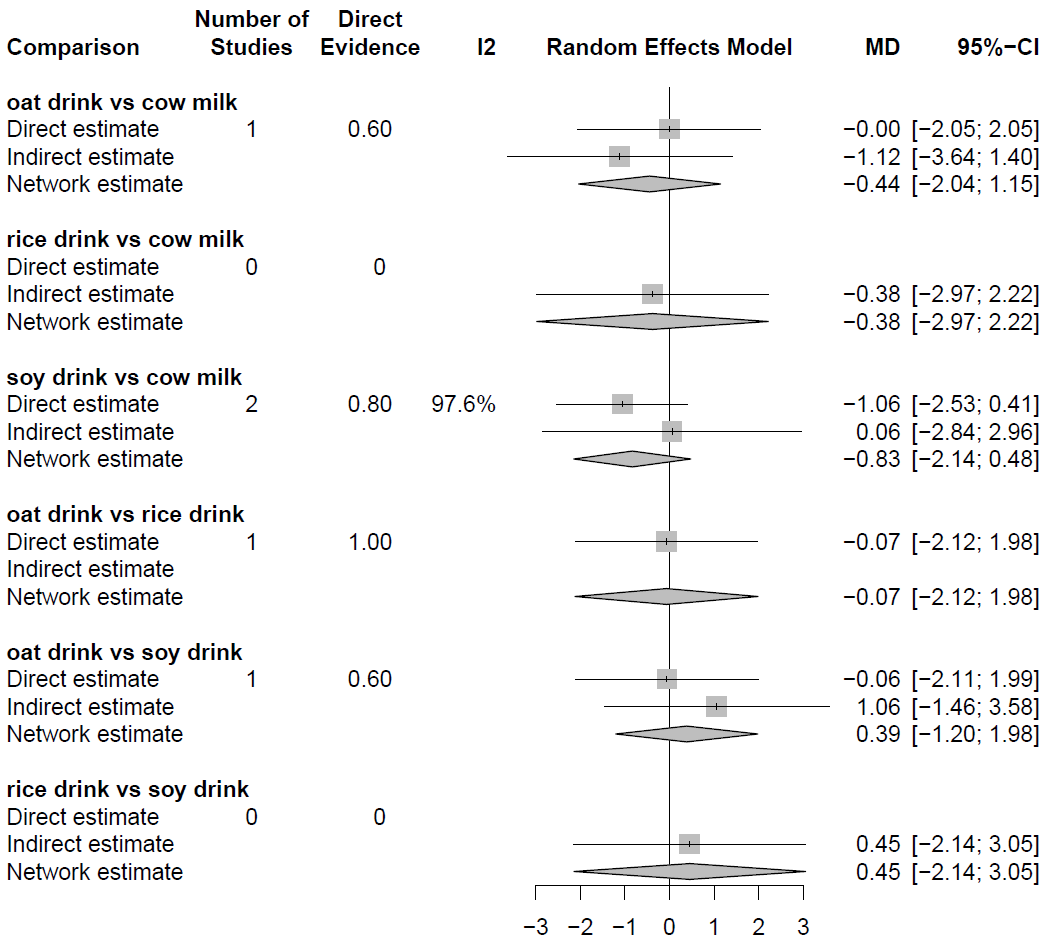


95%CI 95% confidence interval, MD mean difference

# Supplemental Figure 11. Forest Plot summarizing mean differences with 95%CIs

**Pairwise meta-analysis, outcome: systolic blood pressure (mmHg), comparing soy drink and cow’s milk**


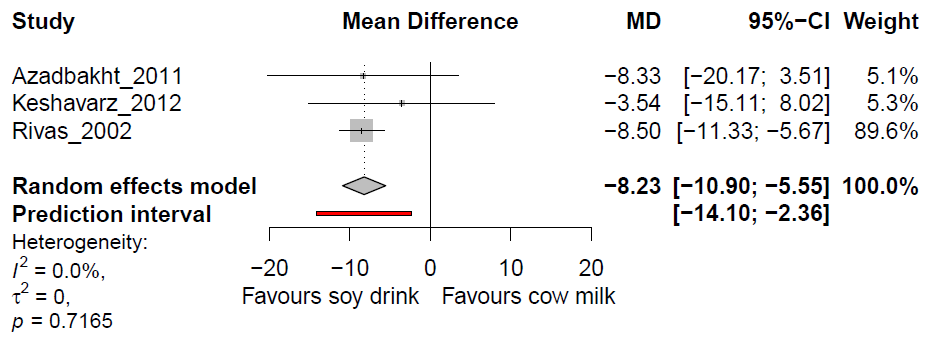


95%CI 95% confidence interval, MD mean difference

# Supplemental Figure 12. Forest Plot summarizing mean differences with 95%CIs

**Pairwise meta-analysis, outcome: diastolic blood pressure (mmHg), comparing soy drink and cow’s milk**


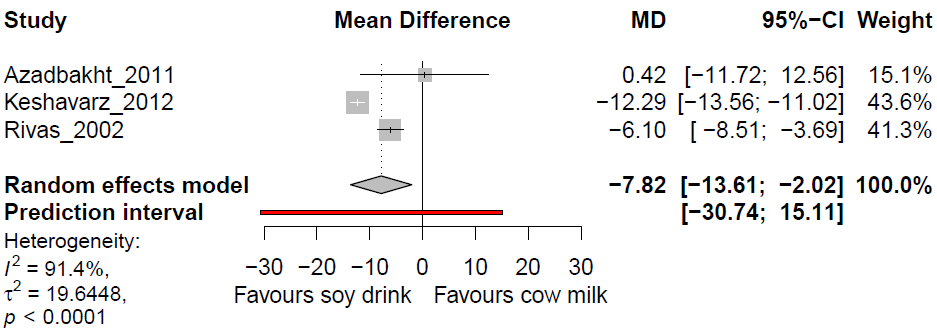


95%CI 95% confidence interval, MD mean difference

# Supplemental Figure 13. Comparison adjusted funnel plot for LDL-cholesterol for the network meta-analysis comparing plant-drinks and cow’s milk


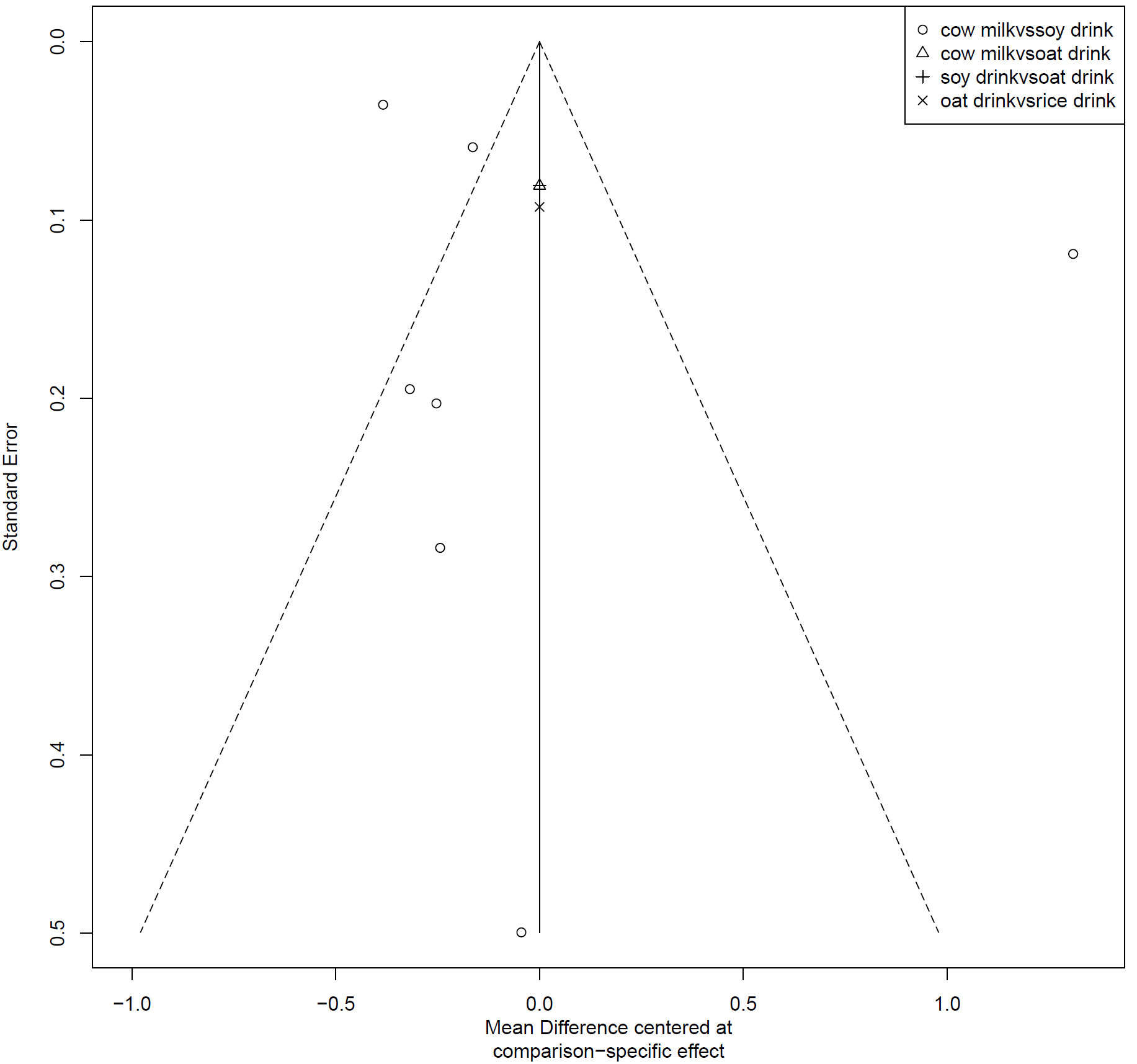


# Supplemental Figure 14. Comparison adjusted funnel plot for total cholesterol for the network meta-analysis comparing plant-drinks and cow’s milk


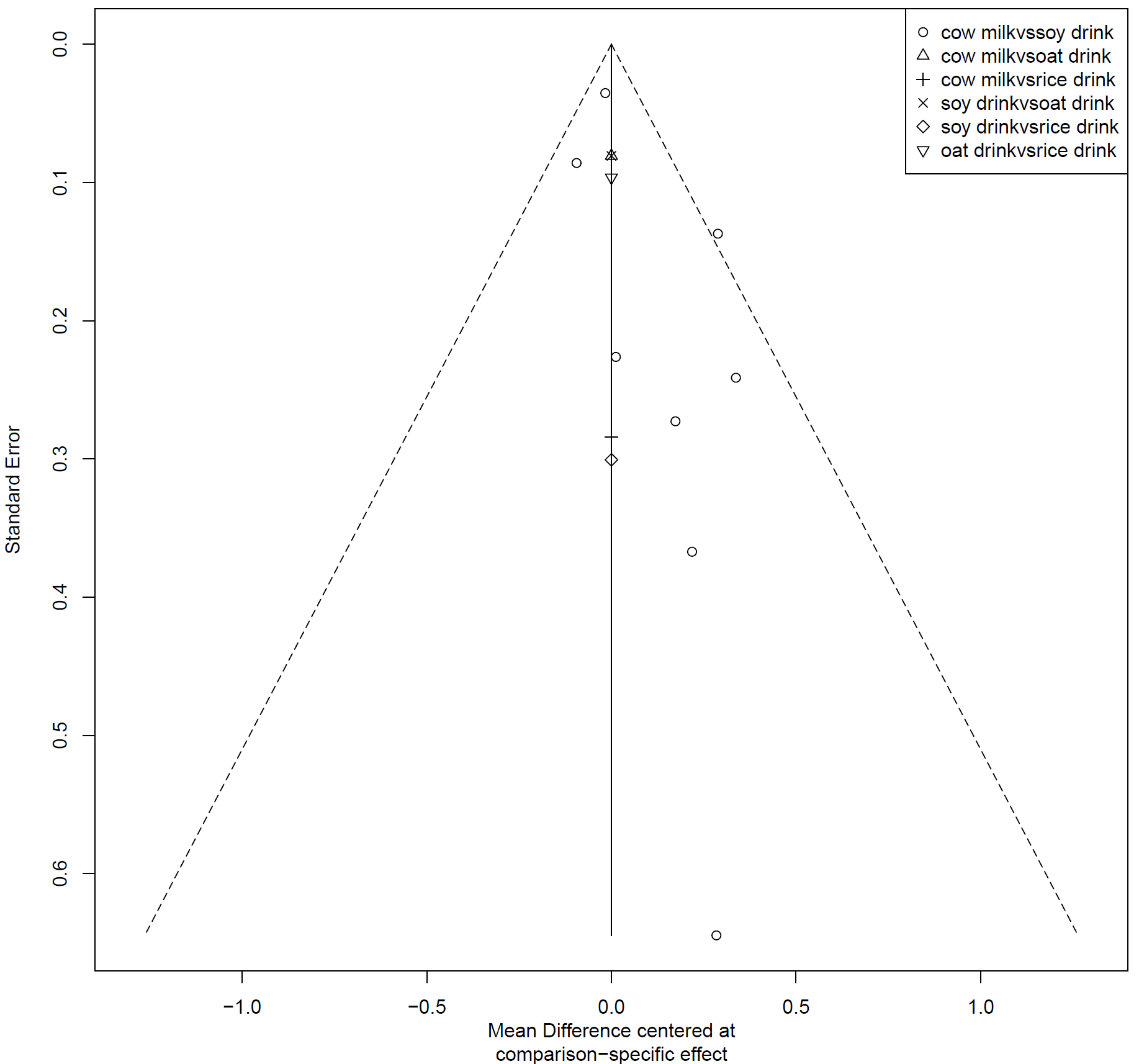


# Supplemental Figure 15. Subgroup Analyses from NMA comparing the effects different plant-drinks with cow’s milk on body weight (kg)


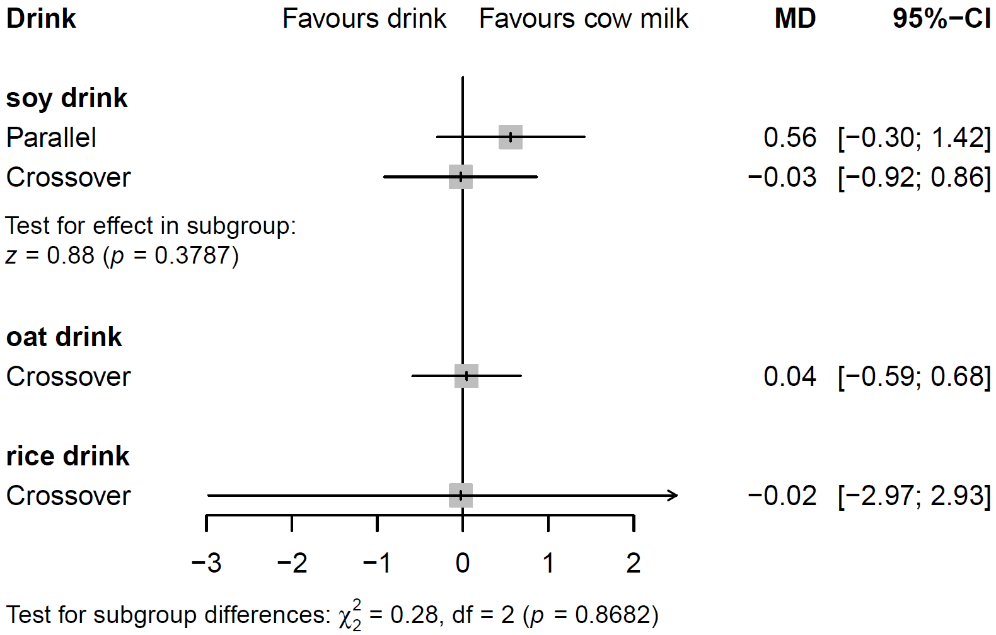


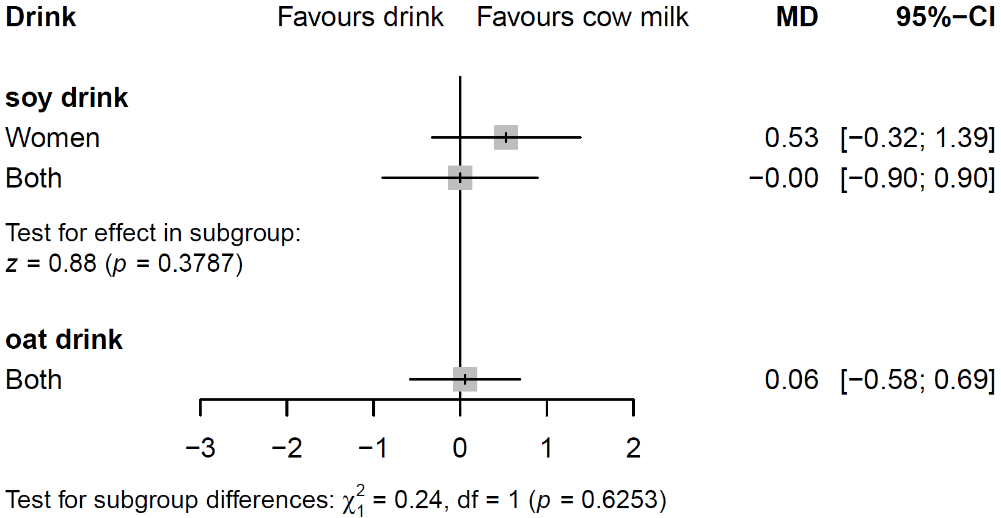

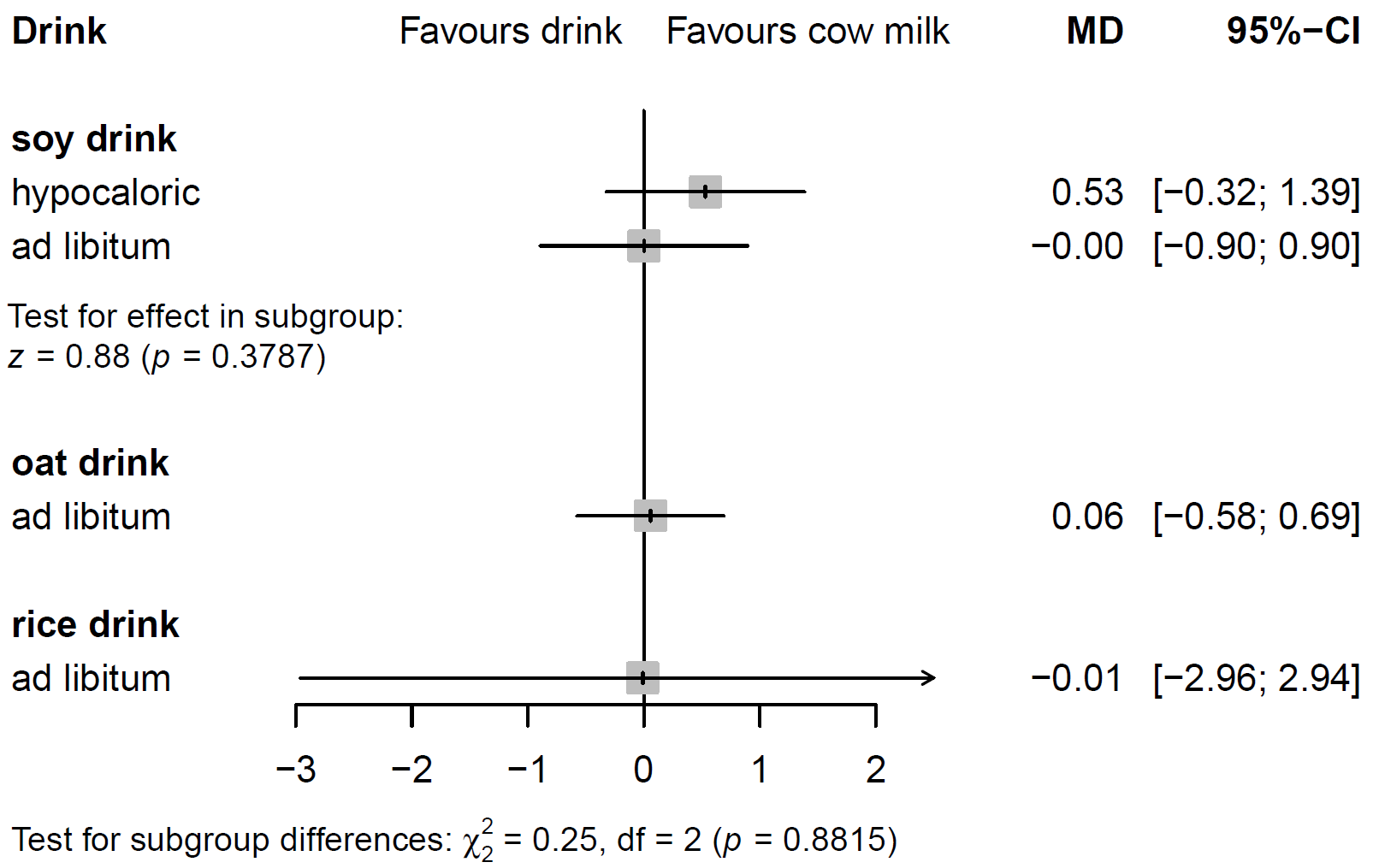


Mean differences (MD) and 95% confidence intervals (95%-CI) are established from dose harmonized (500 ml/d) network meta-analysis (NMA).

# Supplemental Figure 16. Subgroup Analyses from NMA comparing the effects different plant-drinks with cow’s milk on LDL-cholesterol (mmol/L)


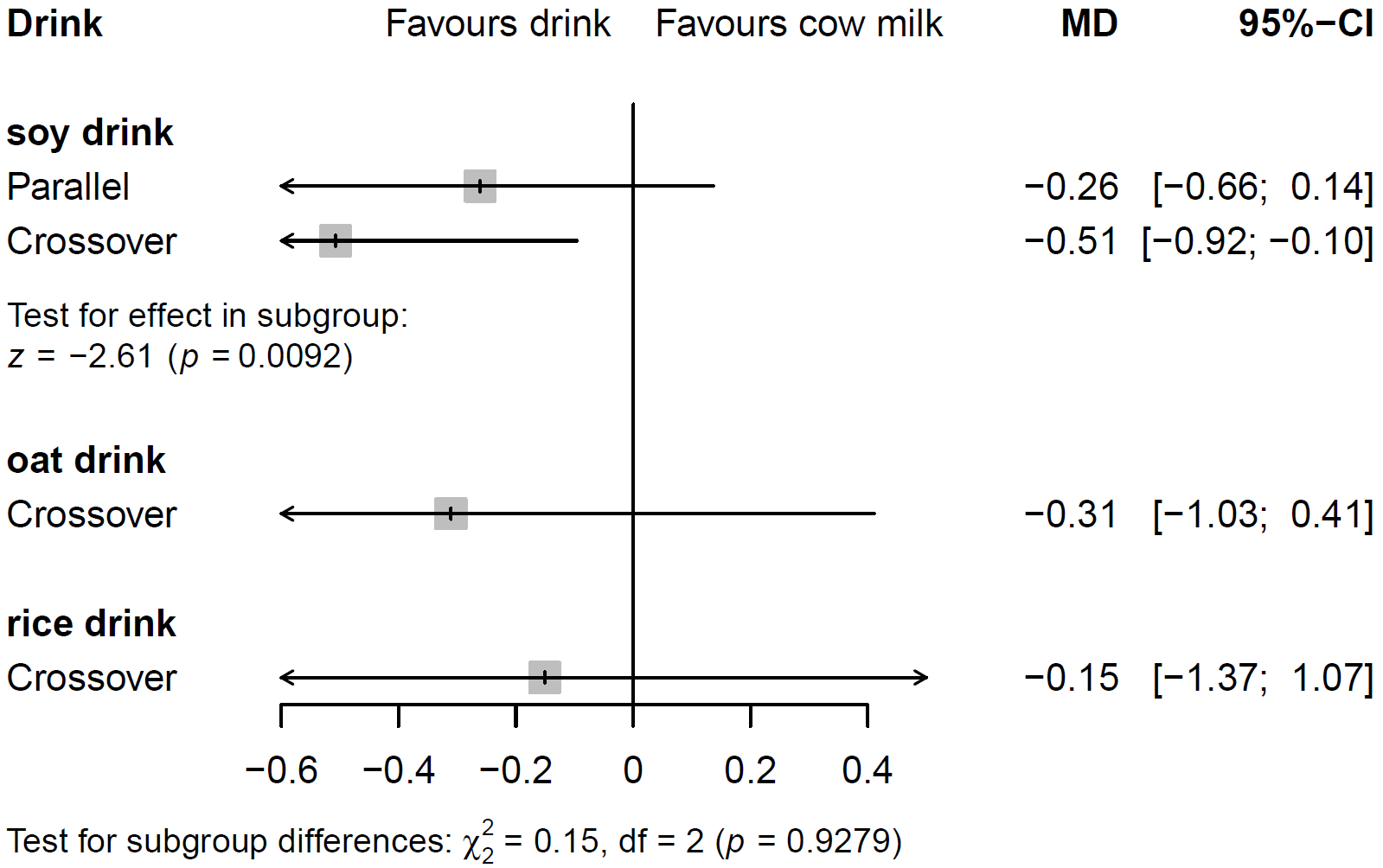

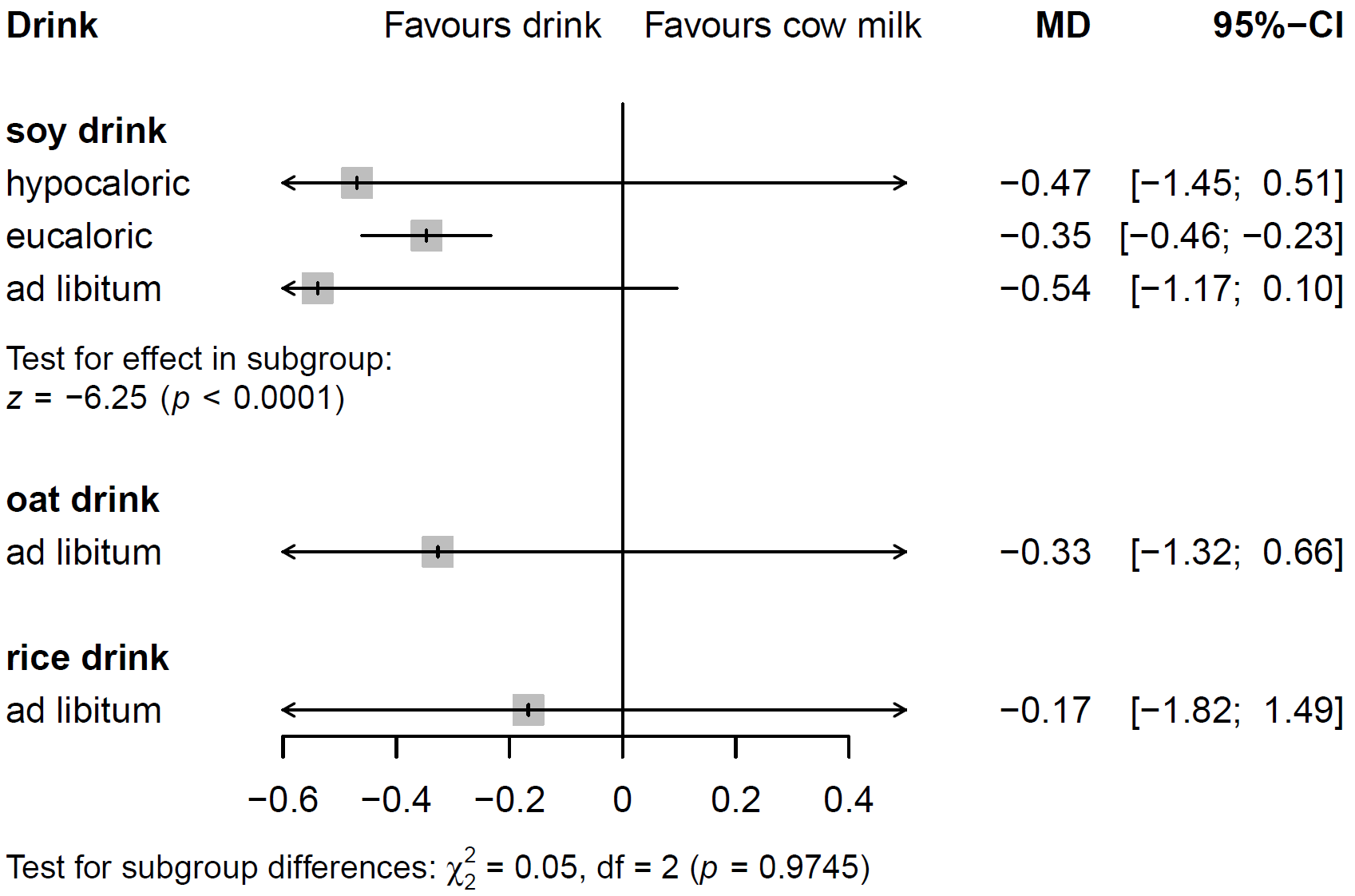


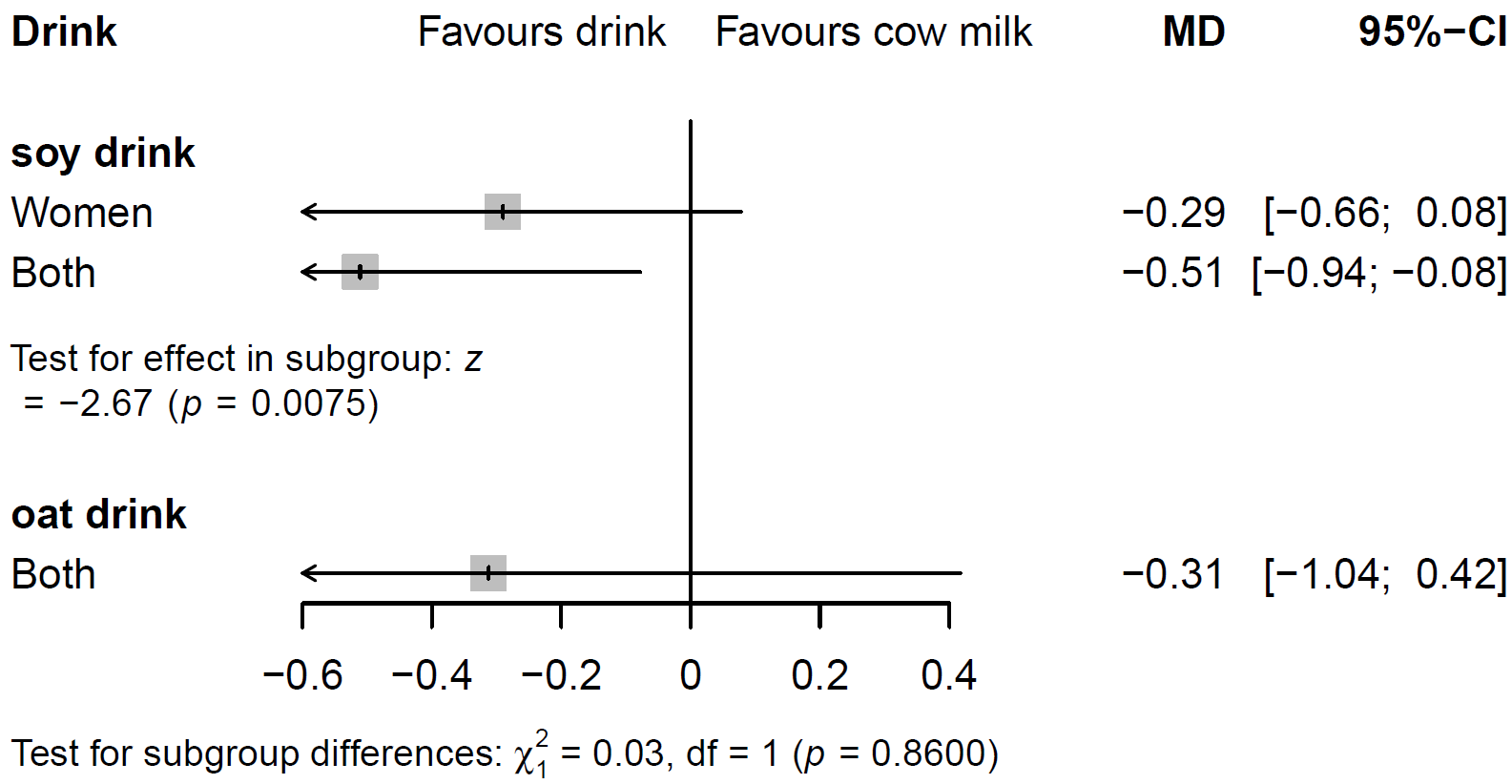
 Mean differences (MD) and 95% confidence intervals (95%-CI) are established from dose harmonized (500 ml/d) network meta-analysis (NMA). LDL low-density lipoprotein

# Supplemental Figure 17. Subgroup Analyses from NMA comparing the effects different plant-drinks with cow’s milk on HDL-cholesterol (mmol/L)


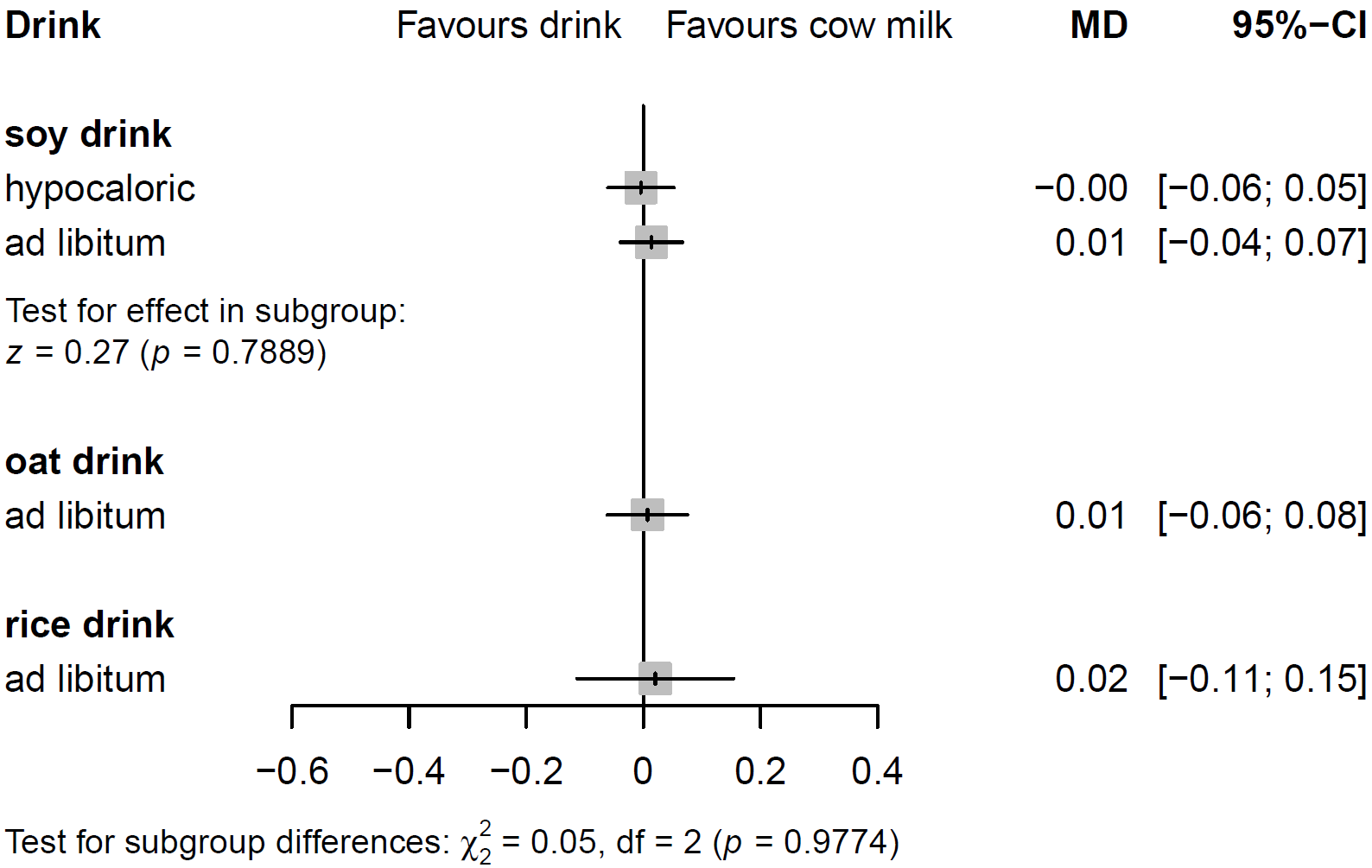

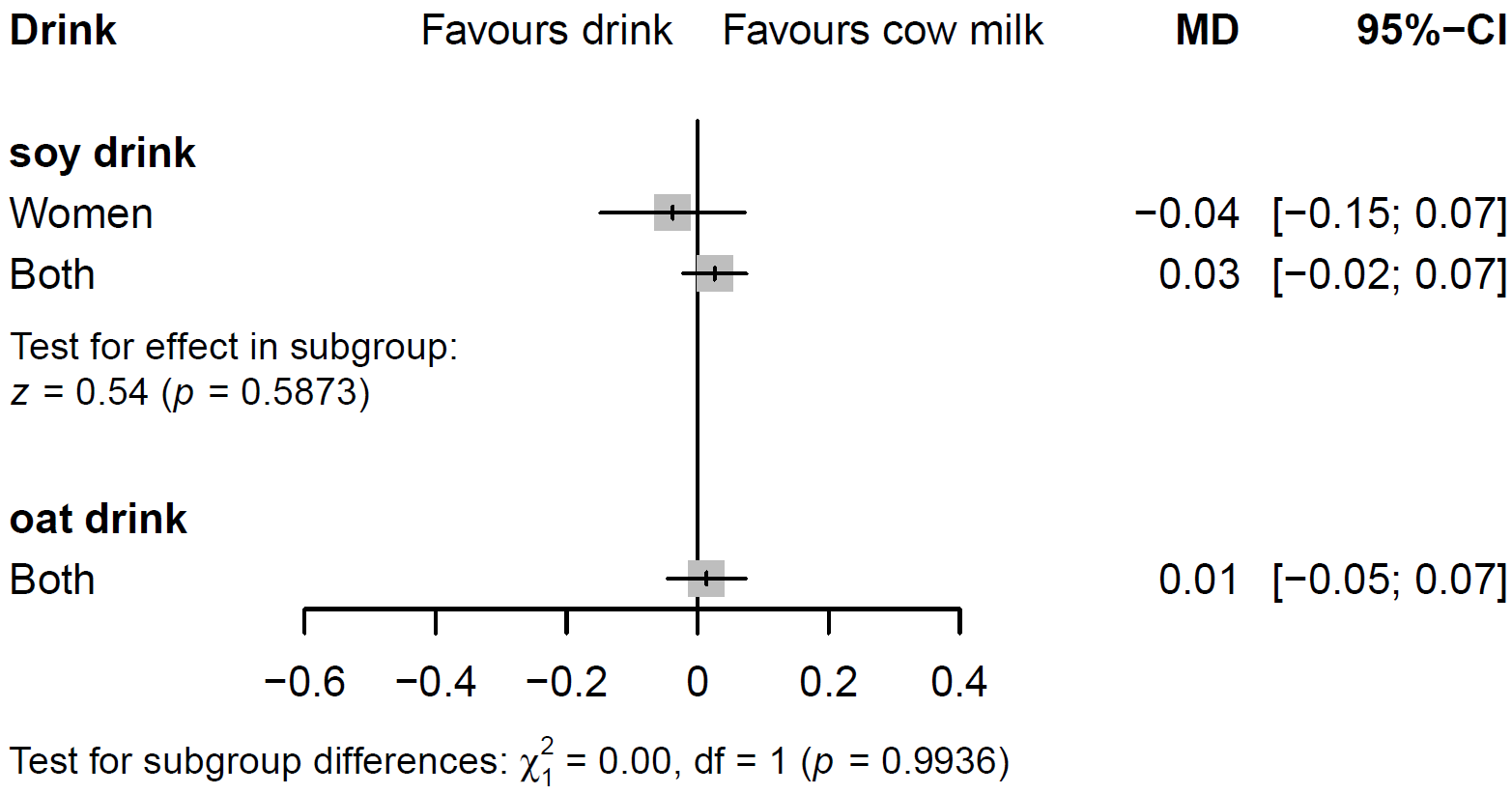


Mean differences (MD) and 95% confidence intervals (95%-CI) are established from dose harmonized (500 ml/d) network meta-analysis (NMA).
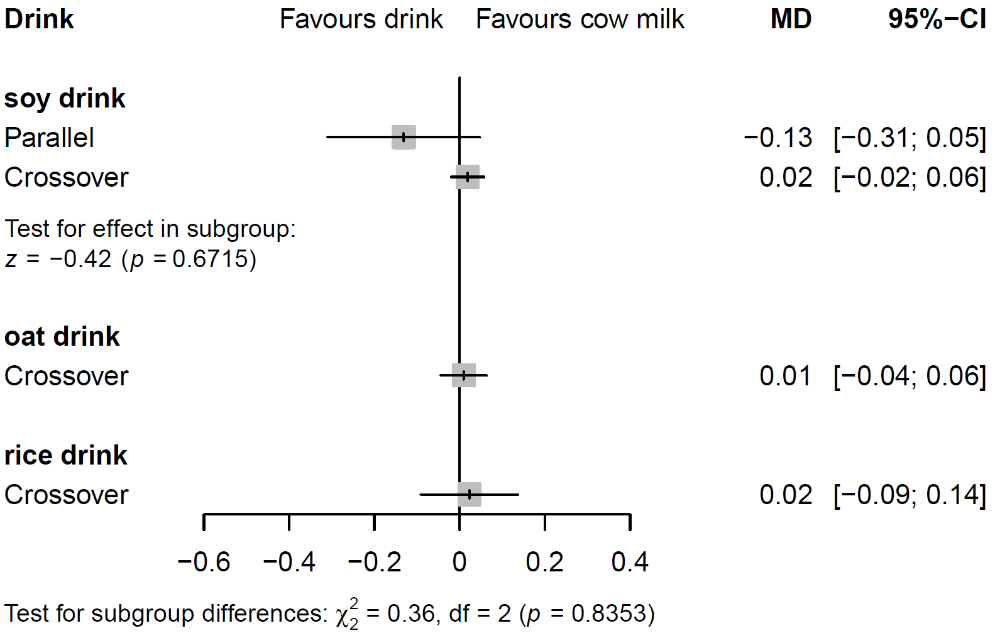
HDL high-density lipoprotein

#
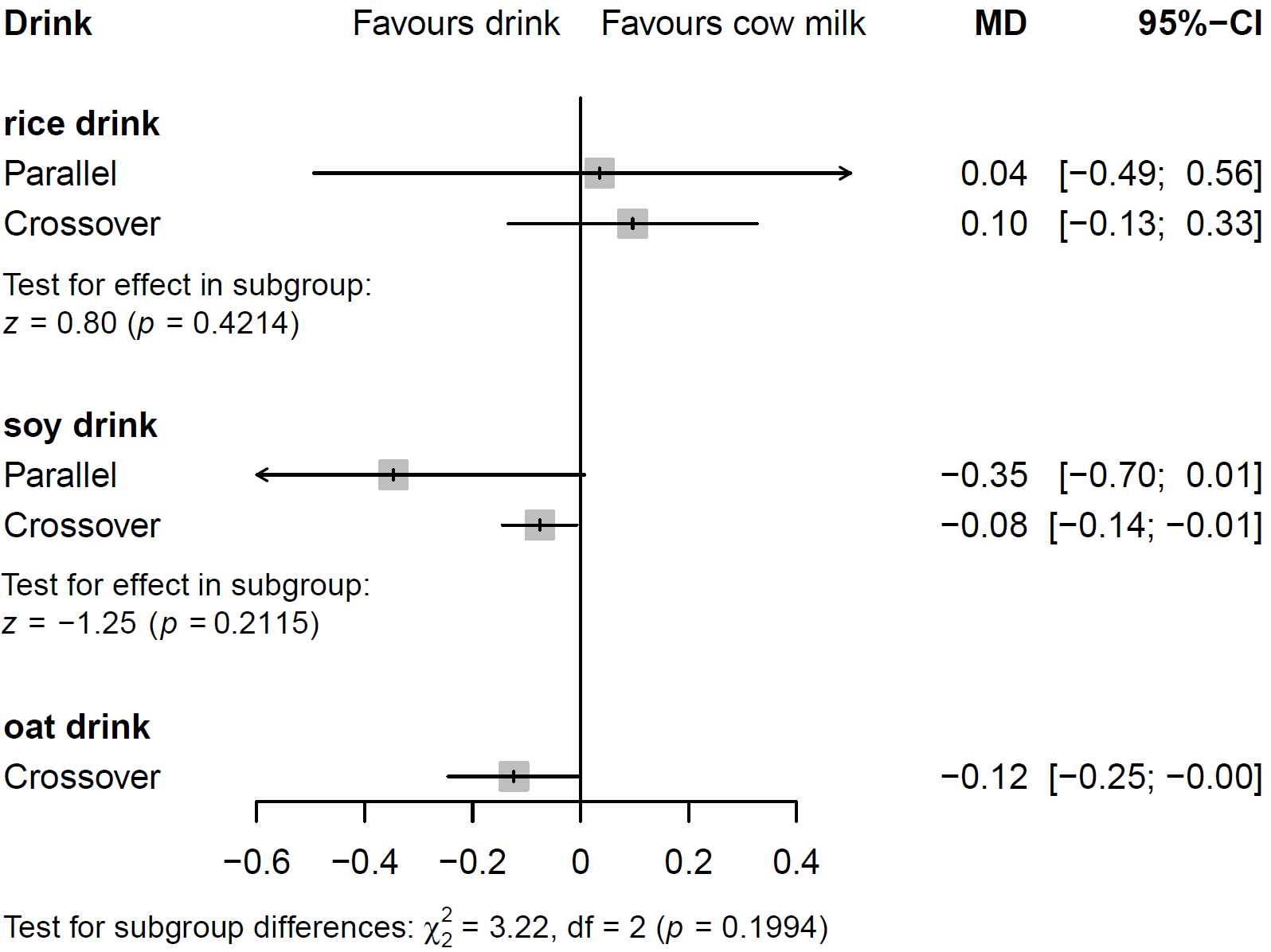
Supplemental Figure 18. Subgroup Analyses from NMA comparing the effects different plant-drinks with cow’s milk on total cholesterol (mmol/L)


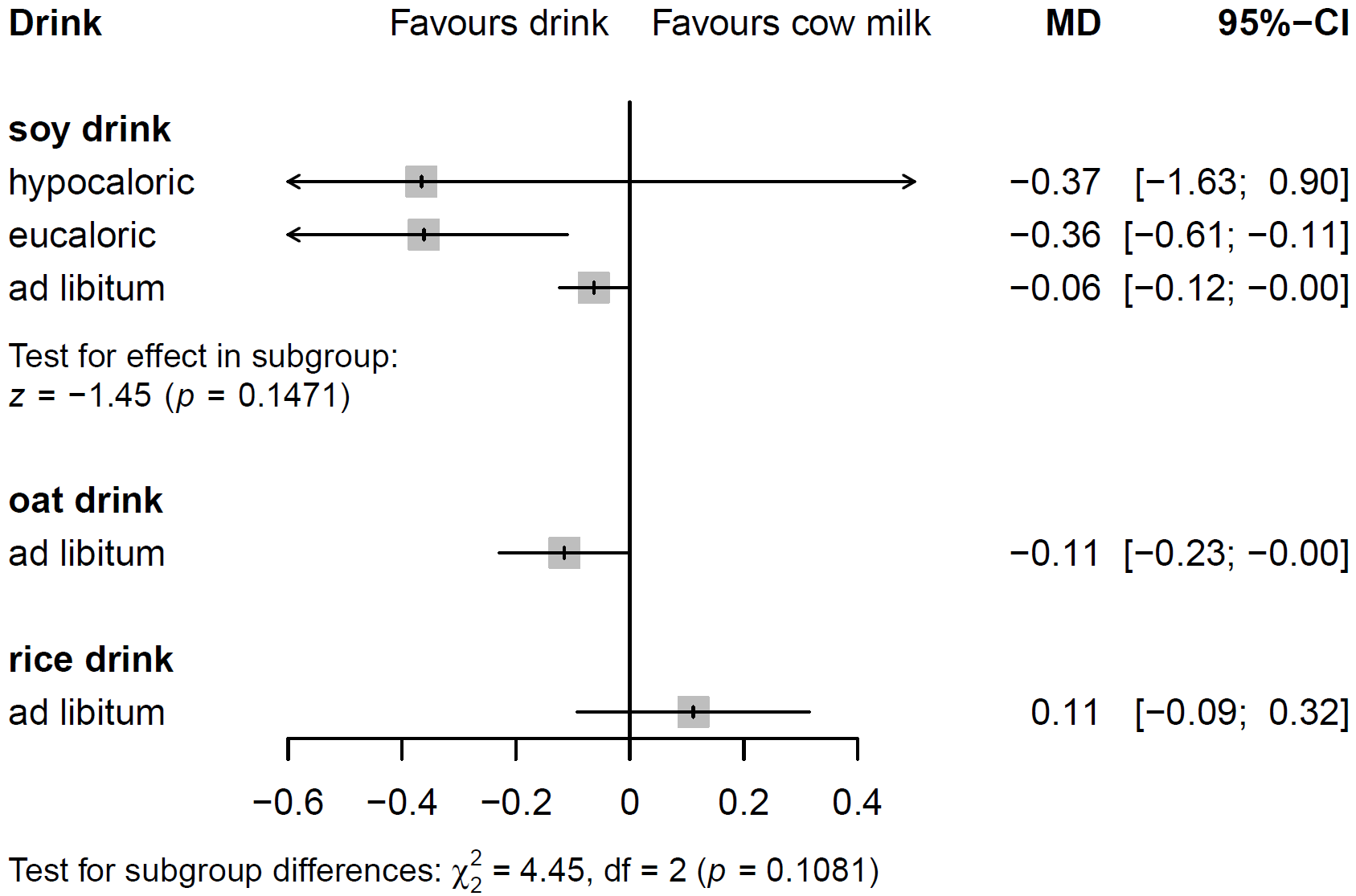


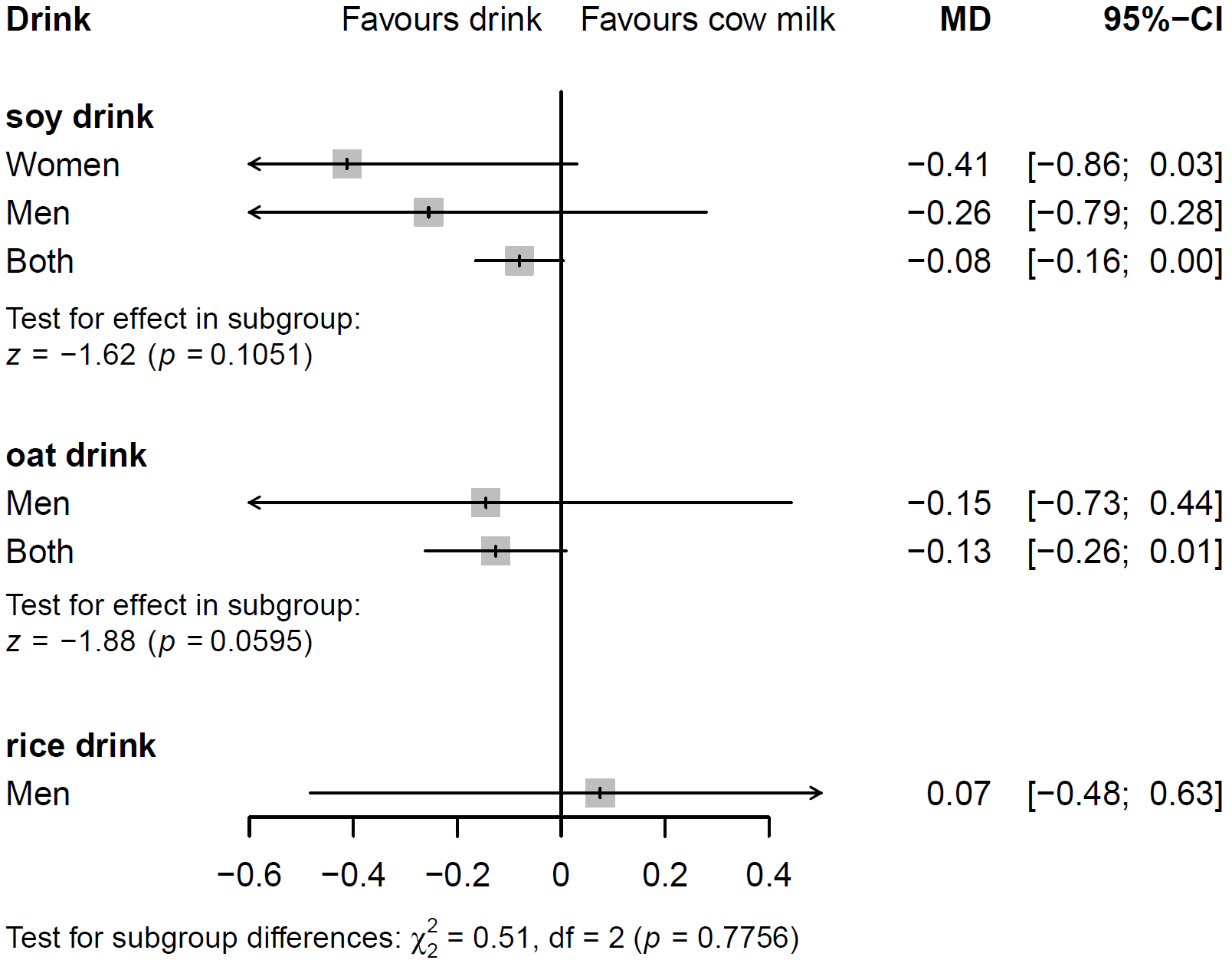


Mean differences (MD) and 95% confidence intervals (95%-CI) are established from dose harmonized (500 ml/d) network meta-analysis (NMA).

# Supplemental Figure 19. Subgroup Analyses from NMA comparing the effects different plant-drinks with cow’s milk on triglycerides (mmol/L)


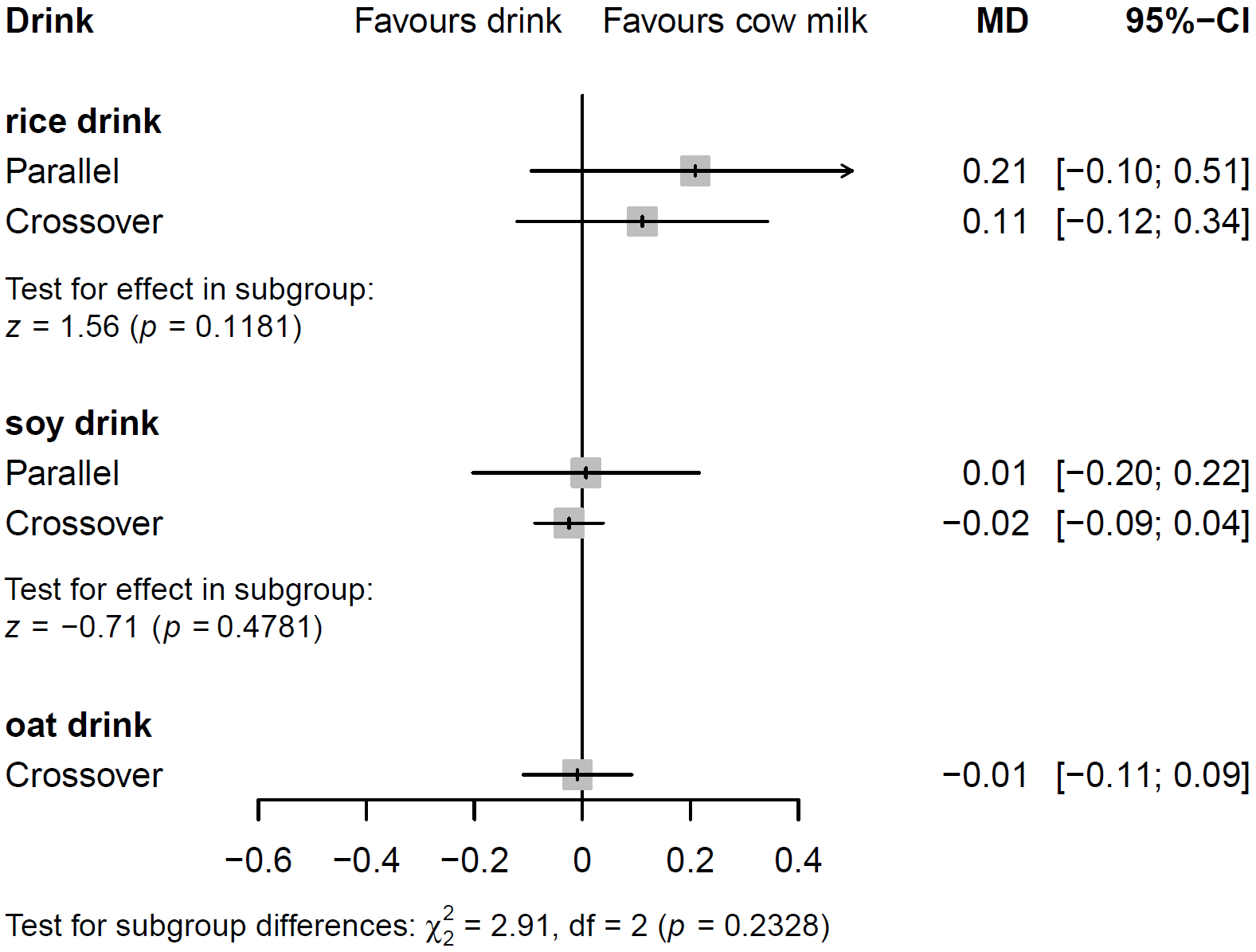

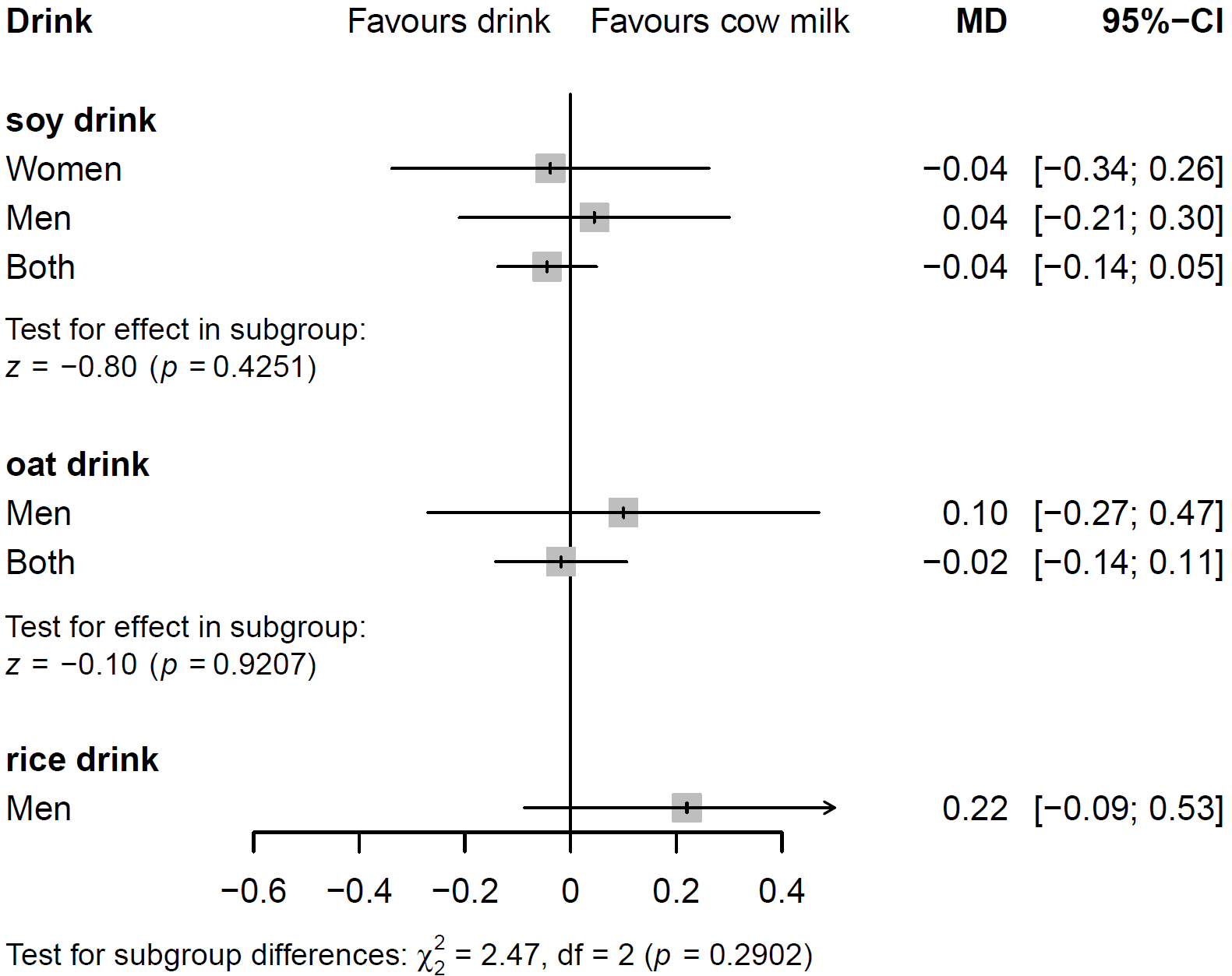


Mean differences (MD) and 95% confidence intervals (95%-CI) are established from dose harmonized (500 ml/d) network meta-analysis (NMA).

# Supplemental Figure 20. Subgroup Analyses from NMA comparing the effects different plant-drinks with cow’s milk on fasting blood glucose (mmol/L)


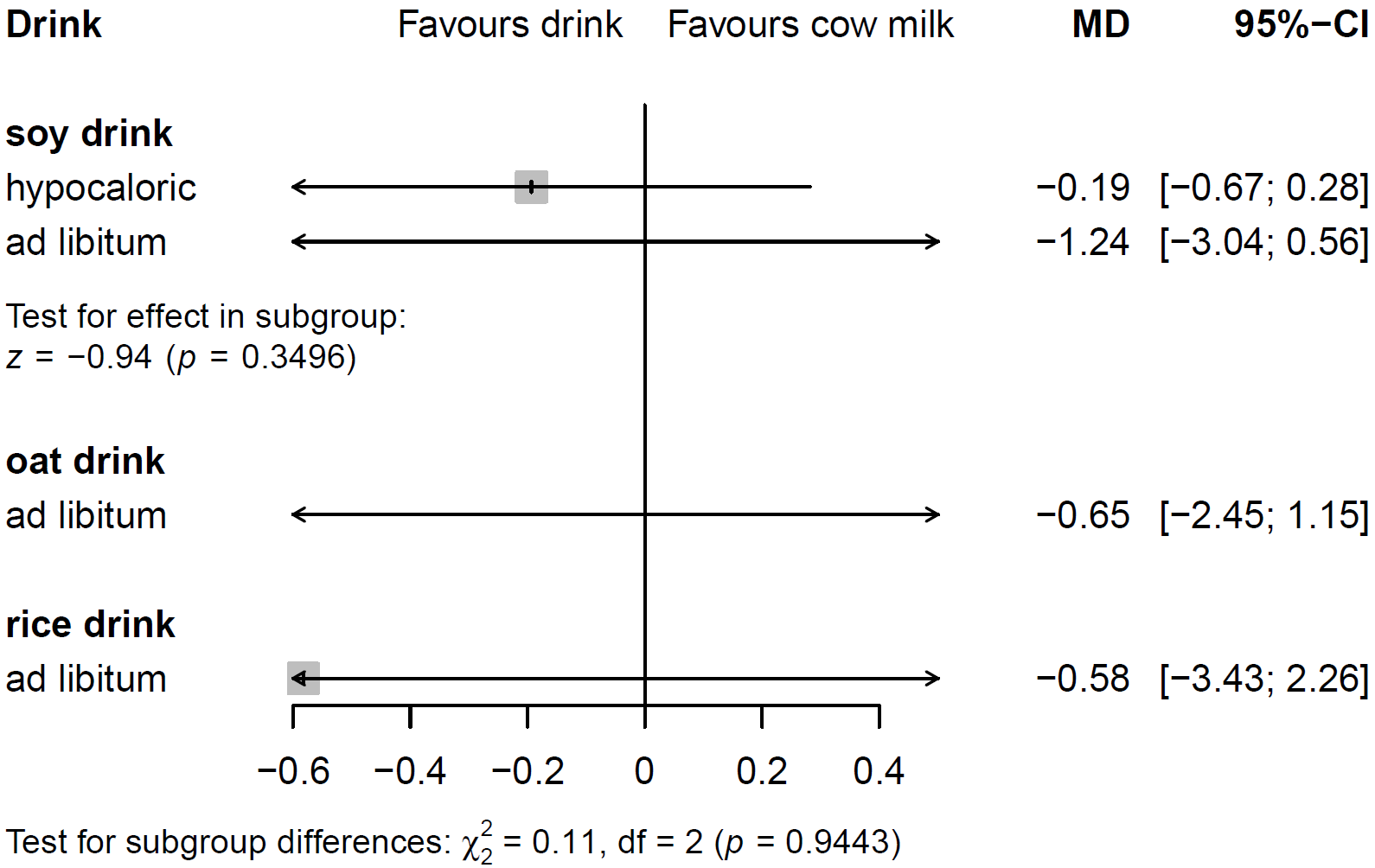

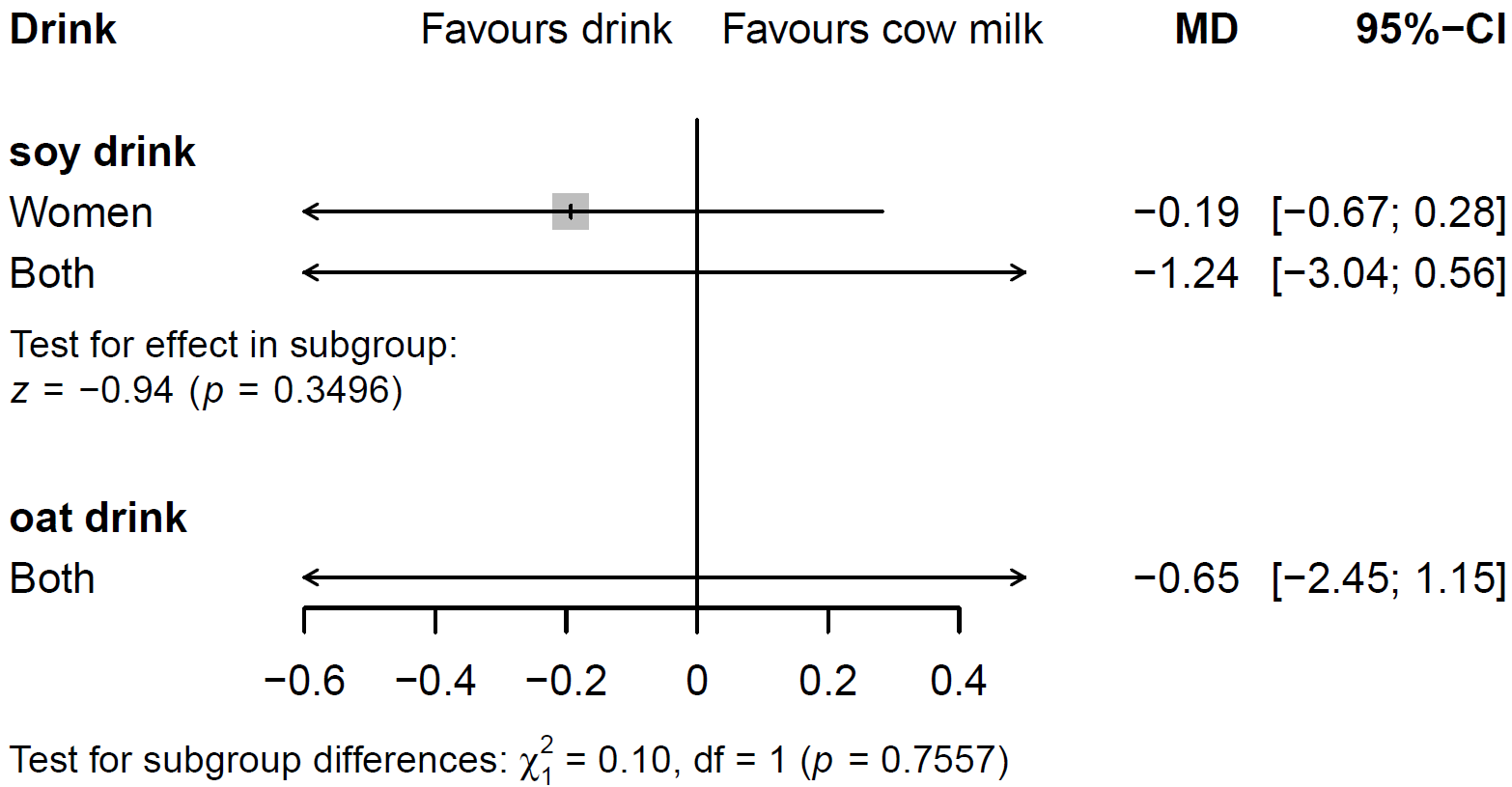


Mean differences (MD) and 95% confidence intervals (95%-CI) are established from dose harmonized (500 ml/d) network meta-analysis (NMA).

# Supplemental Figure 21. Sensitivity analysis regarding risk of bias

**Harmonized NMA, outcome: body weight (kg), comparing the effects different plant-drinks with cow’s milk**


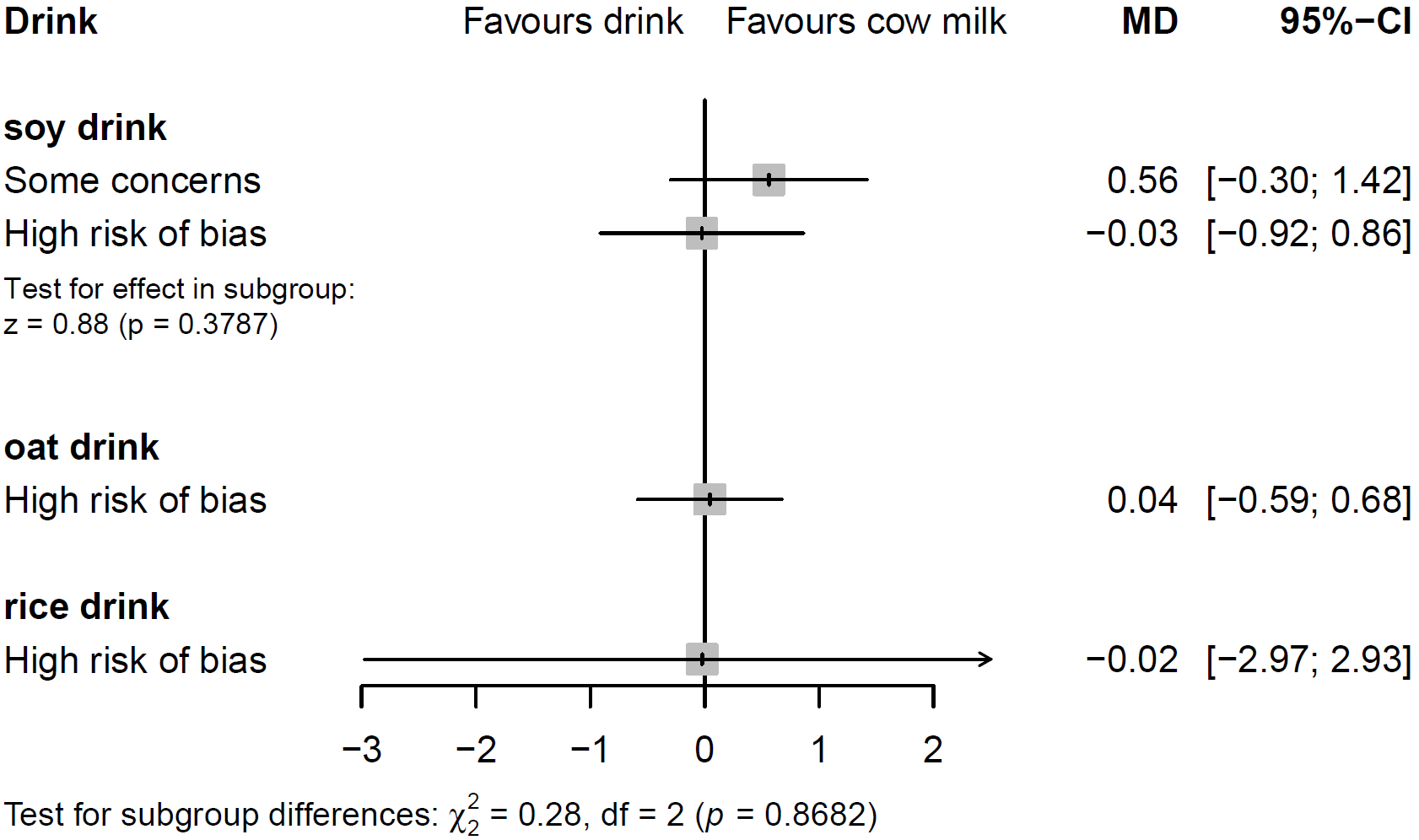


Mean differences (MD) and 95% confidence intervals (95%-CI) are established from dose harmonized (500 ml/d) network meta-analysis (NMA).

# Supplemental Figure 22. Sensitivity analysis regarding risk of bias

**Harmonized NMA, outcome: LDL-cholesterol (mmol/L), comparing the effects different plant-drinks with cow’s milk**


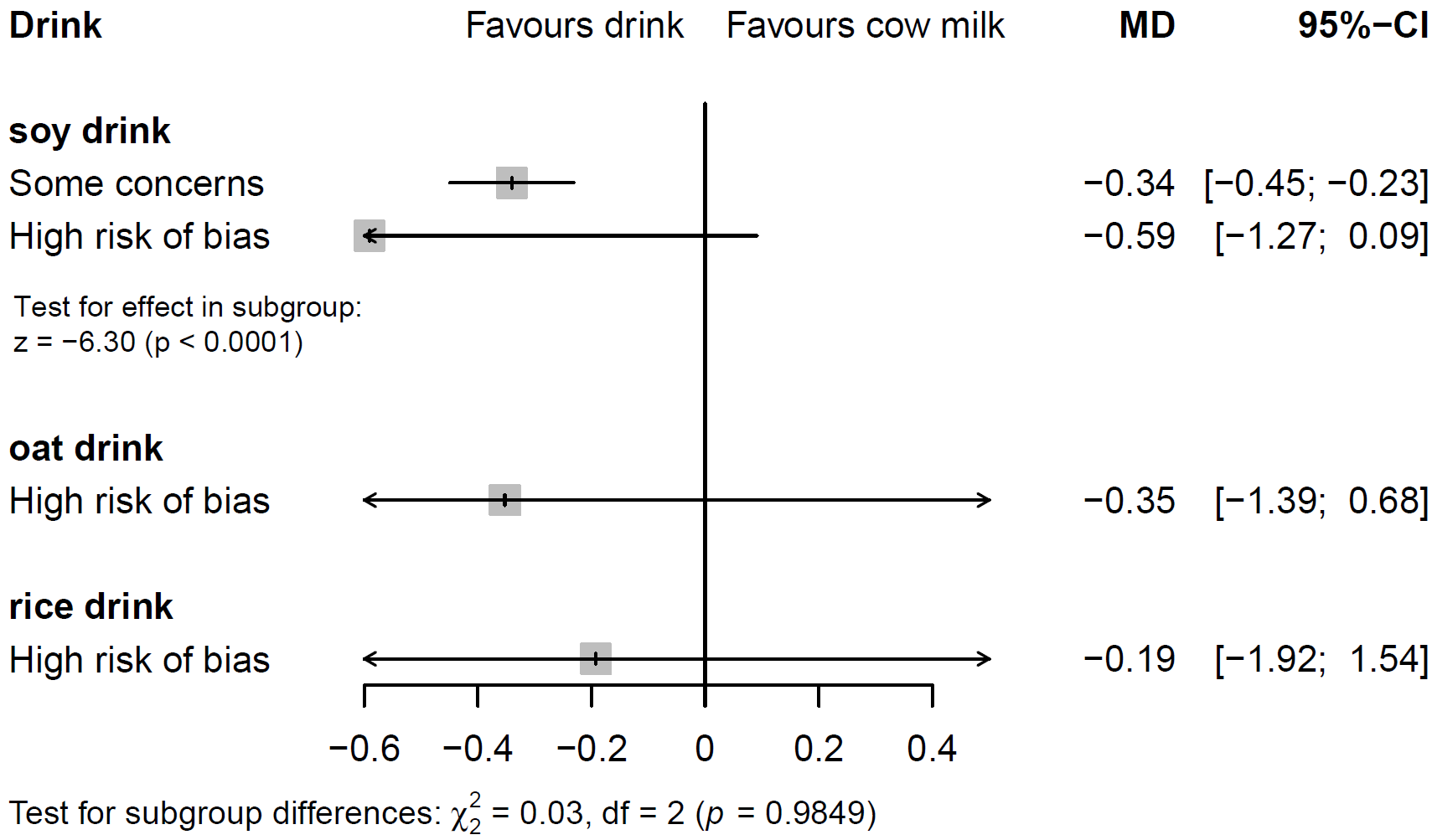
 Mean differences (MD) and 95% confidence intervals (95%-CI) are established from dose harmonized (500 ml/d) network meta-analysis (NMA). LDL low-density lipoprotein

# Supplemental Figure 23. Sensitivity analysis regarding risk of bias

**Harmonized NMA, outcome: HDL-cholesterol (mmol/L), comparing the effects different plant-drinks with cow’s milk**
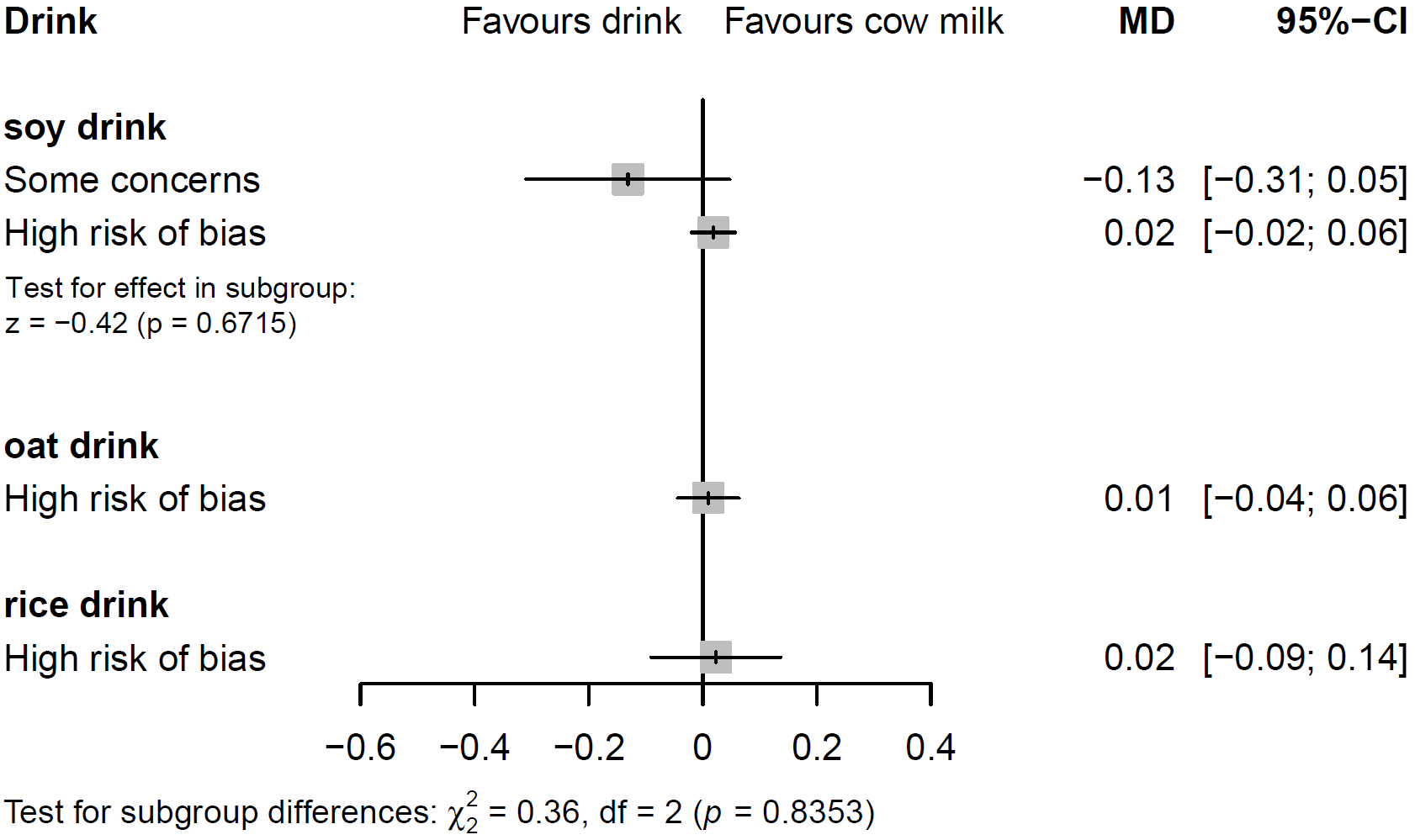


Mean differences (MD) and 95% confidence intervals (95%-CI) are established from dose harmonized (500 ml/d) network meta-analysis (NMA). HDL high-density lipoprotein

# Supplemental Figure 24. Sensitivity analysis regarding risk of bias

**Harmonized NMA, outcome: total cholesterol (mmol/L), comparing the effects different plant-drinks with cow’s milk**


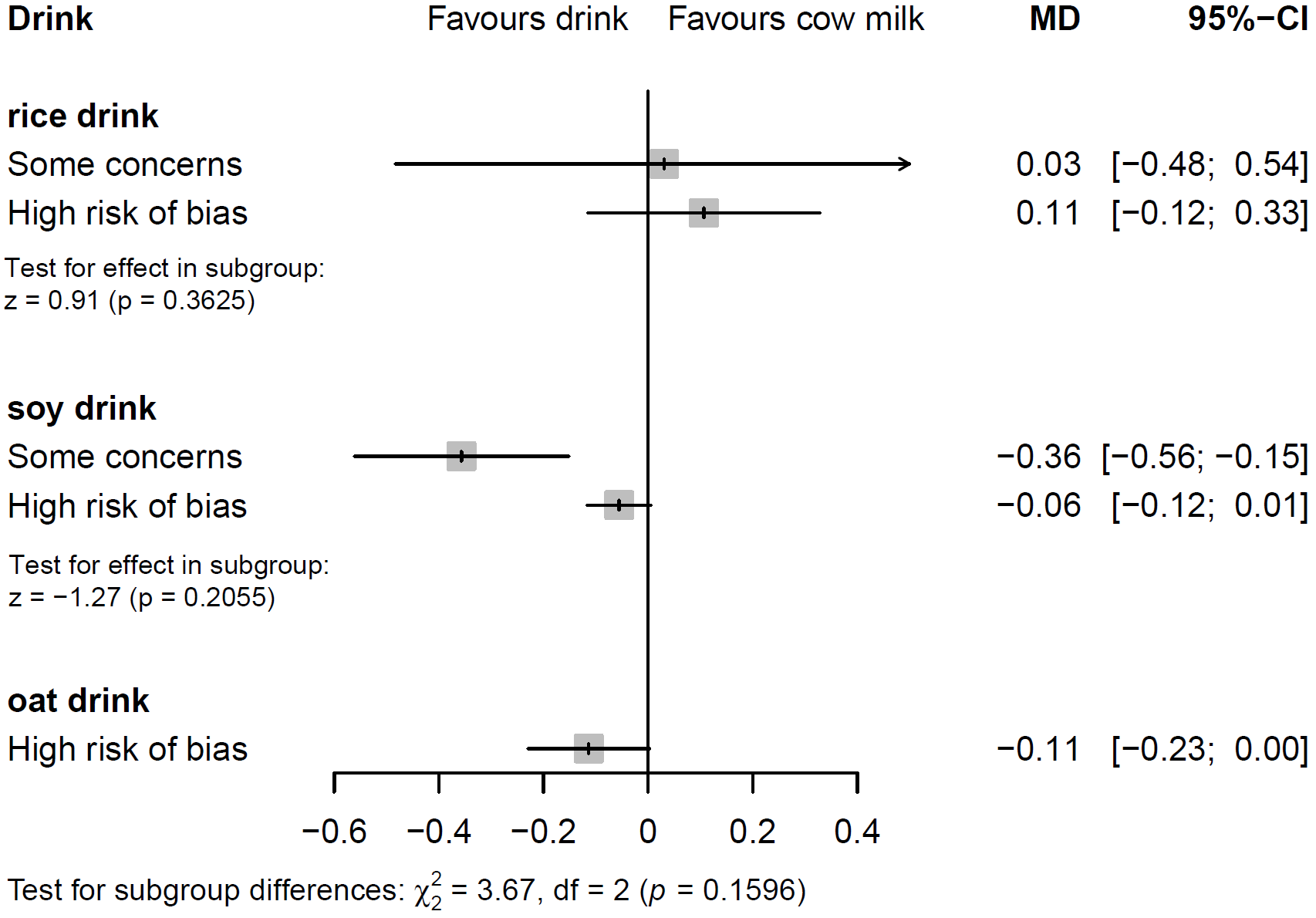
 Mean differences (MD) and 95% confidence intervals (95%-CI) are established from dose harmonized (500 ml/d) network meta-analysis (NMA).

# Supplemental Figure 25. Sensitivity analysis regarding risk of bias

**Harmonized NMA, outcome: triglycerides (mmol/L), comparing the effects different plant-drinks with cow’s milk**


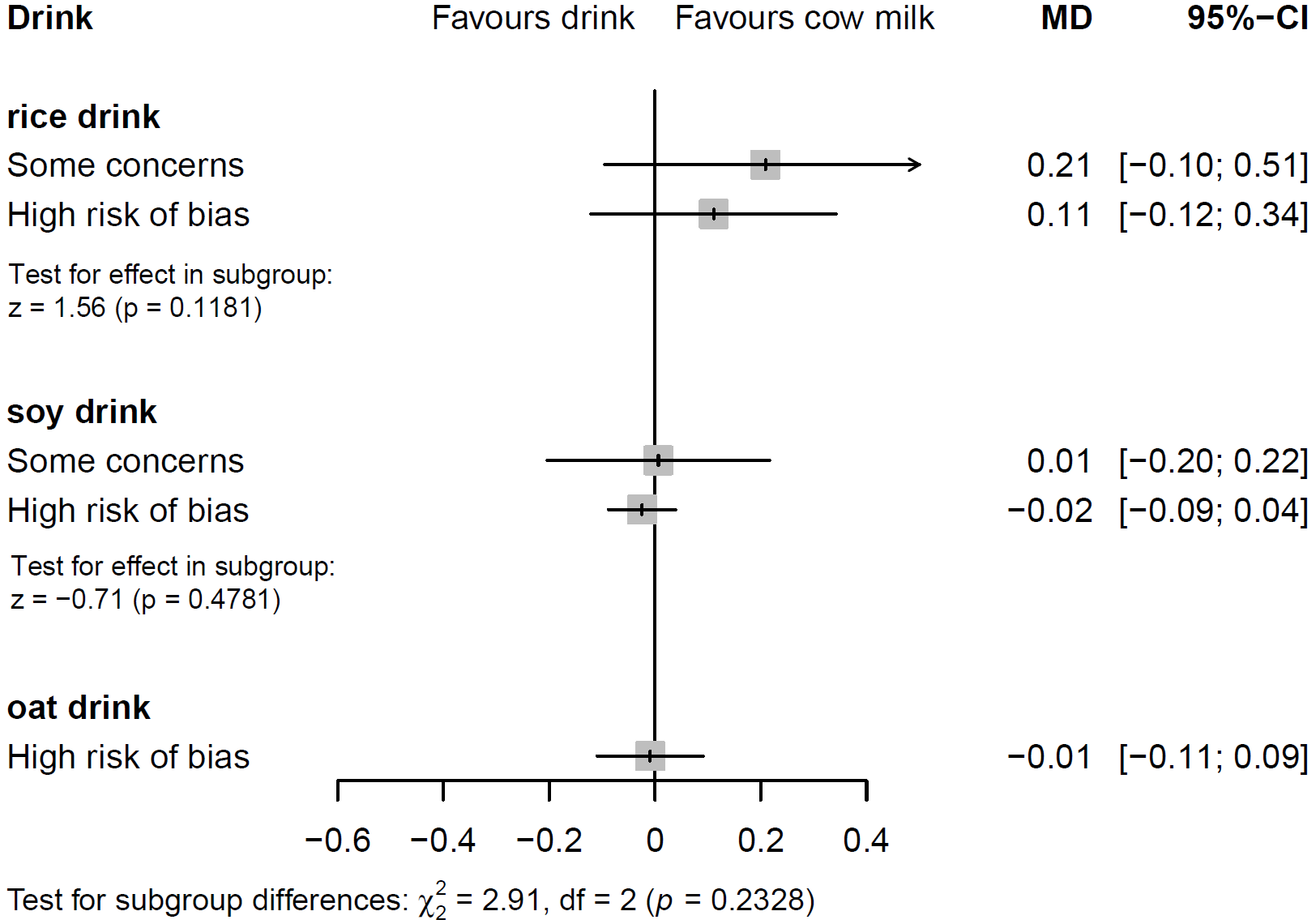


Mean differences (MD) and 95% confidence intervals (95%-CI) are established from dose harmonized (500 ml/d) network meta-analysis (NMA).

# Supplemental Figure 26. Sensitivity analysis harmonized NMA with post values

**Outcome: triglycerides (mmol/L)**


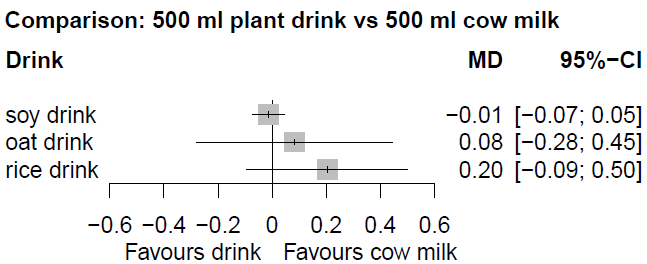


95%-CI 95% confidence interval, MD mean difference

MD mean differences established from network meta-analysis (NMA) harmonized doses (500 ml/d)

# Supplemental Figure 27. Sensitivity analysis harmonized NMA with post values

**Outcome: total cholesterol (mmol/L)**


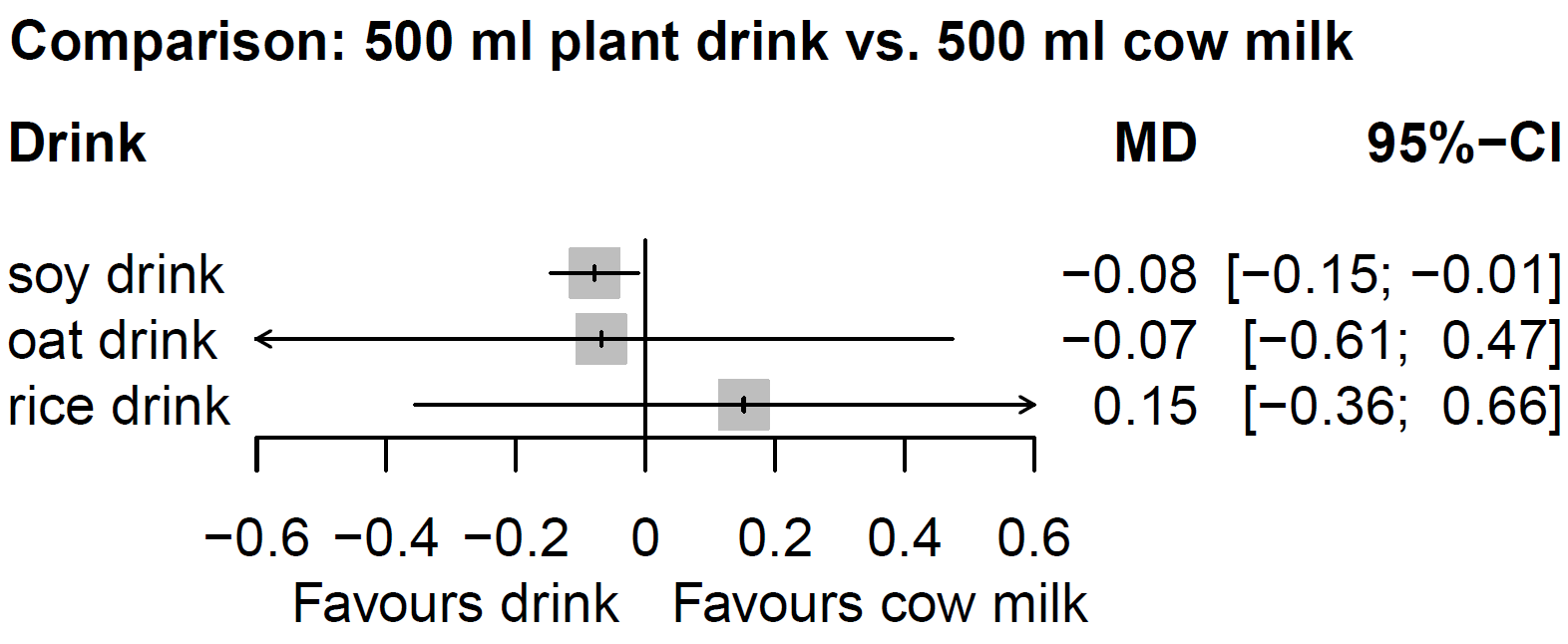


95%-CI 95% confidence interval, MD mean difference

MD mean differences established from network meta-analysis (NMA) harmonized doses (500 ml/d)

# Supplemental Figure 28. Sensitivity analysis harmonized NMA with change scores only

**Outcome: body weight (kg)**


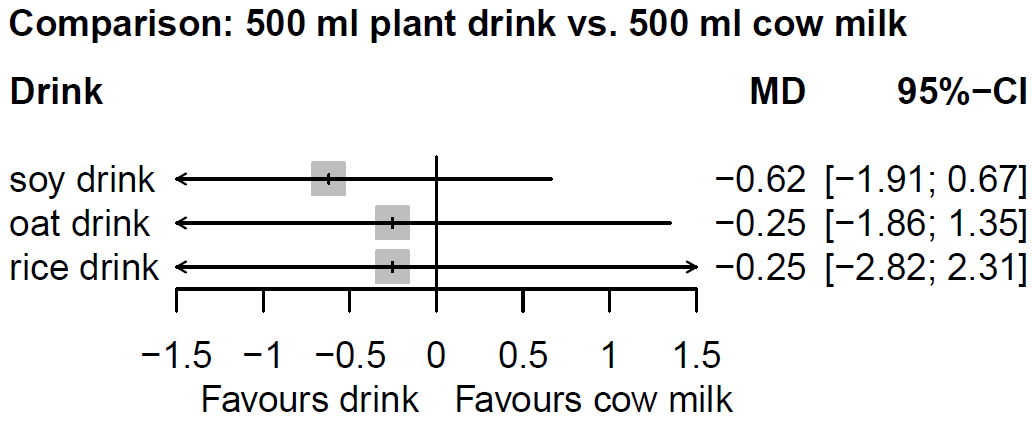


95%-CI 95% confidence interval, MD mean difference

MD mean differences established from network meta-analysis (NMA) harmonized doses (500 ml/d)

# Supplemental Figure 29. Sensitivity analysis harmonized NMA with change scores only

**Outcome: LDL-cholesterol (mmol/L)**


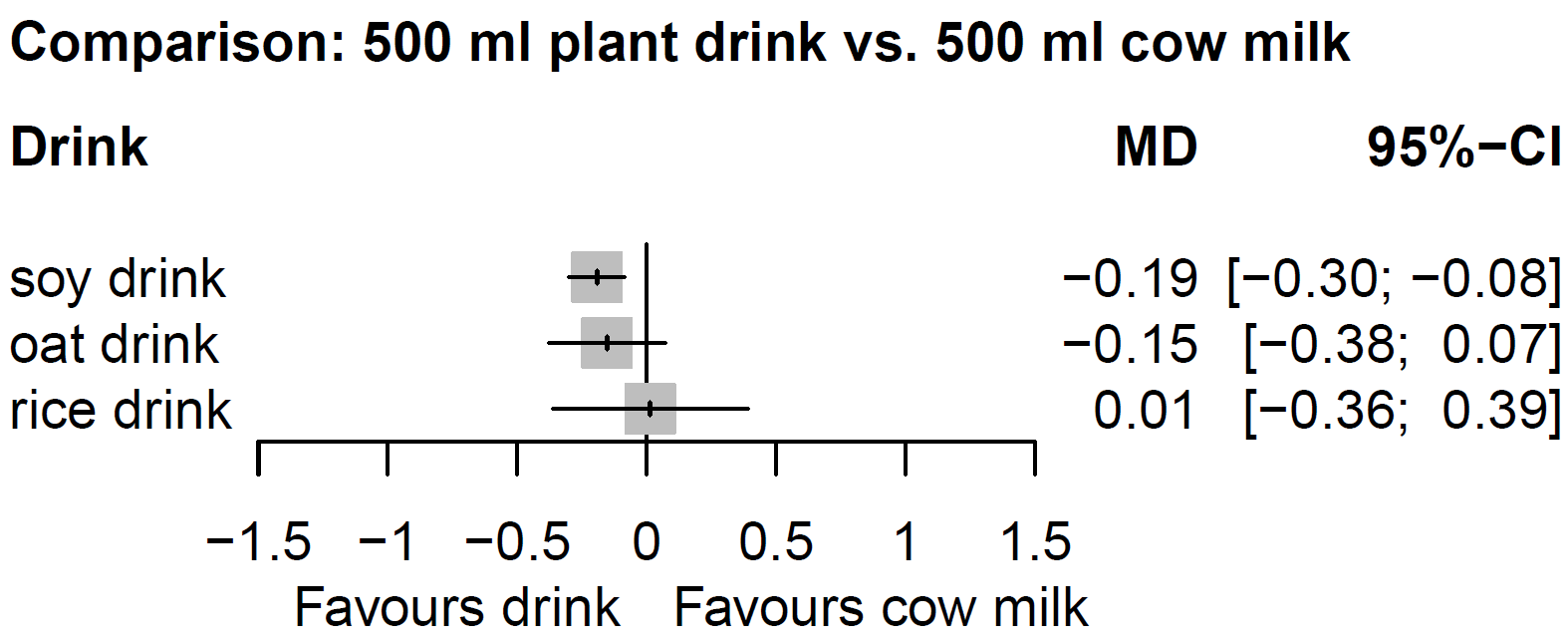


95%-CI 95% confidence interval, MD mean difference

MD mean differences established from network meta-analysis (NMA) harmonized doses (500 ml/d)

# Supplemental Figure 30. Sensitivity analysis harmonized NMA with change scores only

**Outcome: HDL-cholesterol (mmol/L)**


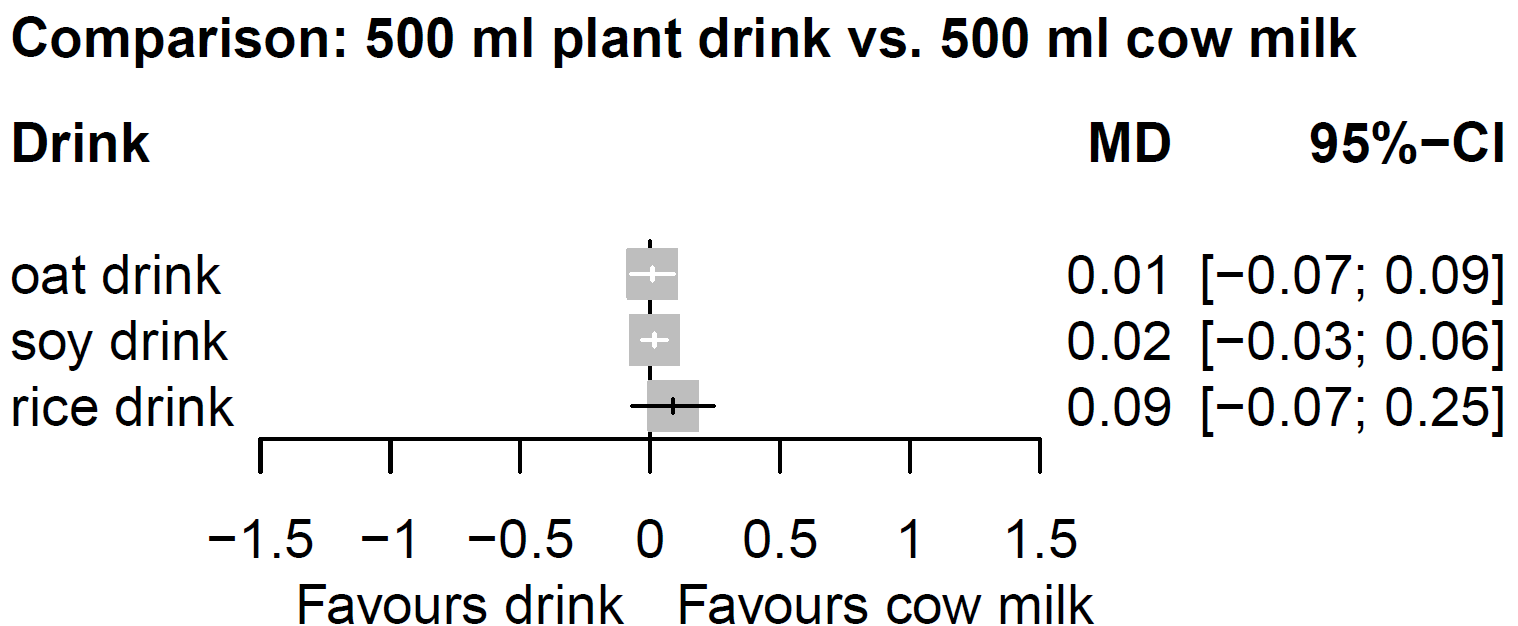


95%-CI 95% confidence interval, MD mean difference

MD mean differences established from network meta-analysis (NMA) harmonized doses (500 ml/d)

# Supplemental Figure 31. Sensitivity analysis harmonized NMA with change scores only

**Outcome: triglycerides (mmol/L)**


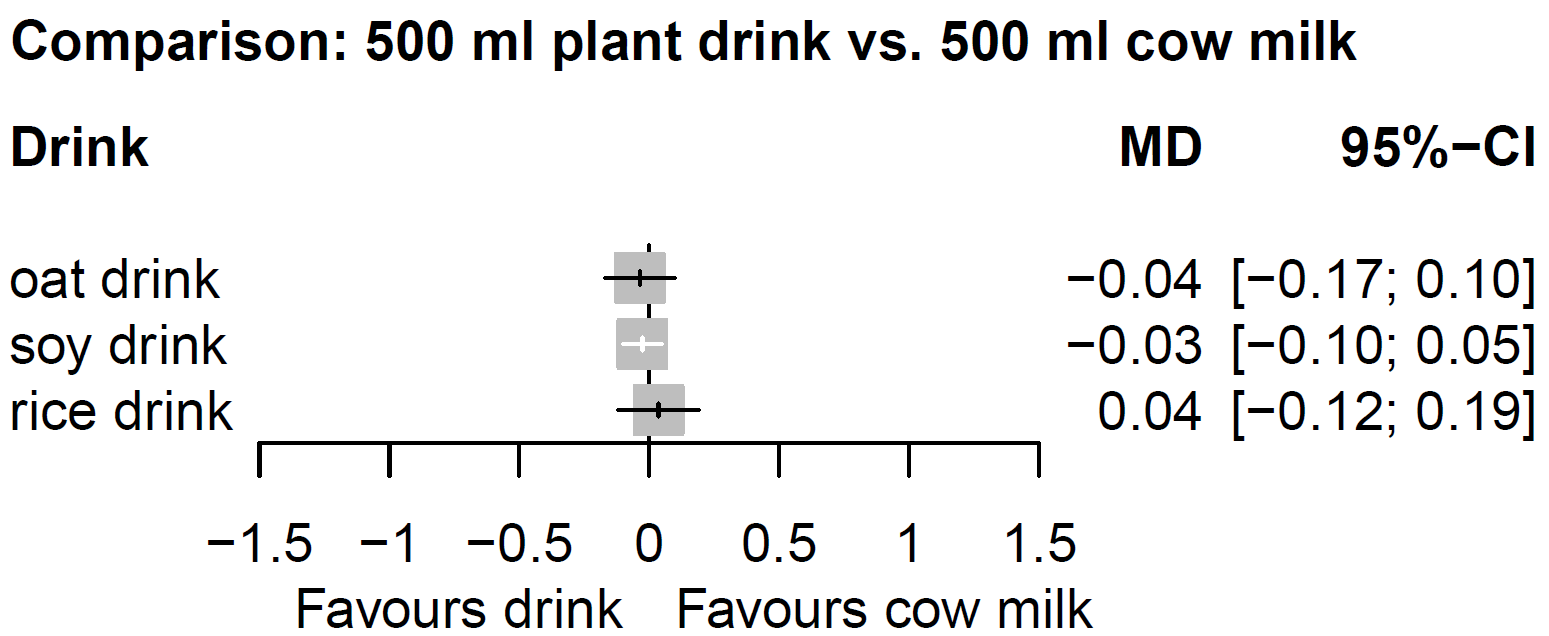


95%-CI 95% confidence interval, MD mean difference

MD mean differences established from network meta-analysis (NMA) harmonized doses (500 ml/d)

# Supplemental Figure 32. Sensitivity analysis harmonized NMA with change scores only

**Outcome: total cholesterol (mmol/L)**


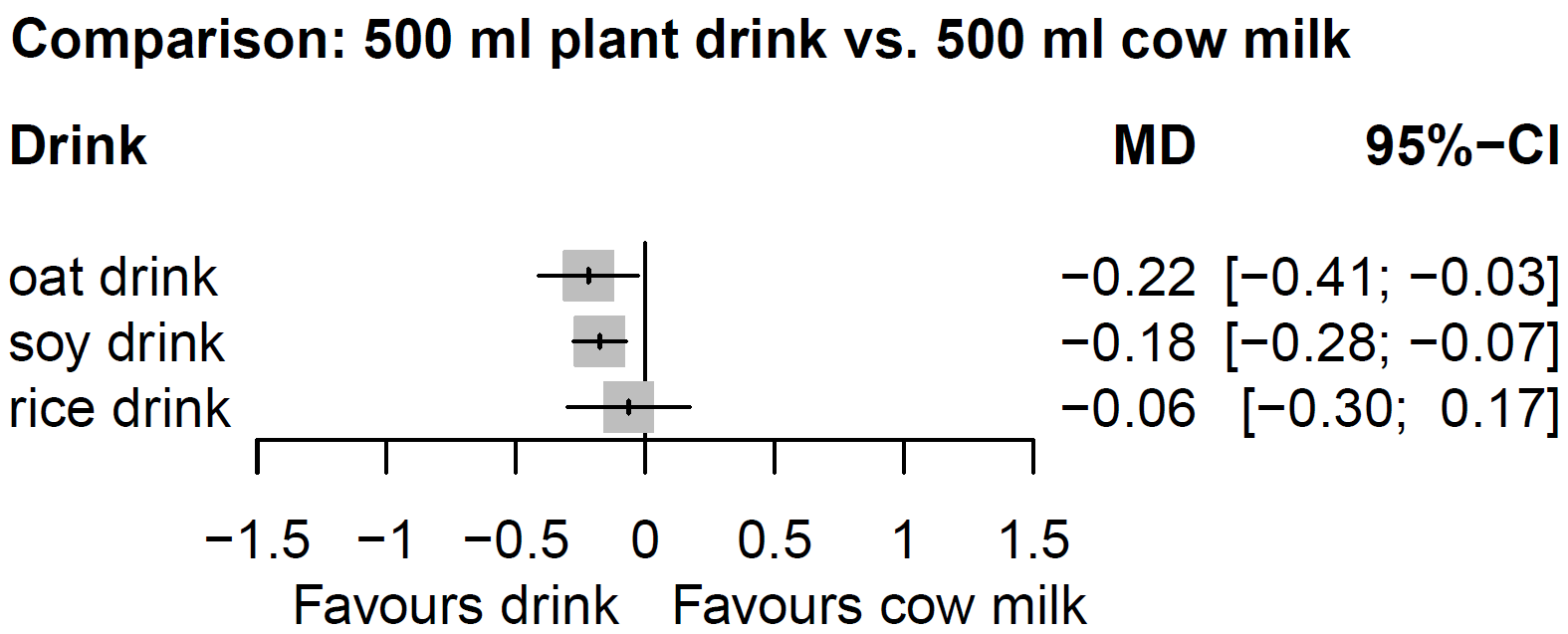


95%-CI 95% confidence interval, MD mean difference

MD mean differences established from network meta-analysis (NMA) harmonized doses (500 ml/d)

# Supplemental Figure 33. Sensitivity analysis harmonized NMA with change scores only

**Outcome: fasting blood glucose (mmol/L)**


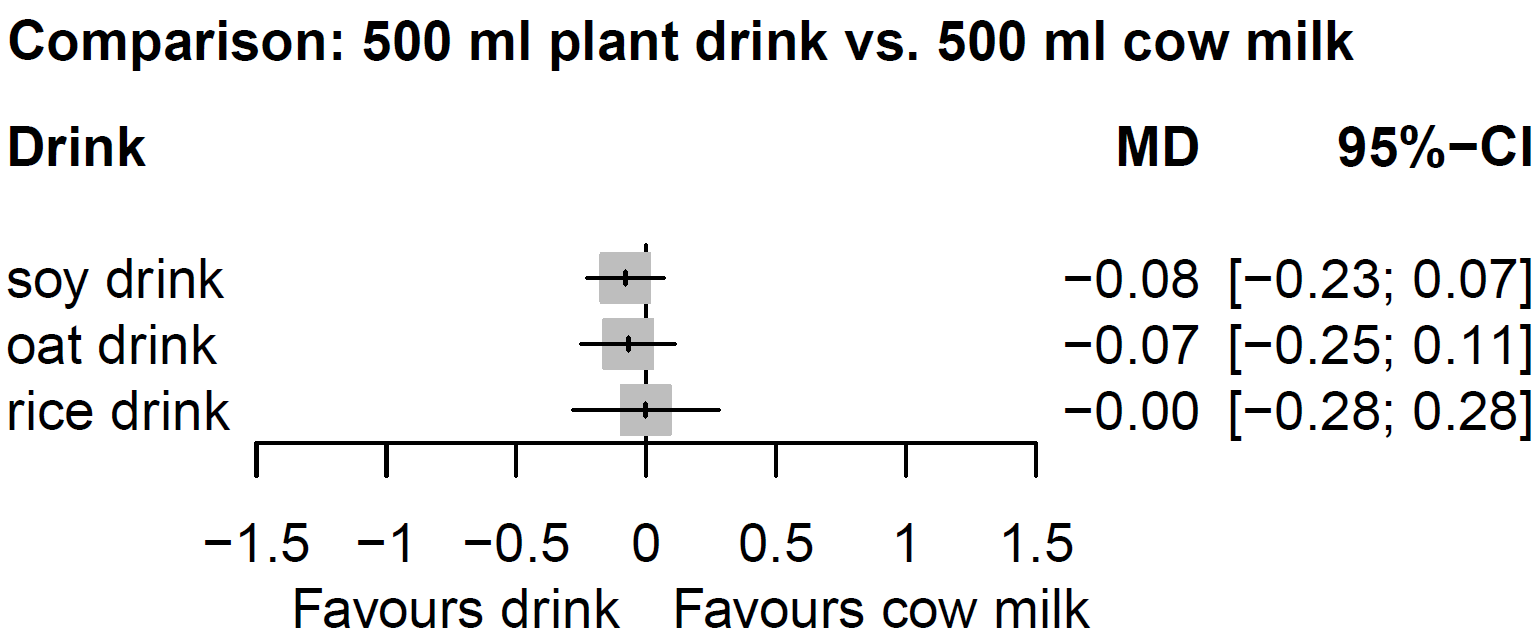


95%-CI 95% confidence interval, MD mean difference

MD mean differences established from network meta-analysis (NMA) harmonized doses (500 ml/d)

# Supplemental Figure 34. Sensitivity analysis pairwise meta-analysis with change scores only

**Outcome: systolic blood pressure (mmHg)**


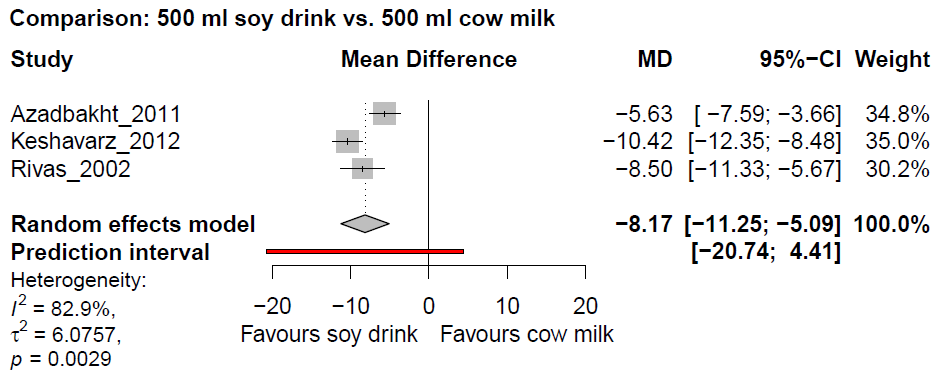


95%-CI 95% confidence interval, MD mean difference

MD mean differences established from pairwise meta-analysis harmonized doses (500 ml/d)

# Supplemental Figure 35. Sensitivity analysis pairwise meta-analysis with change scores only

**Outcome: diastolic blood pressure (mmHg)**


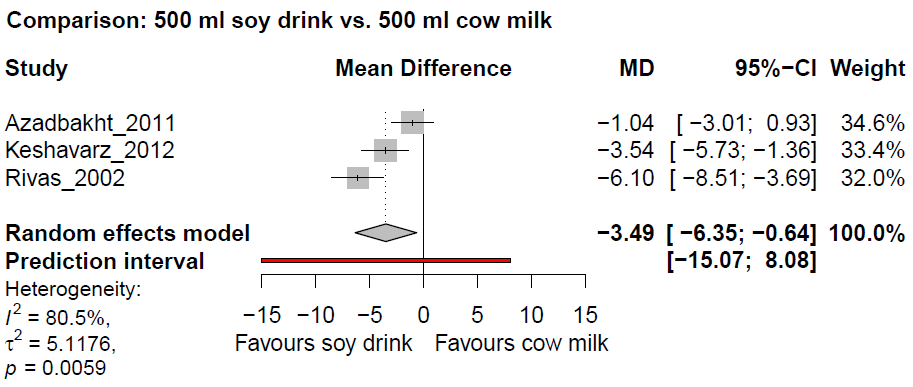


95%-CI 95% confidence interval, MD mean difference

MD mean differences established from pairwise meta-analysis harmonized doses (500 ml/d)

# Supplemental Figure 36. Dose-response curve from dose-response network meta-analysis for body weight

**A**


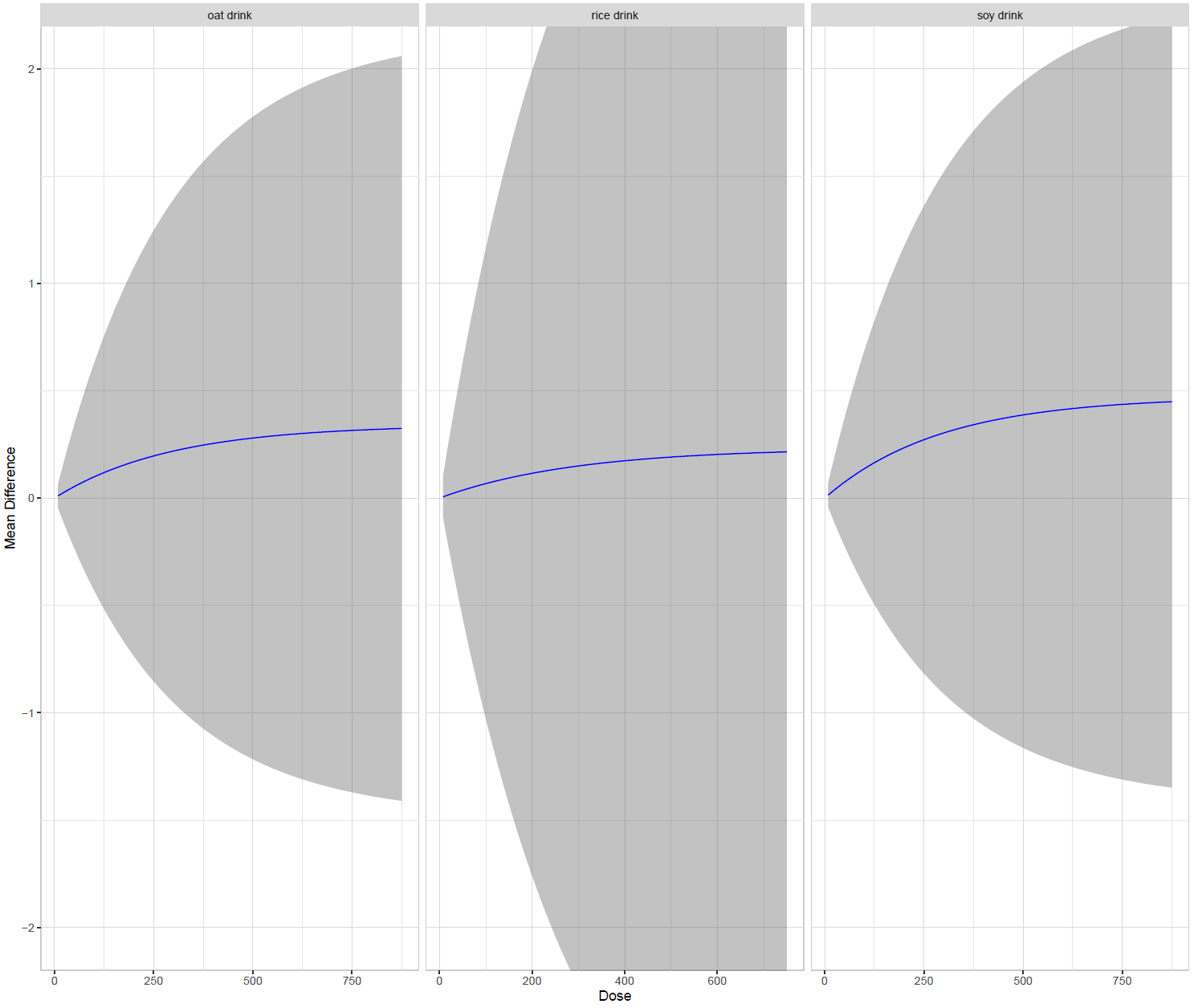


Dose-response plot using post values and change scores. Comparison: plant-drink vs. cow’s milk (reference intervention). Selected dose-response NMA model: Exponential. Heterogeneity: I² = 0,  $\hat{\tau}^{2}$ = 0, Q/df = 0.37**B**


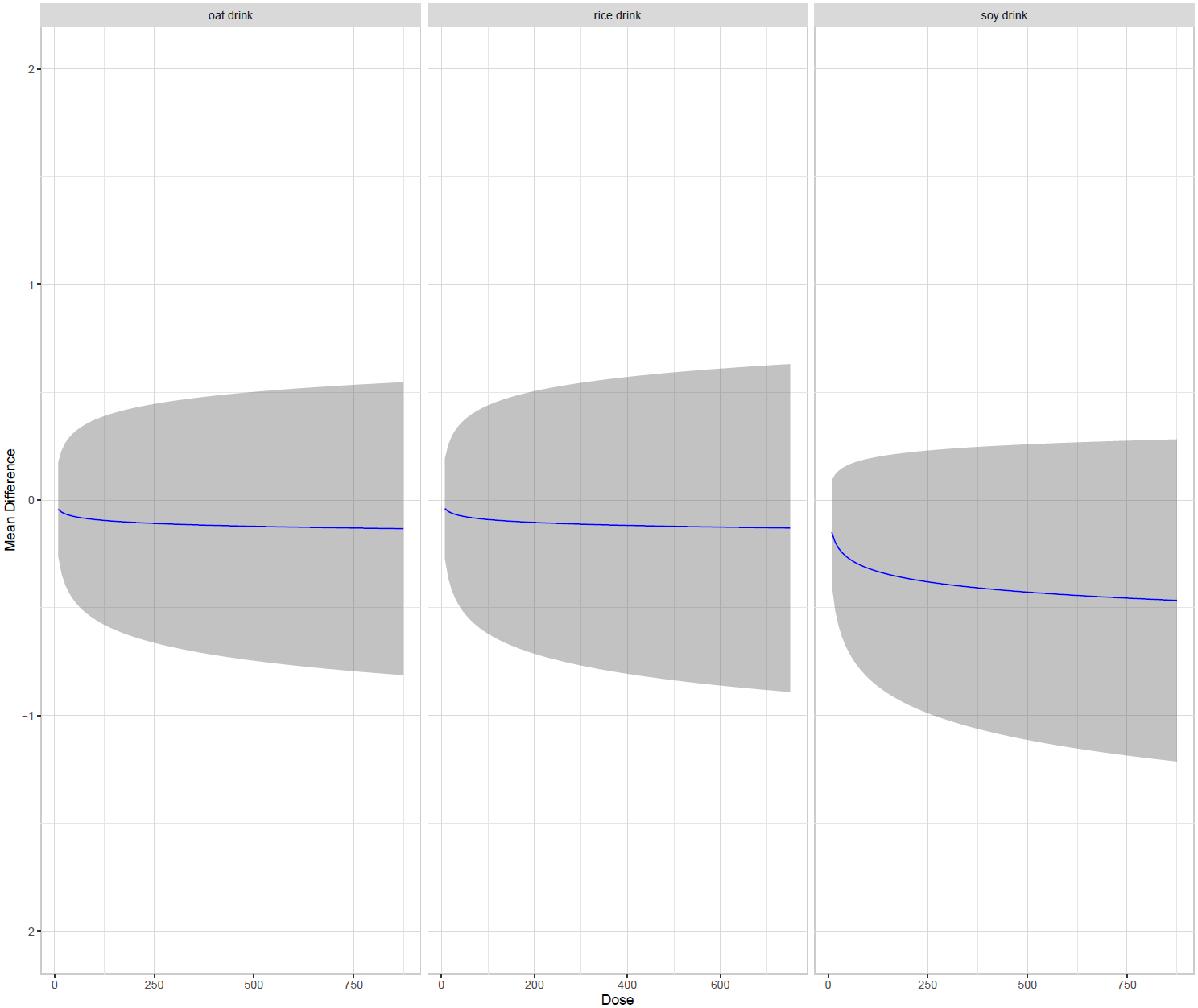


Dose-response plot using change scores only. Comparison: plant-drink vs. cow’s milk (reference intervention). Selected dose-response NMA model: FP1 (p = 0). Heterogeneity: I² = 0.75,  $\hat{\tau}^{2}$ = 0.02, Q/df = 3.94

# Supplemental Figure 37. Dose-response curve from dose-response network meta-analysis for LDL-Cholesterol

**A**


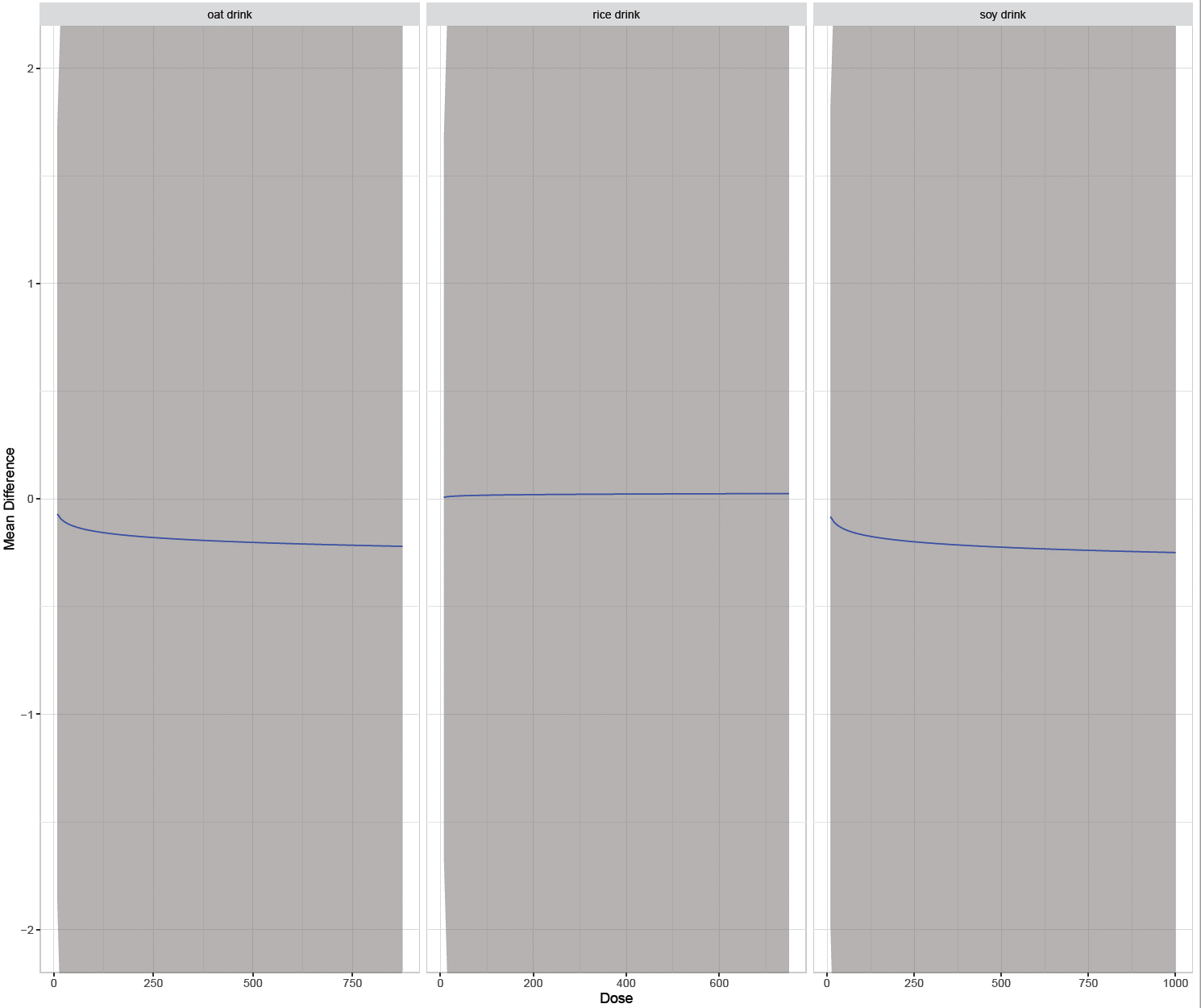


Dose-response plot using post values and change scores. Comparison: plant-drink vs. cow’s milk (reference intervention). Selected dose-response NMA model: FP1 (p = 0). Heterogeneity: I² = 0,  $\hat{\tau}^{2}$ = 0, Q/df = 0.32

**B**


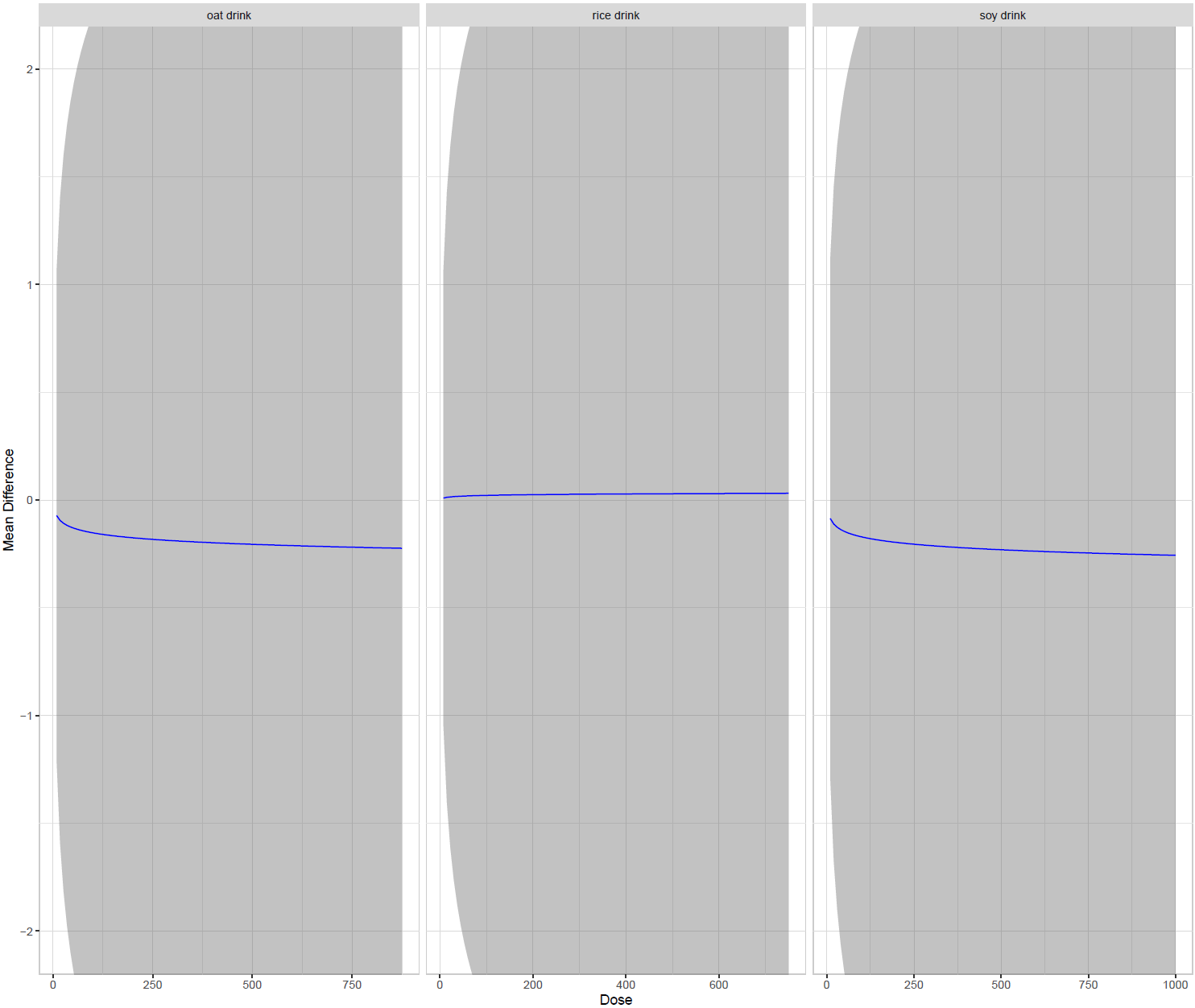


Dose-response plot using change scores only. Comparison: plant-drink vs. cow’s milk (reference intervention). Selected dose-response NMA model: FP1 (p = 0). Heterogeneity: I² = 0.83,  $\hat{\tau}^{2}$ = 0, Q/df = 5.85

# Supplemental Figure 38. Dose-response curve from dose-response network meta-analysis for HDL-Cholesterol

**A**


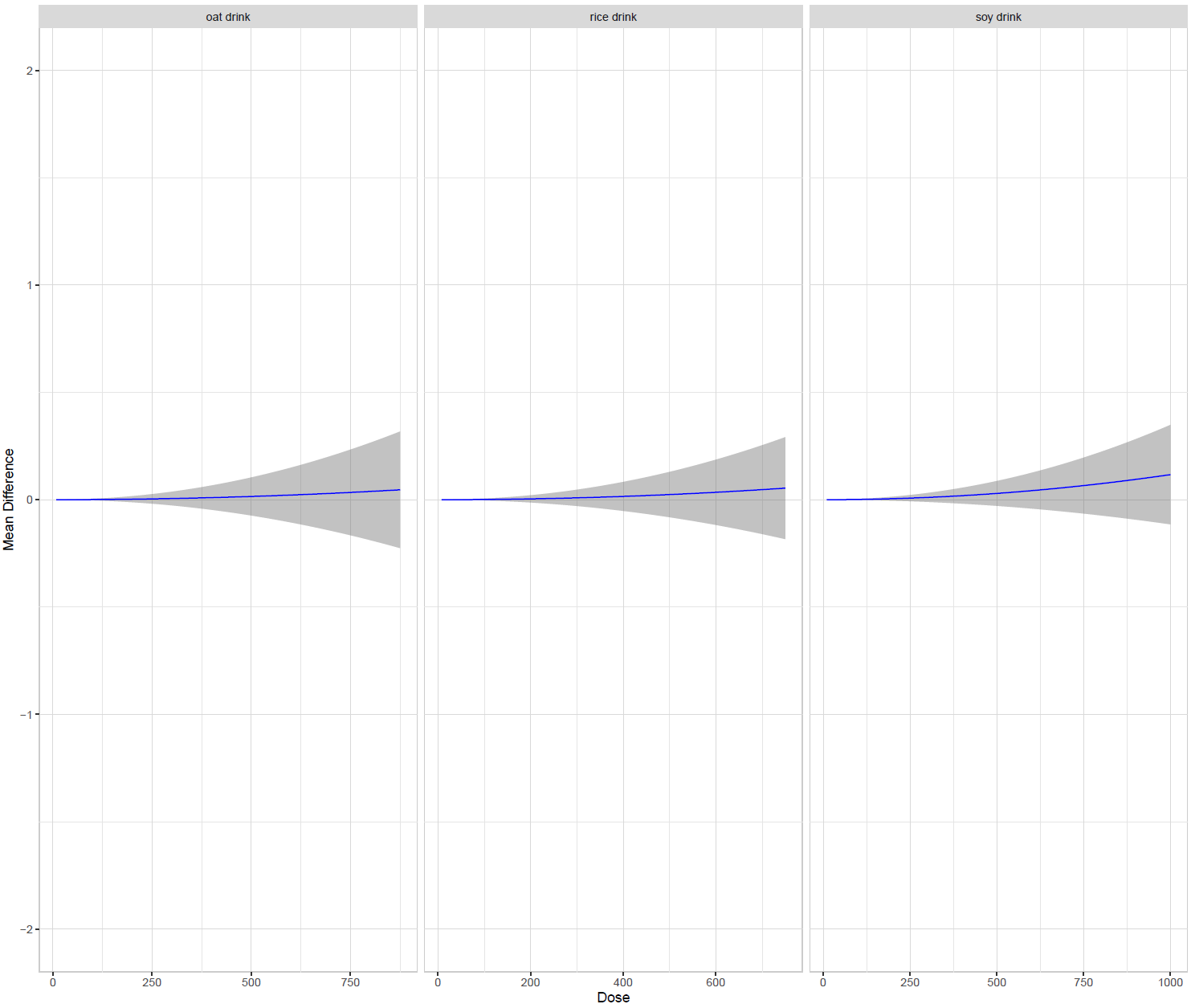


Dose-response plot using post values and change scores. Comparison: plant-drink vs. cow’s milk (reference intervention). Selected dose-response NMA model: Quadratic. Heterogeneity: I² = 0.35,  $\hat{\tau}^{2}$ = 0, Q/df = 1.55

**B**


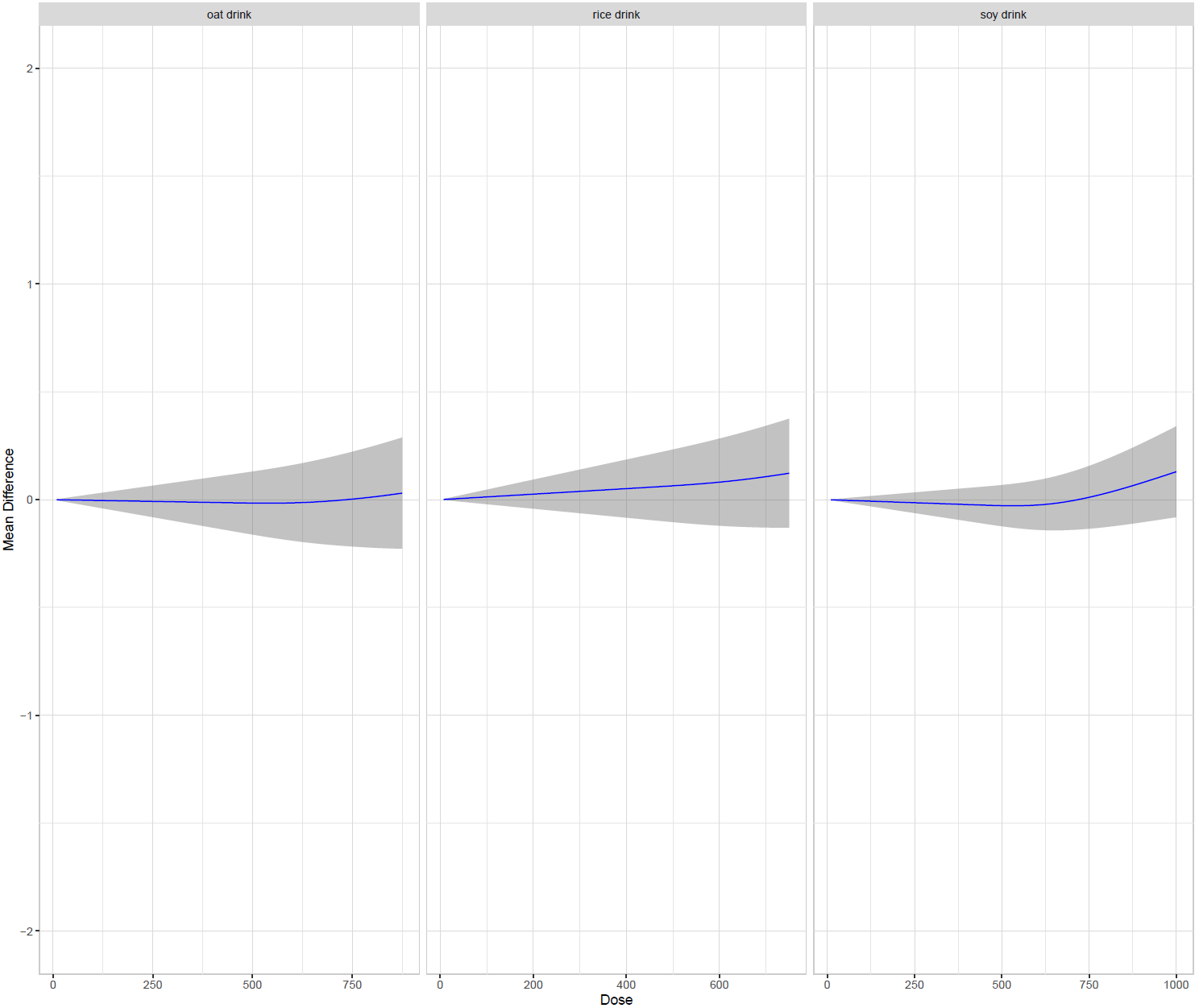


Dose-response plot using change scores only. Comparison: plant-drink vs. cow’s milk (reference intervention). Selected dose-response NMA model: RCS (0.25, 0.5, 1). Heterogeneity: I² = 0.73,  $\hat{\tau}^{2}$ = 0, Q/df = 3.66

# Supplemental Figure 39. Dose-response curve from dose-response network meta-analysis for total cholesterol

**A**


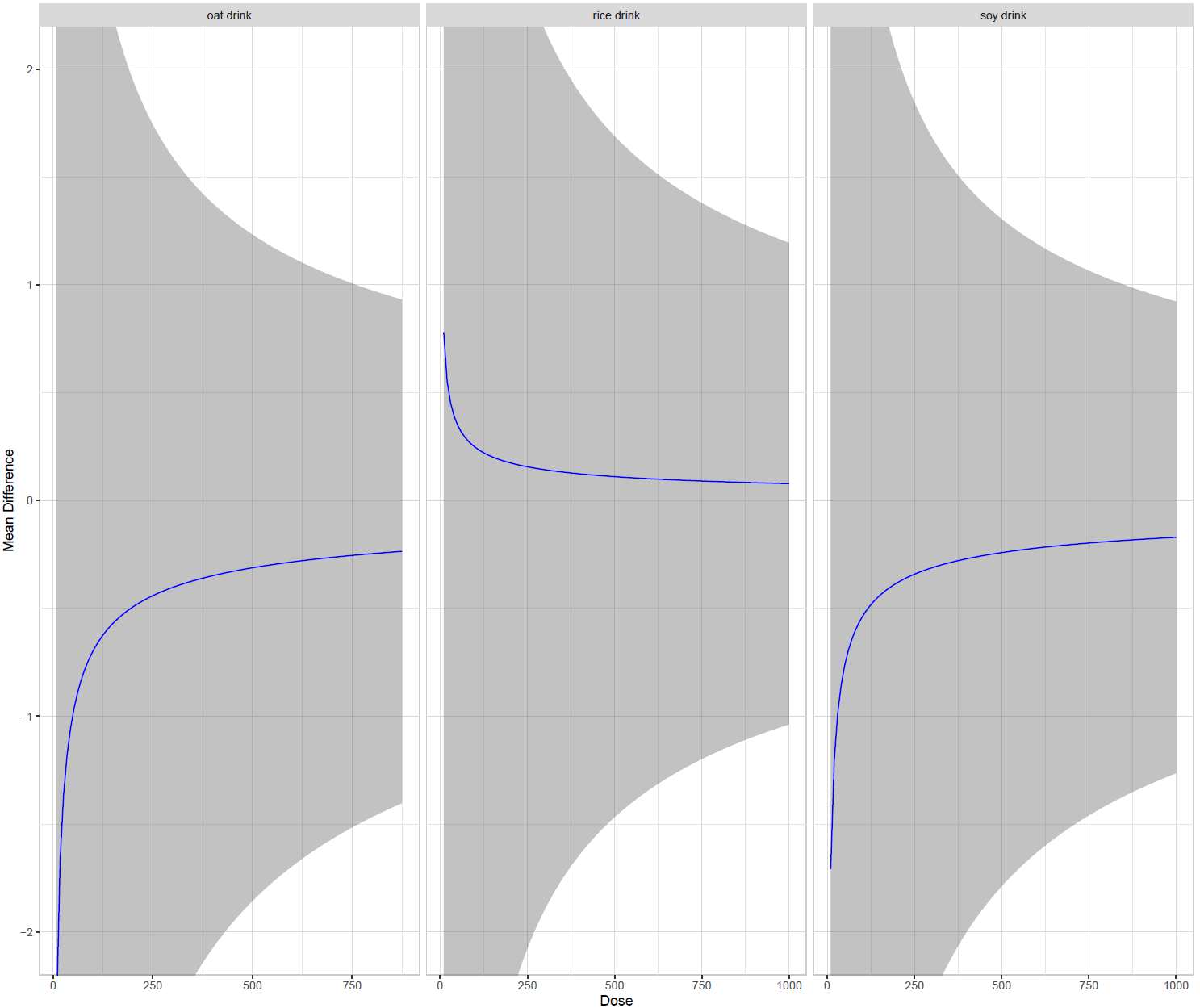


Dose-response plot using post values and change scores. Comparison: plant-drink vs. cow’s milk (reference intervention). Selected dose-response NMA model: FP1 (p = -0.5). Heterogeneity: I² = 0,  $\hat{\tau}^{2}$ = 0, Q/df = 0.42

**B**


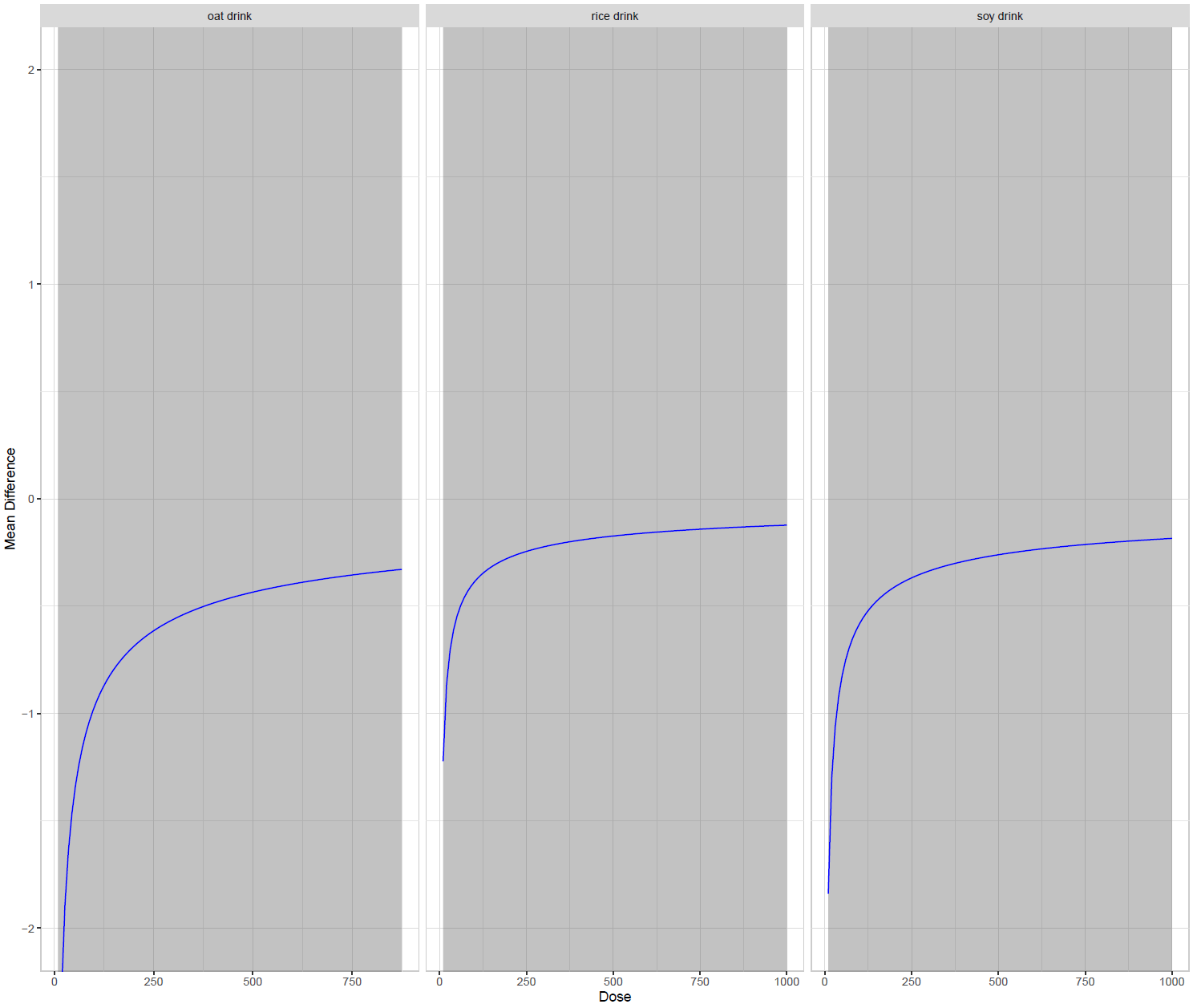


Dose-response plot using change scores only. Comparison: plant-drink vs. cow’s milk (reference intervention). Selected dose-response NMA model: FP1 (p = -0.5). Heterogeneity: I² = 0.25,  $\hat{\tau}^{2}$ = 0.73, Q/df = 1.33

# Supplemental Figure 40. Dose-response curve from dose-response network meta-analysis for triglycerides

**A**


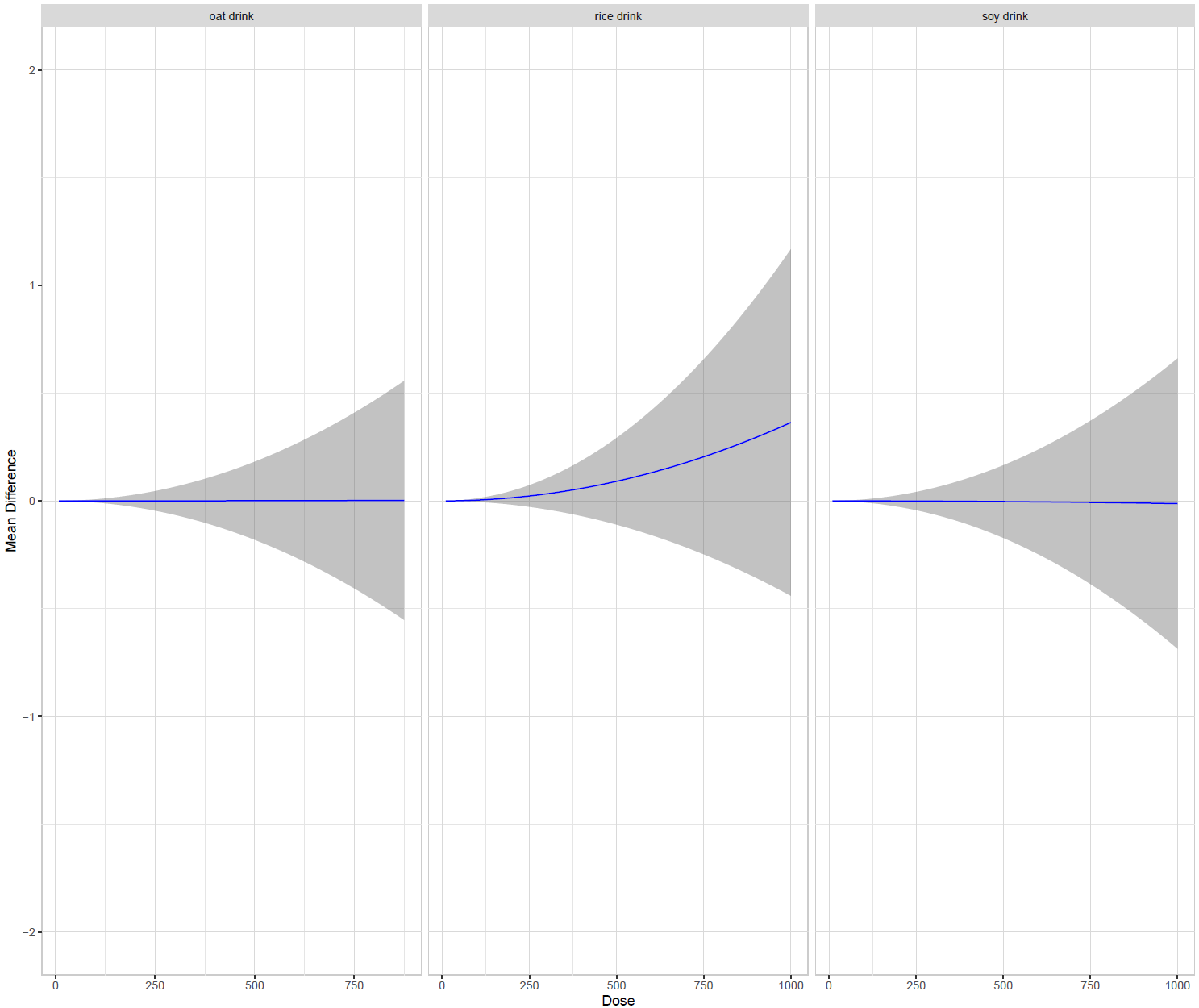


Dose-response plot using post values and change scores. Comparison: plant-drink vs. cow’s milk (reference intervention). Selected dose-response NMA model: FP1 (p = 2). Heterogeneity: I² = 0,  $\hat{\tau}^{2}$ = 0, Q/df = 0.50

**B**


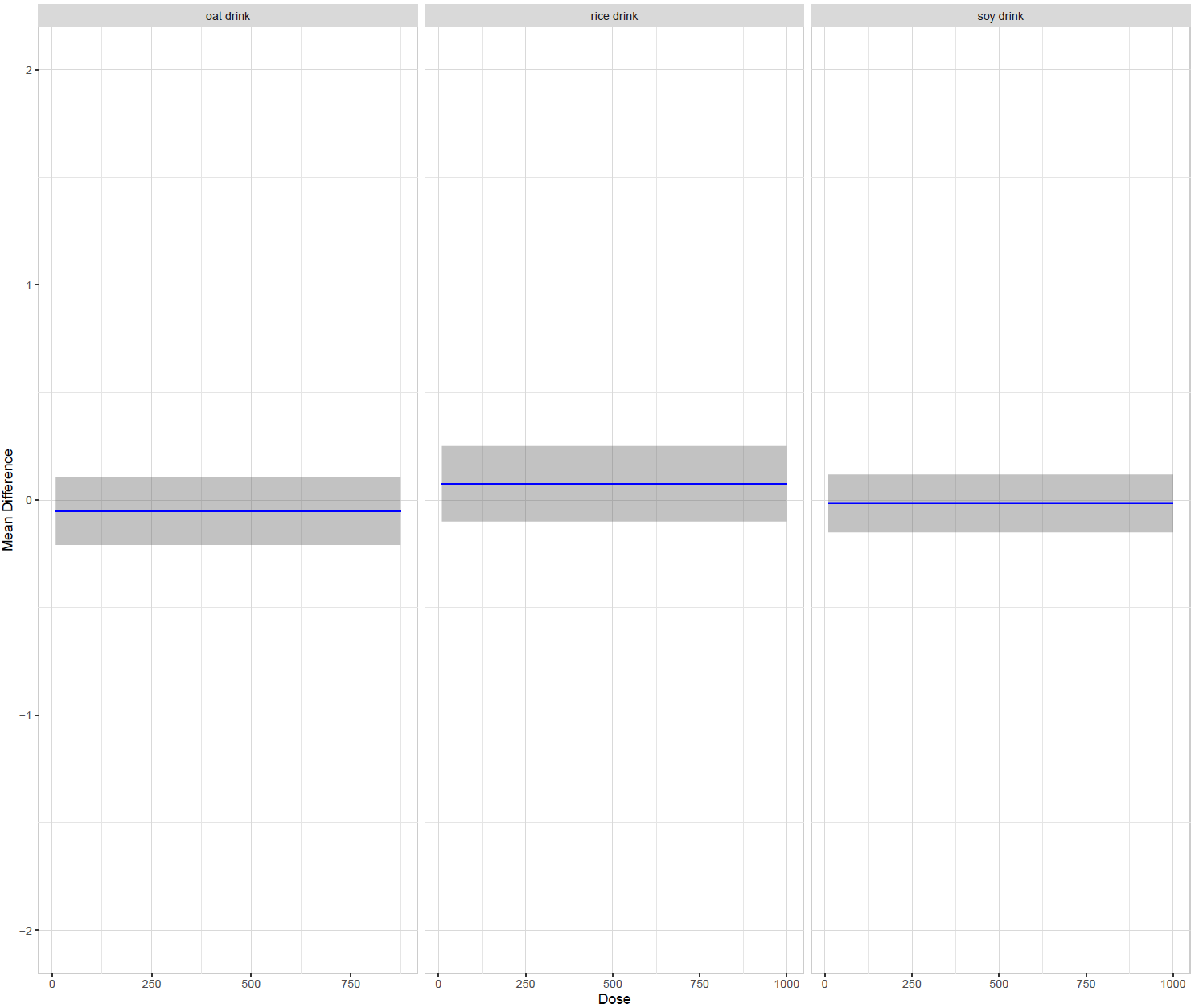


Dose-response plot using change scores only. Comparison: plant-drink vs. cow’s milk (reference intervention). Selected dose-response NMA model: Exponential. Heterogeneity: I² = 0.70,  $\hat{\tau}^{2}$ = 0.005, Q/df = 3.34

# Supplemental Figure 41. Dose-response curve from dose-response network meta-analysis for systolic blood pressure^1^

**A**


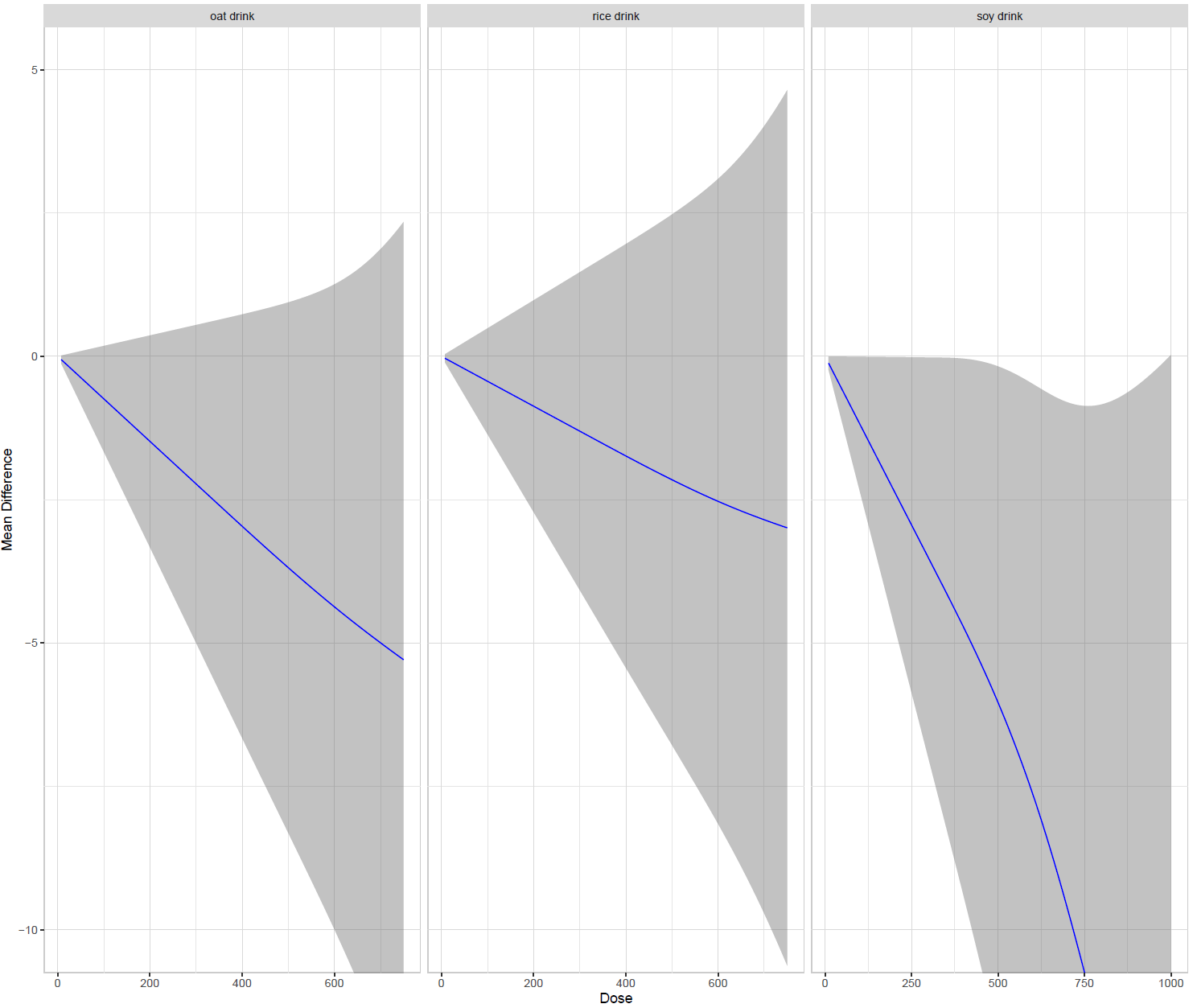


^1^ For systolic blood pressure the network is disconnected in a standard network meta-analysis (NMA).

Dose-response plot using post values and change scores. Comparison: plant-drink vs cow’s milk (reference intervention). Selected dose-response NMA model: RCS (0.1, 0.5, 0.9). Heterogeneity: I² = 0, $\hat{\tau}^{2}$= 0, Q/df = 0.32

**B**


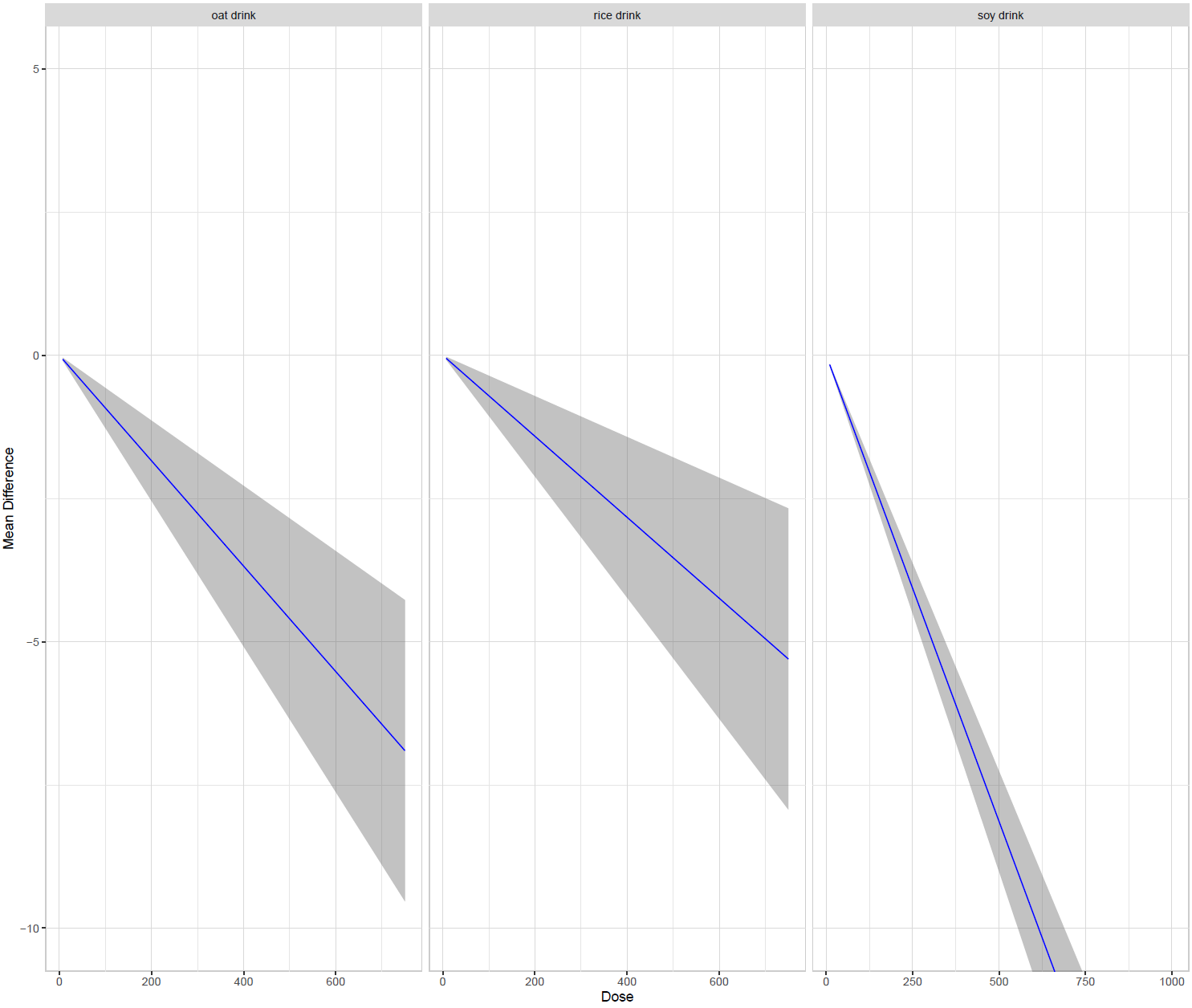
^1^ For systolic blood pressure the network is disconnected in a standard network meta-analysis (NMA).

Dose-response plot using change scores only. Comparison: plant-drink vs. cow’s milk (reference intervention). Selected dose-response NMA model: Linear. Heterogeneity: I² = 0.83,  $\hat{\tau}^{2}$ = 0, Q/df = 5.83

# Supplemental Figure 42. Dose-response curve from dose-response network meta-analysis for diastolic blood pressure^1^

**A**


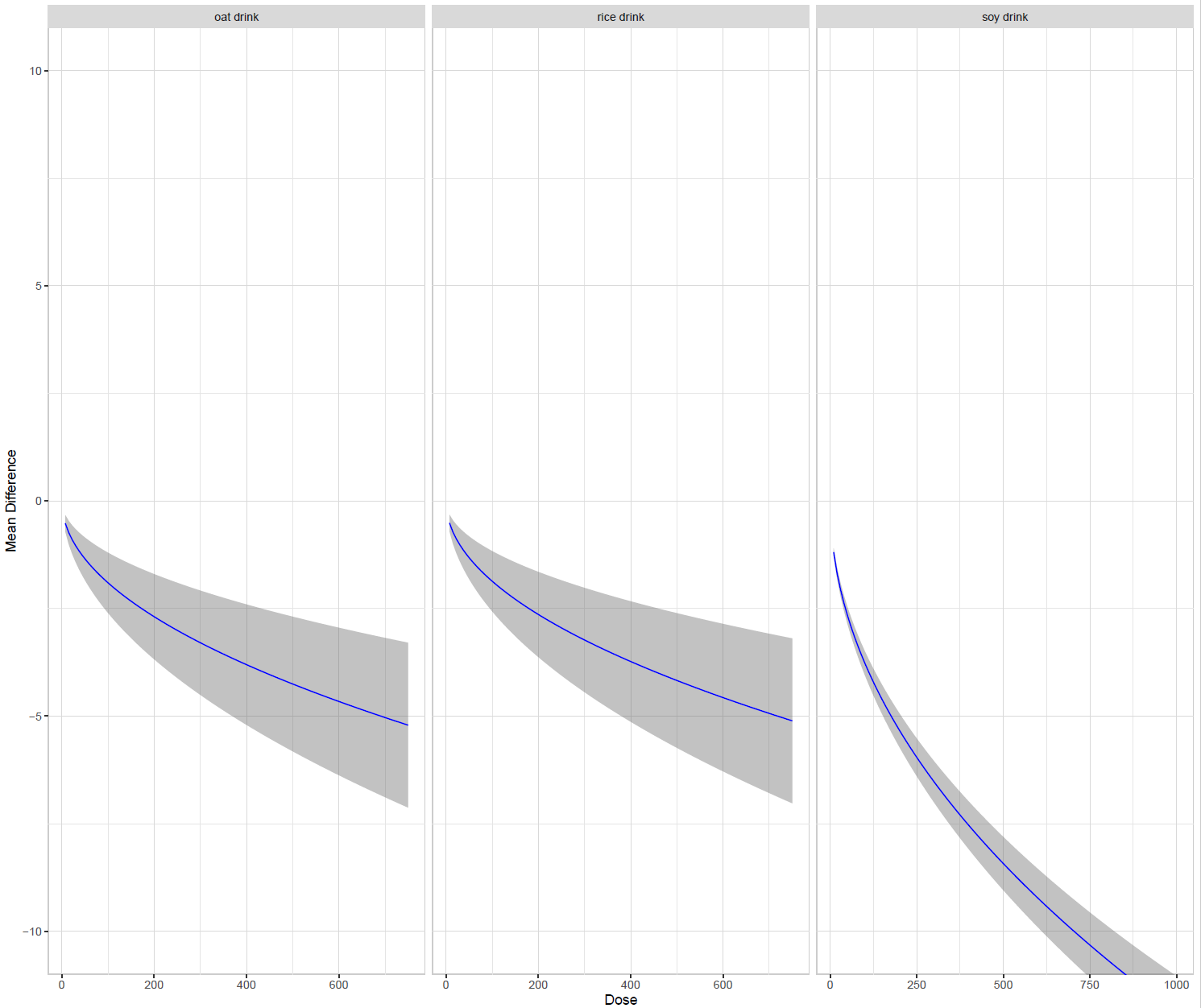


^1^ For diastolic blood pressure the network is disconnected in a common network meta-analysis (NMA).

Dose-response plot using post values and change scores. Comparison: plant-drink vs cow’s milk (reference intervention). Selected dose-response NMA model: FP1 (p = 0.5). Heterogeneity: I² = 0.52, $\hat{\tau}^{2}$= 0.01, Q/df = 2.09

**B**


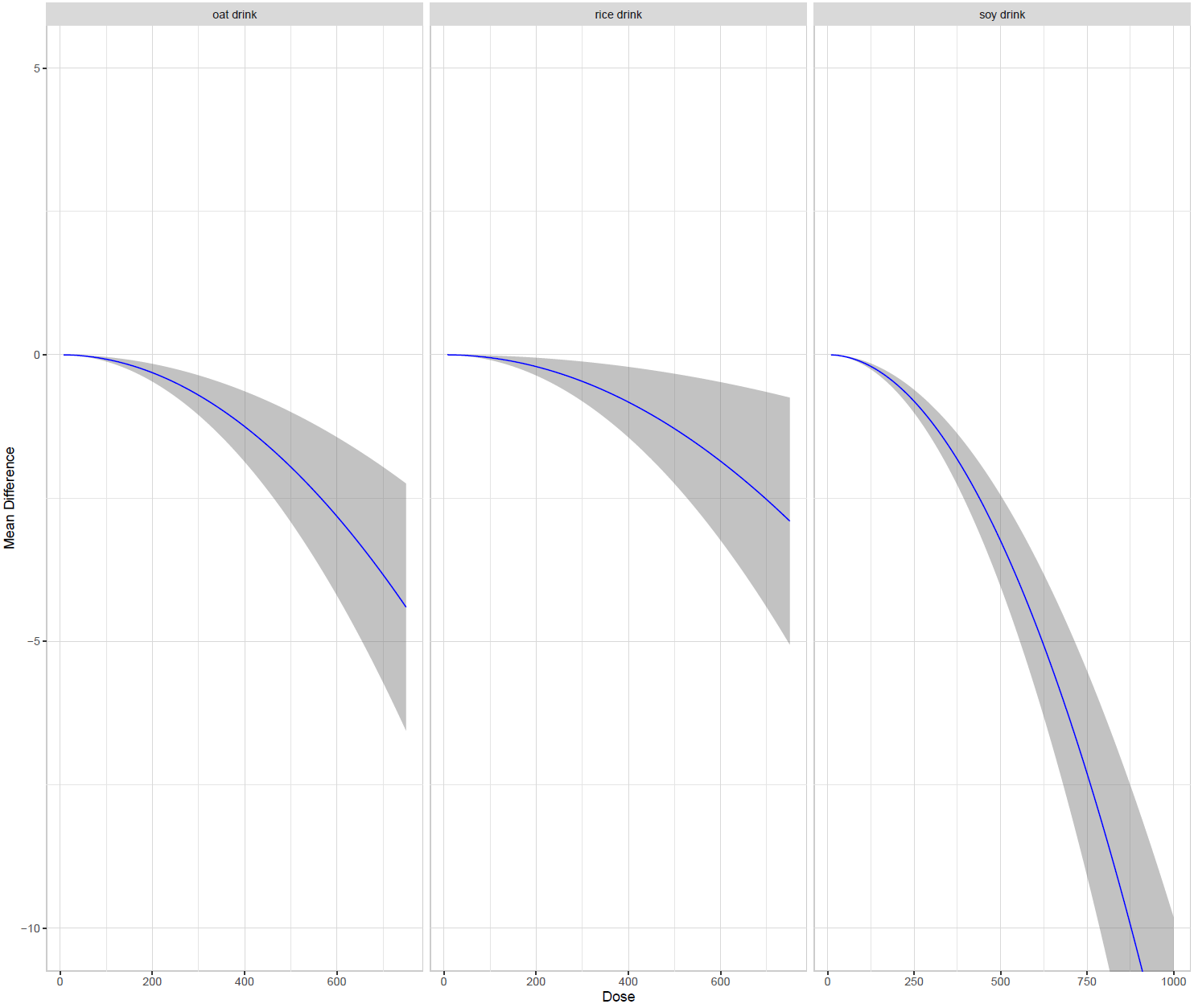


^1^ For diastolic blood pressure the network is disconnected in a common network meta-analysis (NMA).

Dose-response plot using change scores only. Comparison: plant-drink vs. cow’s milk (reference intervention). Selected dose-response NMA model: FP1 (p = 2). Heterogeneity: I² = 0.43,  $\hat{\tau}^{2}$ = 0, Q/df = 1.76

# Supplemental Figure 43. Dose-response curve from dose-response network meta-analysis for fasting blood glucose

**A**


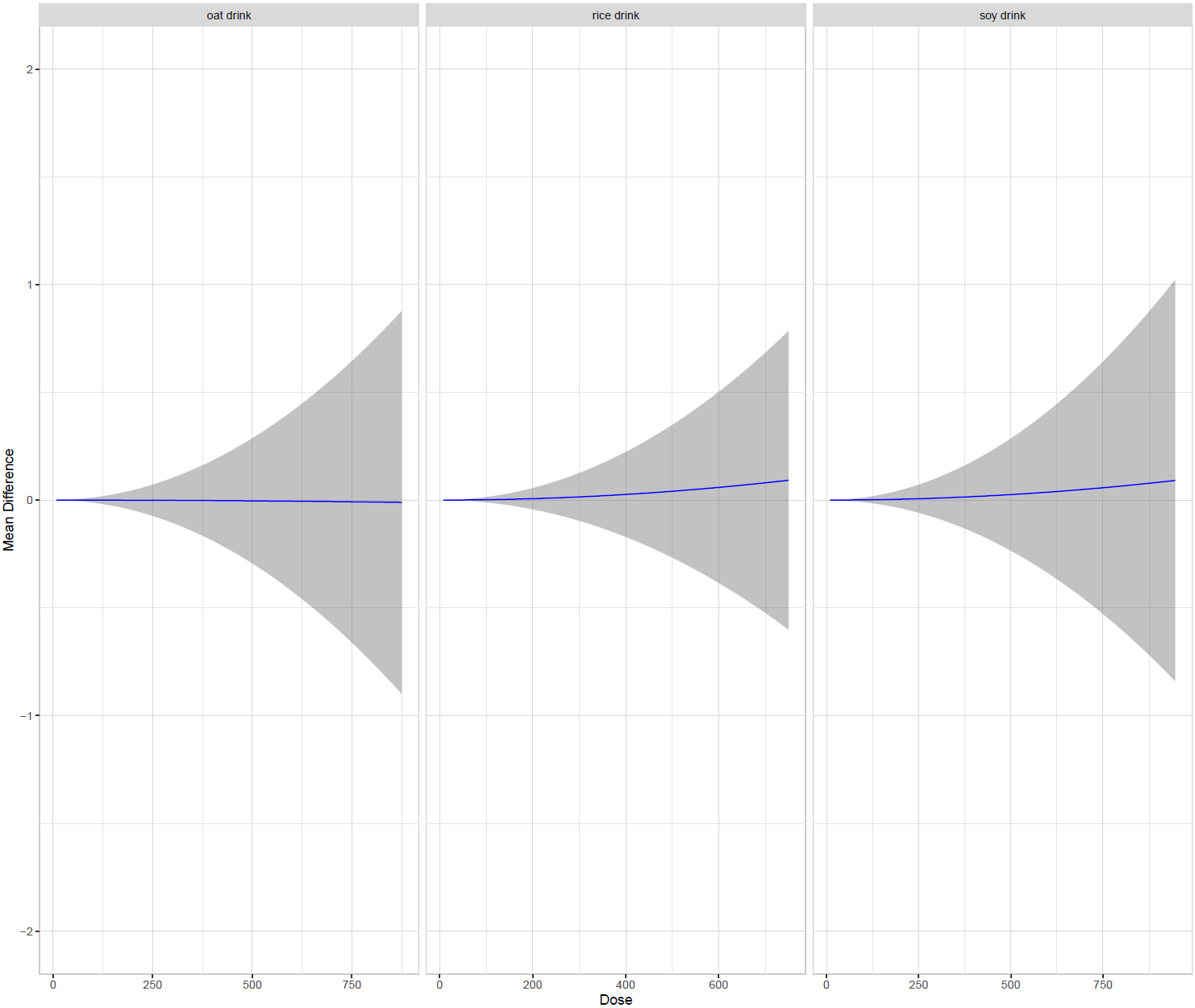


Dose-response plot using post values and change scores. Comparison: plant-drink vs cow’s milk (reference intervention). Selected dose-response NMA model: FP1 (p = 2). Heterogeneity: I² = 0, $\hat{\tau}^{2}$= 0, Q/df = 0.75.

**B**


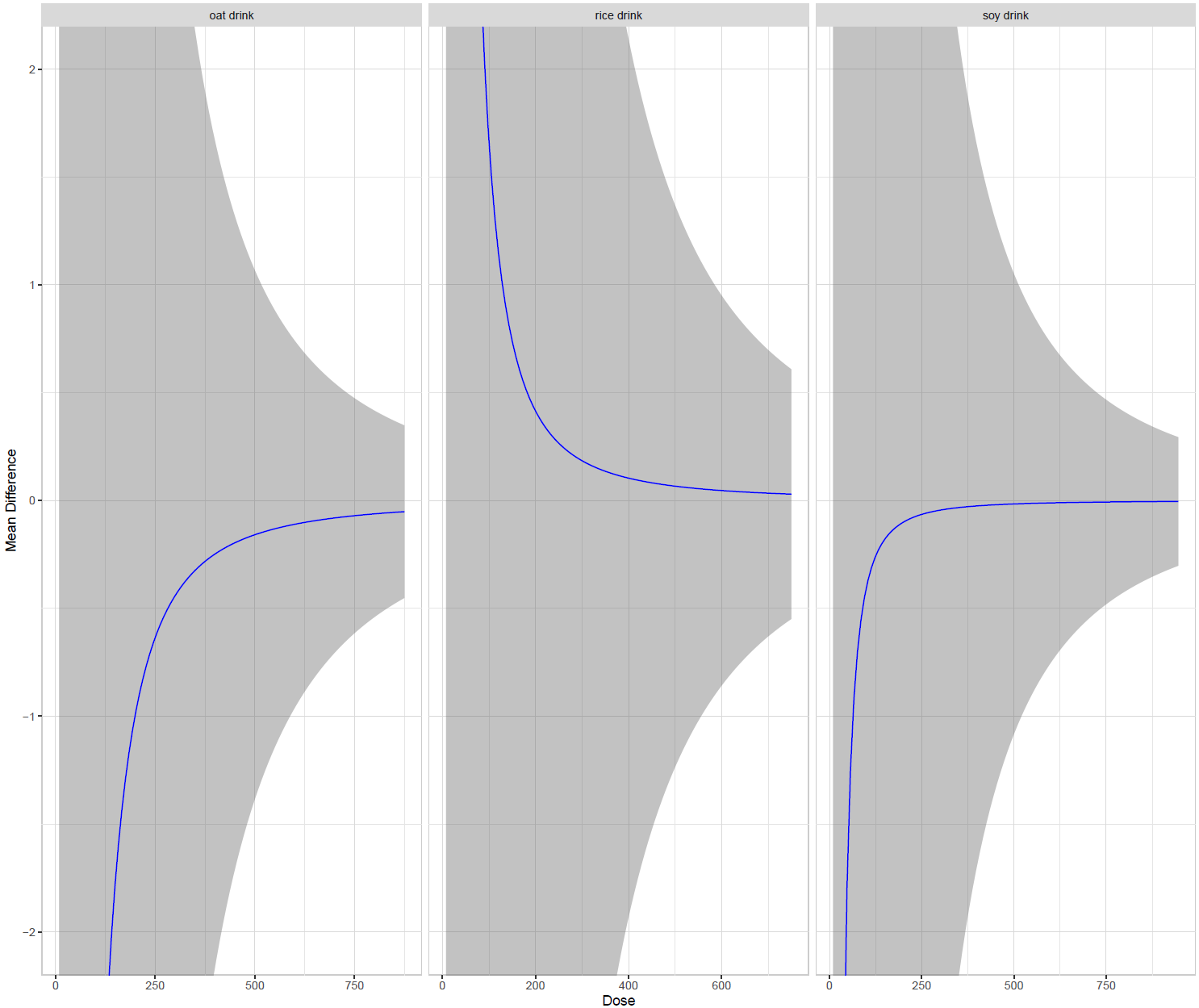


Dose-response plot using post values and change scores. Comparison: plant-drink vs. cow’s milk (reference intervention). Selected dose-response NMA model: FP1 (p = -2). Heterogeneity: I² = 0, $\hat{\tau}^{2}$= 0, Q/df = 0.56.

# Supplemental Appendix 1 Search Strategy

MEDLINE® ALL (via Ovid)

|  | Search |
| --- | --- |
| 1 | milk substitutes/ |
| 2 | exp soy milk/ |
| 3 | (milk* adj3 (noncow or substitut* or alternat* or plant* or vegan or vegeta*)).ti,ab,kf. |
| 4 | ((milk* or alternat* or drink* or beverage*) and (plantbased or plant-based or “plant based”)).ti,ab,kf. |
| 5 | ((soy or soya or soybean or soybeans or oat or oats or almond or almonds or nut or nuts or coconut* or macadamia* or hemp or hempseed* or quinoa or rice or cashew* or corn or maize or millet or spelt or cowpea or pea or peas or peanut* or pecan or pecans or lupin* or hazelnut* or pistachio* or walnut* or sesame* or flax or flaxseed* or sunflower* or teff or amaranth* or pumpkin* or buckwheat or seed* or chickpea* or legume*) adj3 (milk* or beverage* or drink*)).ti,ab,kf. |
| 6 | (soymilk or peamilk).ti,ab,kf. |
| 7 | (nondairy or “non dairy” or non-dairy).ti,ab,kf. |
| 8 | (soy adj3 (food or foods or intake or intakes)).ti,ab,kf. |
| **9** | **or/1-8** |
| 10 | randomized controlled trial.pt. |
| 11 | controlled clinical trial.pt. |
| 12 | randomi?ed.ab. |
| 13 | placebo.ab. |
| 14 | drug therapy.fs. |
| 15 | randomly.ab. |
| 16 | trial.ab. |
| 17 | groups.ab. |
| 18 | or/10-17 |
| 19 | exp animals/ not humans.sh. |
| 20 | 18 not 19 |
| **21** | **9 and 20** |
| 22 | cohort studies/ or follow-up studies/ or longitudinal studies/ or prospective studies/ |
| 23 | (prospective or cohort* or observational or longitudinal or follow-up or cases or (case* and control*) or population-based).ti,ab,kf. |
| 24 | 22 or 23 |
| **25** | **9 and 24** |
| **26** | **21 or 25** |

Cochrane CENTRAL(via CRSO)

| 1 | [milk substitutes] |
| --- | --- |
| 2 | [soy milk] |
| 3 | (milk* NEAR/3 (noncow OR substitut* OR alternat* OR plant* OR vegan OR vegeta*)):ti,ab,kw |
| 4 | ((milk* OR alternat* OR drink* OR beverage*) AND (plantbased OR plant-based OR "plant based")):ti,ab,kw |
| 5 | ((soy OR soya OR soybean OR soybeans OR oat OR oats OR almond OR almonds OR nut OR nuts OR coconut* OR macadamia* OR hemp OR hempseed* OR quinoa OR rice OR cashew* OR corn OR maize OR millet OR spelt OR cowpea OR pea OR peas OR peanut* OR pecan OR pecans OR lupin* OR hazelnut* OR pistachio* OR walnut* OR sesame* OR flax OR flaxseed* OR sunflower* OR teff OR amaranth* OR pumpkin* OR buckwheat OR seed* OR chickpea* OR legume*) NEAR/3 (milk* OR beverage* OR drink*)):ti,ab,kw |
| 6 | (soymilk OR peamilk):ti,ab,kw |
| 7 | (nondairy OR "non dairy" OR non-dairy):ti,ab,kw |
| 8 | (soy NEAR/3 (food OR foods OR intake OR intakes)):ti,ab,kw |
| **9** | **#1 OR #2 OR #3 OR #4 OR #5 OR #6 OR #7 OR #8** |

Web of Science (via Clarivate)

| 1 | TI=("milk substitutes") OR AB=("milk substitutes") |
| --- | --- |
| 2 | TI=((milks OR milk) NEAR/3 (noncow OR substitut* OR alternat* OR plant OR plant-based OR "plant based" OR plantbased OR vegan OR vegeta*)) OR AB=((milk OR milks) NEAR/3 (noncow OR substitut* OR alternat* OR plant OR plant-based OR "plant based" OR plantbased OR vegan OR vegeta*)) |
| 3 | TI=((milk OR milks OR alternat* OR drink OR drinks OR beverage OR beverages) NEAR/3 (plantbased OR plant-based OR "plant based")) OR AB=((milks OR milk OR alternat* OR drink OR drinks OR beverage OR beverages) NEAR/3 (plantbased OR plant-based OR "plant based")) |
| 4 | TI=((soy OR soya OR soybean OR soybeans OR oat OR oats OR almond OR almonds OR nut OR nuts OR coconut OR coconuts OR macadamia OR macadamias OR hemp OR hempseed OR hempseeds OR quinoa OR rice OR cashew OR cashews OR corn OR maize OR millet OR spelt OR cowpea OR pea OR peas OR peanut OR peanuts OR pecan OR pecans OR lupin OR lupins OR hazelnut OR hazelnuts OR pistachio OR pistachios OR walnut OR walnuts OR sesame OR sesames OR flax OR flaxseed OR flaxseeds OR sunflower OR teff OR amaranth OR pumpkin OR pumpkins OR buckwheat OR seed OR seeds OR chickpea OR chickpeas OR legume OR legumes) NEAR/3 (milk OR milks OR beverage OR beverages OR drink OR drinks)) OR AB=((soy OR soya OR soybean OR soybeans OR oat OR oats OR almond OR almonds OR nut OR nuts OR coconut OR coconuts OR macadamia OR macadamias OR hemp OR hempseed OR hempseeds OR quinoa OR rice OR cashew OR cashews OR corn OR maize OR millet OR spelt OR cowpea OR pea OR peas OR peanut OR peanuts OR pecan OR pecans OR lupin OR lupins OR hazelnut OR hazelnuts OR pistachio OR pistachios OR walnut OR walnuts OR sesame OR sesames OR flax OR flaxseed OR flaxseeds OR sunflower OR teff OR amaranth OR pumpkin OR pumpkins OR buckwheat OR seed OR seeds OR chickpea OR chickpeas OR legume OR legumes) NEAR/3 (milk OR milks OR beverage OR beverages OR drink OR drinks)) |
| 5 | TI=(soymilk OR peamilk) OR AB=(soymilk OR peamilk) |
| 6 | TI=(nondairy OR "non dairy" OR non-dairy) OR AB=(nondairy OR "non dairy" OR non-dairy) |
| 7 | TI=(soy NEAR/3 (food OR foods OR intake OR intakes)) OR AB=(soy NEAR/3 (food OR foods OR intake OR intakes)) |
| **8** | **#1 OR #2 OR #3 OR #4 OR #5 OR #6 OR #7** |
| 9 | TI=(randomized or randomised or randomly) OR AB=(randomized or randomised or randomly) |
| 10 | TI=(placebo) OR AB=(placebo) |
| 11 | TI=(trial) OR AB=(trial) |
| 12 | TI=(groups) OR AB=(groups) |
| 13 | #9 OR #10 OR #11 OR #12 |
| **14** | **#8 AND #13** |
| 15 | TI=(prospective OR cohort* OR observational OR longitudinal OR follow-up OR cases OR (case* AND control*) OR population-based) OR AB=(prospective OR cohort* OR observational OR longitudinal OR follow-up OR cases OR (case* AND control*) OR population-based) |
| **16** | **#8 AND #15** |
| **17** | **#14 OR #16** |
| 18 | WC=(Allergy OR Anatomy & Morphology OR Andrology OR Anesthesiology OR Asian Studies OR Behavioral Sciences OR Cardiac & Cardiovascular Systems OR Clinical Neurology OR Critical Care Medicine OR Demography OR Dentistry, Oral Surgery & Medicine OR Dermatology OR Development Studies OR Energy & Fuels OR Ergonomics OR Ethics OR Ethnic Studies OR Gastroenterology & Hepatology OR Genetics & Heredity OR Geriatrics & Gerontology OR Gerontology OR Health Care Sciences & Services OR Hematology OR Humanities, Multidisciplinary OR Immunology OR Infectious Diseases OR Medicine, Research & Experimental OR Multidisciplinary Sciences OR Neurosciences OR Nutrition & Dietetics OR Obstetrics & Gynecology OR Oncology OR Ophthalmology OR Optics OR Ornithology OR Orthopedics OR Pediatrics OR Peripheral Vascular Disease OR Physiology OR Primary Health Care OR Psychology OR Respiratory System OR Rheumatology OR Sport Sciences OR Toxicology OR Urology & Nephrology OR Women's Studies) |
| **19** | **#17 AND #18** |

ClinicalTrials.gov

AREA[InterventionSearch](((milks OR milk) AND (noncow OR substitut OR substitution OR substitutions OR alternative OR alternatives OR plant OR "plant based" OR plantbased OR vegan)) OR ((milk OR milks OR alternative OR alternatives OR drink OR drinks OR beverage OR beverages) AND (plantbased OR plant-based OR "plant based")) OR ((soy OR soya OR soybean OR soybeans OR oat OR oats OR almond OR almonds OR nut OR nuts OR coconut OR coconuts OR macadamia OR macadamias OR hemp OR hempseed OR hempseeds OR quinoa OR rice OR cashew OR cashews OR corn OR maize OR millet OR spelt OR cowpea OR pea OR peas OR peanut OR peanuts OR pecan OR pecans OR lupin OR lupins OR hazelnut OR hazelnuts OR pistachio OR pistachios OR walnut OR walnuts OR sesame OR sesames OR flax OR flaxseed OR flaxseeds OR sunflower OR teff OR amaranth OR pumpkin OR pumpkins OR buckwheat OR seed OR seeds OR chickpea OR chickpeas OR legume OR legumes) AND (milk OR milks OR beverage OR beverages OR drink OR drinks)) OR (soymilk OR peamilk) OR (nondairy OR "non dairy"))

International Clinical Trials Registry Platform (ICTRP)

((milks OR milk) AND (alternat* OR plant OR "plant based" OR plantbased OR vegan)) OR ((milk OR milks OR alternat* OR drink OR drinks OR beverage OR beverages) AND (plantbased OR "plant based")) OR ((soy OR soya OR soybean OR soybeans OR oat OR oats OR almond OR almonds OR nut OR nuts OR coconut OR coconuts OR macadamia OR macadamias OR hemp OR hempseed OR hempseeds OR quinoa OR rice OR cashew OR cashews OR corn OR maize OR millet OR spelt OR cowpea OR pea OR peas OR peanut OR peanuts OR pecan OR pecans OR lupin OR lupins OR hazelnut OR hazelnuts OR pistachio OR pistachios OR walnut OR walnuts OR sesame OR sesames OR flax OR flaxseed OR flaxseeds OR sunflower OR teff OR amaranth OR pumpkin OR pumpkins OR buckwheat OR seed OR seeds OR chickpea OR chickpeas OR legume OR legumes) AND (milk OR milks OR beverage OR beverages OR drink OR drinks)) OR (soymilk OR peamilk) OR (nondairy OR "non dairy")

#

# Supplemental Appendix 2: Additional description and decision criteria for each domain in ROB 2 assessment (for parallel and cross-over trials)

| **Domain 1: Risk of bias arising from randomisation process** | |
| --- | --- |
| **1.1** Was the allocation sequence random? | No information about randomisation method → NI |
| **1.2** Was the allocation sequence concealed until participants were enrolled and assigned to interventions? | Check if allocation was concealed (e.g., by using envelopes or a central or external enrolment service).  If appropriate allocation concealment can be assumed → PY/Y  If no information about allocation concealment is reported → NI |
| **1.3** Did baseline differences between intervention groups suggest a problem with the randomisation process? | Check group sizes.  Look for imbalances for key variables such as age, gender, health status, baseline values of outcomes.  Baseline tables:  If p-values are given, check for significant differences in baseline characteristics between intervention groups.  If p-values are not given, check for large/obvious baseline imbalances between intervention groups.  Text: If small or no significant imbalances are reported → PN/N |

| **Domain S (cross-over trials only): Risk of bias arising from randomisation process** | |
| --- | --- |
| **S.1** Was the number of participants allocated to each of the two sequences equal or nearly equal? | Check the number of participants assigned to each group/ intervention sequence. |
| **S.2** If N/PN/NI to S.1: Were period effects accounted for in the analysis? | Check if the authors made any attempt to account for period effects in the analysis, e.g., by inclusion of intervention by time period interactions. |
| **S.3** Was there sufficient time for any carryover effects to have disappeared before outcome assessment in the second period? | A washout period of 4 weeks was considered to be adequate to diminish any carryover effects in the context of our study.  Check if the wash out period was 4 weeks.  If yes → Y/PY  If no → N/PN |

| **Domain 2: Risk of bias due to deviations from the intended interventions** | |
| --- | --- |
| **2.1** Were participants aware of their assigned intervention during the trial? | In studies with dietary interventions other than supplementation of vitamins/minerals, blinding is likely not possible due to the nature of the included interventions → Y/PY |
| **2.2** Were carers and people delivering the interventions aware of participants' assigned intervention during the trial? | In studies with dietary interventions other than supplementation of vitamins/minerals, blinding is likely not possible due to the nature of the included interventions → Y/PY |
| **2.3.** If Y/PY/NI to 2.1 or 2.2: Were there deviations from the intended intervention that arose because of the trial context? | Check if   1. additional interventions that were introduced were not consistent with trial protocol 2. failure to implement the protocol interventions as intended was evident   If no reasons or details of deviations from the planned interventions are reported, it is likely that no deviations occurred → PN/N  If reported deviations are expected to arise in usual care, e.g., disliked diet, missed visits, lost interest, difficulty following diet → PN/N |
| **2.4** If Y/PY to 2.3:  Were these deviations likely to have affected the outcome? | Judge whether the above mentioned aspects/deviations had an impact on the outcome. |
| **2.5** If Y/PY/NI to 2.4: Were these deviations from intended intervention balanced between groups? | See guidance |
| **2.6** Was an appropriate analysis used to estimate the effect of assignment to intervention? | If ITT or modified ITT was used → Y/PY  If ITT or modified ITT can be assumed (i.e. number randomised per group = number analysed per group) → Y/PY  If no details of the analysis are reported (i.e. number randomised per group ≠ number analysed per group, with no information about excluded participants) → NI  If per protocol analysis was used (with investigators actively excluding available data, e.g., due to reasons related to compliance) → PN/N |
| **2.7** If N/PN/NI to 2.6: Was there potential for a substantial impact (on the result) of the failure to analyse participants in the group to which they were randomised? | Cut-off: >5% missing per group (excluded or analysed in wrong group) |

| **Domain 3: Risk of bias due to missing outcome data** | |
| --- | --- |
| **3.1** Were data for this outcome available for all, or nearly all, participants randomised? | Note that imputed data should be regarded as missing data, and not considered as ‘outcome data’ in the context of this question.  Cut-off: ≥20% missing data → N/PN  Low RoB: <20% + valid reasons  Some concerns: <20% without valid reasons  However, if valid imputation techniques mentioned → low RoB |
| **3.2** If N/PN/NI to 3.1: Is there evidence that the result was not biased by missing outcome data? | Check if   1. (multiple) imputation was used 2. Sensitivity analyses were conducted 3. Reasons were given |
| **3.3** If N/PN to 3.2: Could missingness in the outcome depend on its true value?  **3.4** If Y/PY/NI to 3.3: Is it likely that missingness in the outcome depended on its true value? | High risk: > 20%  However, if:   - valid imputation techniques mentioned → low RoB - no imputation techniques are used, but valid reasons are mentioned for both groups and are (nearly) equally distributed across groups, we will not assume high RoB |
|  | |
| **Domain 4: Risk of bias in measurement of the outcome** | |
| **4.1** Was the method of measuring the outcome inappropriate? | - Check if self-reported outcomes were validated by a second source (register, medical records, second questionnaire). |
| **4.2** Could measurement or ascertainment of the outcome have differed between intervention groups? | Check if outcome measurement differed between groups.  If Y/PY → high RoB |
| **4.3** If N/PN/NI to 4.1 and 4.2: Were outcome assessors aware of the intervention received by study participants? | If N/PN → low RoB |
| **4.4** If Y/PY/NI to 4.3: Could assessment of the outcome have been influenced by knowledge of intervention received? | See guidance |
| **4.5** If Y/PY/NI to 4.4: Is it likely that assessment of the outcome was influenced by knowledge of intervention received? | See guidance |

| **Domain 5: Risk of bias in selection of the reported result** | |
| --- | --- |
| **5.1** Were the data that produced this result analysed in accordance with a pre-specified analysis plan that was finalised before unblinded outcome data were available for analysis?  Is the numerical result being assessed likely to have been selected, on the basis of the results, from...  **5.2.** ... multiple eligible outcome measurements (e.g. scales, definitions, time points) within the outcome domain?  **5.3** ... multiple eligible analyses of the data? | Check if   1. protocol or trial registry entry is available 2. information on the pre-specified analysis is given 3. changes to the pre-specific analysis plan were made (check also history of changes of the register entry) 4. deviations were reported in the manuscript.   If no study protocol/registration is available and no deviations are reported in the manuscript. → some concerns  If registry entry is available but no information about the analysis plan exists. → some concerns  If study protocol/registration is present and there is no evidence for differences between protocol and report. → low RoB  If study protocol/registration is present and differences between protocol and report were clearly described and justified in the text.  → low RoB  Cave: Only consider outcome pre-specification information that is dated before the end of trial. |
| **5.4** If CROSS-OVER trial: Is a result based on data from both periods sought, but unavailable on the basis of carryover having been identified? | See guidance |

ITT: intention-to-treat analysis; NI: no information; PN/N: partial no/no; PY/Y: partial yes/yes; RoB: risk of bias

# Supplemental Appendix 3: Additional description and decision criteria for each domain in the GRADE assessment.^a^

| **Estimate** | **Domain** | **Judgement / explanation** |
| --- | --- | --- |
| In order to establish the certainty of evidence for each comparison every direct, indirect and network estimate for all comparisons in a network must be evaluated. The guidance is adapted according to Brignardello-Petersen et al. and Izcovich et al. (235, 236). | | |
| **Direct estimate** | **Risk of bias** | *Check proportion of included studies (and participants and events) with some concerns or high RoB.*  Don’t downgrade:  More than 2/3 of the studies (and their contributing weight) are rated with low RoB.  *and*  No cohort study is rated with high RoB.  Downgrade by 1 level:  Less than 2/3 of the studies are rated with low RoB.  *and*  Less than 2/3 of the studies are rated with (very) high RoB, or more than 2/3 of the studies (and their contributing weight) are rated with a high RoB, **but** the sensitivity analysis, excluding studies with a (very) high risk of bias, was robust.  Downgrade by 2 levels:  More than 2/3 of the studies (and their contributing weight) are rated with a high RoB.  *and*  The estimate of the sensitivity analysis, excluding studies with a (very) high risk of bias, differs from the main analysis, or there is no sensitivity analysis. |
|  | **Inconsistency** | *Check (i) if 95%CI overlap only minimally or not at all, (ii) if point estimates vary distinctly between studies, (iii) the p-value of statistical test for heterogeneity and I² value.*  Don’t downgrade:  The point estimates indicate a similar direction of effect and the corresponding 95%CI overlap to a high degree.  ***or***  The point estimates show some heterogeneity, but this can be explained by differences between the studies.  Downgrade by 1 level  The point estimates differ distinctly between studies.  *and*  The corresponding 95%CI overlap only minimally or not at all.  *and*  The I² shows a high and significant heterogeneity. |
|  | **Indirectness** | *Check if the included studies represent the PICO question of the review.*  Don’t downgrade:  The intervention/exposure of the included studies as well as the population studied represents the research question of interest and directly measures outcomes of interest.  Downgrade by 1 level:  The population of included studies differs markedly (in biology and/or physiology) from the population of interest and this could have a substantial impact on the magnitude effect.  *or*  The outcome measures are only available as surrogate parameters instead of the outcome intended.  *or*  The duration, intensity or modality of the intervention/exposure differs to an extend that the magnitude of effect could be influenced. |
|  | **Publication bias** | *Not applicable if less than ten studies per comparison*  Don’t downgrade:  The funnel plot shows no asymmetry.  Downgrade by 1 level:  The funnel plot shows a substantial asymmetry. |
|  | **Overall GRADEing of the direct estimate** | The certainty of evidence for the direct estimate can be rated as “high”, “moderate”, “low” or “very low”. |
| **Indirect estimate ^b^** | **Starting point** | The most dominant first order loop for the indirect estimate is formed by two direct estimates / has only one additional node. For example, the indirect evidence of A vs. C is established by A vs. B and B vs. C (2 arms, additional node = B).  If there are more than 1 first order loops the one with the higher number of studies and the lesser inverse variance is chosen.  The lower rating of the two comparisons forming the most dominant first order loop is the starting point for the evaluation of the indirect estimate. |
|  | **Intransitivity** | *Check study characteristics and effect modifiers of the studies forming the indirect estimate.*  Don’t downgrade:  Differences in the direct comparisons that form the indirect estimate are assumed to be differences relating only to the exposures of interest per each arm. There are probably no effect modifiers which lead to reasonable questioning of the credibility of the indirect estimate.  Downgrade by 1 level:  Effect modifiers vary substantially between the two arms that form the indirect estimate and there is a strong assumption that this has an impact on the credibility of the indirect estimate. |
|  | **Overall GRADEing of the direct estimate** | The certainty of evidence for the indirect estimate can be rated as “high”, “moderate”, “low” or “very low”, depending on the starting point. |
| **Network estimate** | **Starting point** | The higher rating of the direct and indirect estimate is the starting point for the assessment of the network estimate. |
|  | **Incoherence** | *Compare the direct and indirect effect estimate.*  Don’t downgrade:  The direct and indirect estimates as well as their corresponding 95% CI are coherent. The p-value for the comparison of the indirect and direct evidence is not significant.  Downgrade by 1 level:  The direct and indirect estimates differ beyond chance and this difference cannot be explained. The p-value for the comparison of the indirect and direct evidence is significant. |
|  | **Imprecision** | *Use thresholds/minimally important difference (MID) and 95%CI as a primary criterion for imprecision rating (minimally contextualized approach).*  *MIDs are as follows:*  *Body Weight: ± 0.5 kg (237)*  *Body Mass Index: ± 0.20 kg (237)*  *Waist Circumference: ± 2 cm (237)*  *Total, LDL-, HDL-Cholesterol, and Triglycerides: ± 1 mmol/L (237, 238)*  *Systolic and diastolic blood pressure: ± 2 mmHg (237, 239)*  *Fasting Plasma Glucose: ± 0.5 mmol/L (237, 240)*  *Consider also optimal information size (OIS): ≥ 400 participants per intervention group.*  Do not downgrade:  The 95%CI does not cross any thresholds.  **If the 95%CI crosses the thresholds:**  Downgrade by 1 level:  The point estimate suggests a trivial or no effect, but the 95%CI crosses the threshold of important benefit or harm.  *or*  The point estimate suggests an important benefit or an important harm, but the 95%CI includes a trivial effect.  *or*  The point estimate suggests an important benefit or an important harm, but the 95%CI includes a large effect.  Downgrade by 2 levels:  The point estimate suggests an important benefit, but the 95%CI crosses the threshold of a trivial effect and important harm.  *or*  The point estimate suggests an important harm, but the 95%CI crosses the threshold of a trivial effect and important benefit. |
|  | **OPTION TO UPGRADE** | **Prerequisite for the option to upgrade:**  There was no downgrading for inconsistency and there was no downgrading for more than 1 levels for RoB. The direct and indirect estimates are coherent and there was no downgrading for imprecision. |
|  | **Dose-response** | Option to upgrade by 1 level:  The prerequisites are met and the analyses show a consistent does-response relationship across and within all studies (>50% of studies must report a consistent dose response effect within the study). |
|  | **Large effect** | Option to upgrade by 1 level:  The prerequisites are met and there is a large effect with a narrow 95%CI.  Option to upgrade by 2 levels:  The prerequisites are met and there is a large effect with a narrow 95%CI. |
| **Overall rating:** | | The overall certainty of evidence for each comparison can be rated as “high”, “moderate”, “low” or “very low”. |

95%CI 95% confidence interval; CHO carbohydrates; GRADE grading of recommendations, assessment, development, and evaluations; PRO protein; RR risk ratio; RoB Risk of bias; ROBINS-E tool Risk of Bias In Non-randomized Studies - of Exposures tool; MID minimally important differences;

^a^ For outcomes where only pairwise analysis was possible, all domains of the direct estimate plus the domain for imprecision were used to assess the certainty of the evidence for these outcomes.

^b^ If the certainty of evidence of the direct estimate is “high” and the direct evidence contributes as much as the indirect evidence there is no need to grade the indirect estimate.

# Supplemental Appendix 4: Additional description of dose-response patterns for all outcomes for dose-response network meta-analysis

**Body weight:** The results for body weight showed a small, dose-dependent increase for all plant drinks compared with cow’s milk, with the curve reaching a plateau at around 400 ml/d. Instead, the analysis using only change scores suggested a slight decrease. Of note, the 95% confidence intervals were wide for both analyses and all estimates remained within ±0.5 kg, indicating no clinically relevant effect.

**Blood lipids:** For LDL-cholesterol, results were imprecise, with no clinically relevant effect of rice drink and small reductions of about −0.2 mmol/L for soy and oat drinks across doses; the sensitivity analysis based only on change scores showed similar patterns. High imprecision and wide confidence intervals were also shown in the analyses for total cholesterol; the curves tended to plateau at higher doses. Estimates for HDL-cholesterol remained close to the null, with narrow confidence intervals and only a slight, non-significant increase in both analyses. For triglycerides, soy and oat drinks indicated effects around zero. Rice drink showed a small, non-significant increase (0.1–0.3 mmol/L); in the change-score analysis, estimates stayed close to zero across all plant-drinks and doses.

**Blood pressure:** The effect estimates for systolic blood pressure decreased with higher doses, reaching clinically relevant effects (with respect to the confidence interval) for soy drink at about 750 ml/d, while the change-score analysis showed a concise, linear and dose-dependent reduction for all plant drinks, with clinically relevant and precise effects at around ≥200 ml/d. This was similar in both analyses for diastolic blood pressure.

**Fasting glucose:** In contrast to the harmonized NMA for fasting glucose, the estimates of the dose-response NMA were close to the null, indicating no relevant effects. However, for both analyses the imprecision was high.

# References

1. Nury E, Stadelmaier J, Morze J, Nagavci B, Grummich K, Schwarzer G, et al. Effects of nutritional intervention strategies in the primary prevention of overweight and obesity in school settings: systematic review and network meta-analysis. BMJ Medicine. 2022;1(1):e000346.

2. IRCT2013030312689N1. effect of soymilk intake on inflammatory markers and oxidative stress. 2013.

3. Manjrekar C, Leelavathi K, Saraswathi A, Sujayalakshmi AN, Katyayani V. Evaluation of the special nutrition programme in Mysore City. Indian J Med Res. 1986;83:404-7.

4. Soczynska I, da Costa BR, O’Connor DL, Jenkins DJA, Birken CS, Keown-Stoneman CDG, et al. Plant-Based Milk Consumption and Growth in Children 1–10 Years of Age. The Journal of Nutrition. 2024;154(3):985-93.

5. Xu X, Kabir A, Barr ML, Schutte AE. Different Types of Long-Term Milk Consumption and Mortality in Adults with Cardiovascular Disease: A Population-Based Study in 7236 Australian Adults over 8.4 Years. Nutrients [Internet]. 2022; 14(3).

6. Akamine Y, Millman JF, Uema T, Okamoto S, Yonamine M, Uehara M, et al. Fermented brown rice beverage distinctively modulates the gut microbiota in Okinawans with metabolic syndrome: A randomized controlled trial. Nutrition Research. 2022;103:68-81.

7. Bijeh N, Mohammadnia-Ahmadi M, Hooshamnd-Moghadam B, Eskandari M, Golestani F. Effects of Soy Milk in Conjunction With Resistance Training on Physical Performance and Skeletal Muscle Regulatory Markers in Older Men. Biological Research For Nursing. 2022;24(3):294-307.

8. Cohen T HT, Loiselle SE, et al. A family-centered lifestyle intervention focused on milk and alternatives reduces adiposity in 6-to 8-year-old overweight and obese children compared to control: Results at 6 months from a RCT. Exp Biol. 2014;28(S1).

9. Cohen TR, Hazell TJ, Loiselle S, Kasvis P, Vanstone CA, Kim N, et al. 103: A Family-Centered Lifestyle Intervention Focused On Milk and Alternatives Reduces Adiposity in Six to Eight Y Old Overweight and Obese Children Compared to Control: Results at Six Months From a RCT. Paediatrics & Child Health. 2014;19(6):e72-e.

10. Cohen TR, Hazell TJ, Vanstone CA, Plourde H, Rodd CJ, Weiler HA. A family-centered lifestyle intervention to improve body composition and bone mass in overweight and obese children 6 through 8 years: a randomized controlled trial study protocol. BMC Public Health. 2013;13(1):383.

11. Cohen TR, Hazell TJ, Vanstone CA, Rodd C, Weiler HA. Changes in lean mass and bone parameters in obese children participating in a family-centered lifestyle intervention: results from a 1-year RCT. The FASEB Journal. 2017;31(S1):456.8-.8.

12. Cohen TR, Hazell TJ, Vanstone CA, Rodd C, Weiler HA. Changes in eating behavior and plasma leptin in children with obesity participating in a family-centered lifestyle intervention. Appetite. 2018;125:81-9.

13. Cohen TR, Hazell TJ, Vanstone CA, Rodd C, Weiler HA. A family-centered lifestyle intervention for obese six- to eight-year-old children: Results from a one-year randomized controlled trial conducted in Montreal, Canada. Canadian Journal of Public Health. 2016;107(4):e453-e60.

14. CTRI/2013/12/004189. Evaluation of blood cholesterol lowering effect of a novel functional fermented betaglucan enriched oat bran/milk based drink. 2013.

15. Gardner CD, Newell KA, Cherin R, Haskell WL. The effect of soy protein with or without isoflavones relative to milk protein on plasma lipids in hypercholesterolemic postmenopausal women123. The American Journal of Clinical Nutrition. 2001;73(4):728-35.

16. Hanachi P, S G. Assessment of Soy Phytoestrogens and Exercise on Lipid Profiles and Menopause Symptoms in Menopausal Women. journal Biological Science. 2008;8.

17. Hilpert KF, Kris-Etherton PM, West SG. Lipid Response to a Low-Fat Diet with or without Soy Is Modified by C-Reactive Protein Status in Moderately Hypercholesterolemic Adults1. The Journal of Nutrition. 2005;135(5):1075-9.

18. Hooshmand-Moghadam B, Johne M, Golestani F, Lorenz K, Asad M, Maculewicz E, et al. Effects of soy milk ingestion immediately after resistance training on muscular-related biomarkers in older males: a randomized controlled trial. Biology of Sport. 2023;40(4):1207-17.

19. Ho SC, Guldan GS, Woo J, Yu R, Tse MM, Sham A, et al. A prospective study of the effects of 1-year calcium-fortified soy milk supplementation on dietary calcium intake and bone health in Chinese adolescent girls aged 14 to 16. Osteoporosis International. 2005;16(12):1907-16.

20. Kajimoto O, Furusawa K, Aoi S, Oshima H, Sawada M, Asai N, et al. Effects of soymilk containing soy protein on human serum cholesterol in normal and borderline hypercholesterolemia subjects. Japanese pharmacology and therapeutics. 2006;34(1):119‐34.

21. Kurowska EM, Jordan J, Spence JD, Wetmore S, Piché LA, Radzikowski M, et al. Effects of substituting dietary soybean protein and oil for milk protein and fat in subjects with hypercholesterolemia. Clin Invest Med. 1997;20(3):162-70.

22. Laurin D, Jacques H, Moorjani S, Steinke FH, Gagné C, Brun D, et al. Effects of a soy-protein beverage on plasma lipoproteins in children with familial hypercholesterolemia. The American Journal of Clinical Nutrition. 1991;54(1):98-103.

23. Lukaszuk JM, Luebbers P, Gordon BA. Preliminary Study: Soy Milk as Effective as Skim Milk in Promoting Weight Loss. Journal of the American Dietetic Association. 2007;107(10):1811-4.

24. Maleki Z, Jazayeri S, Eslami O, Shidfar F, Hosseini AF, Agah S, et al. Effect of soy milk consumption on glycemic status, blood pressure, fibrinogen and malondialdehyde in patients with non-alcoholic fatty liver disease: a randomized controlled trial. Complementary Therapies in Medicine. 2019;44:44-50.

25. Maltais ML, Perreault K, Courchesne-Loyer A, Lagacé J-C, Barsalani R, Dionne IJ. Effect of Resistance Training and Various Sources of Protein Supplementation on Body Fat Mass and Metabolic Profile in Sarcopenic Overweight Older Adult Men: A Pilot Study. International Journal of Sport Nutrition and Exercise Metabolism. 2016;26(1):71-7.

26. Maltais ML, Ladouceur JP, Dionne IJ. The Effect of Resistance Training and Different Sources of Postexercise Protein Supplementation on Muscle Mass and Physical Capacity in Sarcopenic Elderly Men. The Journal of Strength & Conditioning Research. 2016;30(6).

27. Martini MC, Dancisak BB, Haggans CJ, Thomas W, Slavin JL. Effects of Soy Intake on Sex Hormone Metabolism in Premenopausal Women. Nutrition and Cancer. 1999;34(2):133-9.

28. Maskarinec G, Ju D, Morimoto Y, Franke AA, Stanczyk FZ. Soy Food Intake and Biomarkers of Breast Cancer Risk: Possible Difference in Asian Women? Nutrition and Cancer. 2017;69(1):146-53.

29. Matthan NR, Jalbert SM, Ausman LM, Kuvin JT, Karas RH, Lichtenstein AH. Effect of soy protein from differently processed products on cardiovascular disease risk factors and vascular endothelial function in hypercholesterolemic subjects23. The American Journal of Clinical Nutrition. 2007;85(4):960-6.

30. Meyer BJ, Larkin TA, Owen AJ, Astheimer LB, Tapsell LC, Howe PRC. Limited Lipid-Lowering Effects of Regular Consumption of Whole Soybean Foods. Annals of Nutrition and Metabolism. 2004;48(2):67-78.

31. Nagata C, Takatsuka N, Inaba S, Kawakami N, Shimizu H. Effect of Soymilk Consumption on Serum Estrogen Concentrations in Premenopausal Japanese Women. JNCI: Journal of the National Cancer Institute. 1998;90(23):1830-5.

32. Nagata C, Takatsuka N, Shimizu H, Hayashi H, Akamatsu T, Murase K. Effect of soymilk consumption on serum estrogen and androgen concentrations in Japanese men. Cancer Epidemiol Biomarkers Prev. 2001;10(3):179-84.

33. NCT01883674. Milk and Milk-produce to Counteract the Loss of Muscle Mass and Function in Exercising Older Adults. 2013.

34. Onuegbu AJ, Olisekodiaka JM, Onibon MO, Adesiyan AA, Igbeneghu CA. Consumption of Soymilk Lowers Atherogenic Lipid Fraction in Healthy Individuals. Journal of Medicinal Food. 2010;14(3):257-60.

35. Poster Sessions. Journal of Diabetes. 2011;3(s1):42-281.

36. The Effect of Increased Soy Protein Intake on Bone Metabolism [Internet]. 2008. Available from: <https://clinicaltrials.gov/study/NCT00661856>.

37. Takatsuka N, Nagata C, Kurisu Y, Inaba S, Kawakami N, Shimizu H. Hypocholesterolemic Effect of Soymilk Supplementation with Usual Diet in Premenopausal Normolipidemic Japanese Women. Preventive Medicine. 2000;31(4):308-14.

38. Törmälä R, Appt S, Clarkson TB, Groop P-H, Rönnback M, Ylikorkala O, et al. Equol production capability is associated with favorable vascular function in postmenopausal women using tibolone; no effect with soy supplementation. Atherosclerosis. 2008;198(1):174-8.

39. Tranche S, Brotons C, Pascual de la Pisa B, Macías R, Hevia E, Marzo-Castillejo M. Impact of a soy drink on climacteric symptoms: an open-label, crossover, randomized clinical trial. Gynecological Endocrinology. 2016;32(6):477-82.

40. JPRN-UMIN000035612. A verification study for the effect of the soybean milk beverage. 2019.

41. Horn LV, Liu K, Gerber J, Schiffer L, Gernhofer N, Greenland P. Oats and Soy in Lipid-Lowering Diets for Women with Hypercholesterolemia: Is There Synergy? Journal of the American Dietetic Association. 2001;101(11):1319-25.

42. Verheus M, van Gils CH, Kreijkamp-Kaspers S, Kok L, Peeters PHM, Grobbee DE, et al. Soy Protein Containing Isoflavones and Mammographic Density in a Randomized Controlled Trial in Postmenopausal Women. Cancer Epidemiology, Biomarkers & Prevention. 2008;17(10):2632-8.

43. Wilkinson SB, Tarnopolsky MA, MacDonald MJ, MacDonald JR, Armstrong D, Phillips SM. Consumption of fluid skim milk promotes greater muscle protein accretion after resistance exercise than does consumption of an isonitrogenous and isoenergetic soy-protein beverage2. The American Journal of Clinical Nutrition. 2007;85(4):1031-40.

44. Wofford MR, Rebholz CM, Reynolds K, Chen J, Chen CS, Myers L, et al. Effect of soy and milk protein supplementation on serum lipid levels: a randomized controlled trial. European Journal of Clinical Nutrition. 2012;66(4):419-25.

45. Zhao X-fH, Lan-ying; Yin, Shi-an; Kastenmayor, Peter; Barclay, Denis. Effect of long term supplementation of mineral-fortified dephytinized soy milk powder on biomarkers of bone turnover in boys aged 12 to 14 years. Chinese journal of preventive medicine. 2003;37(1).

46. Zhu JS, Y; Wang, Y; Wang, L; Zhao. Effect of milk enriched with phytosterol ester on blood cholesterol of patients with hypercholesterolemia: a randomized controlled trial. Journal of hygiene research. 2016;45(5):718-32.

47. Abu Mweis SS, Tayyem RF, Shehadah I, Bawadi HA, Agraib LM, Bani-Hani KE, et al. Food groups and the risk of colorectal cancer: results from a Jordanian case–control study. European Journal of Cancer Prevention. 2015;24(4).

48. Acosta-Navarro JC, Oki AM, Antoniazzi L, Bonfim MAC, Hong V, Gaspar MCdA, et al. Consumption of animal-based and processed food associated with cardiovascular risk factors and subclinical atherosclerosis biomarkers in men. Revista da Associação Médica Brasileira. 2019;65.

49. Akbaraly TN, Ferrie JE, Berr C, Brunner EJ, Head J, Marmot MG, et al. Alternative Healthy Eating Index and mortality over 18 y of follow-up: results from the Whitehall II cohort123. The American Journal of Clinical Nutrition. 2011;94(1):247-53.

50. Akhter M, Inoue M, Kurahashi N, Iwasaki M, Sasazuki S, Tsugane S, et al. Dietary Soy and Isoflavone Intake and Risk of Colorectal Cancer in the Japan Public Health Center–Based Prospective Study. Cancer Epidemiology, Biomarkers & Prevention. 2008;17(8):2128-35.

51. Baglia ML, Zheng W, Li H, Yang G, Gao J, Gao Y-T, et al. The association of soy food consumption with the risk of subtype of breast cancers defined by hormone receptor and HER2 status. International Journal of Cancer. 2016;139(4):742-8.

52. Batis C, Mendez MA, Sotres-Alvarez D, Gordon-Larsen P, Popkin B. Dietary pattern trajectories during 15 years of follow-up and HbA1c, insulin resistance and diabetes prevalence among Chinese adults. J Epidemiol Community Health. 2014;68(8):773-9.

53. Berkey CS, Colditz GA, Rockett HRH, Frazier AL, Willett WC. Dairy Consumption and Female Height Growth: Prospective Cohort Study. Cancer Epidemiology, Biomarkers & Prevention. 2009;18(6):1881-7.

54. Berkey CS, Tamimi RM, Willett WC, Rosner B, Hickey M, Toriola AT, et al. Dietary intake from birth through adolescence in relation to risk of benign breast disease in young women. Breast Cancer Research and Treatment. 2019;177(2):513-25.

55. Budhathoki S, Iwasaki M, Sawada N, Yamaji T, Shimazu T, Sasazuki S, et al. Soy food and isoflavone intake and endometrial cancer risk: the Japan Public Health Center-based prospective study. BJOG: An International Journal of Obstetrics & Gynaecology. 2015;122(3):304-11.

56. Budhathoki S, Joshi AM, Ohnaka K, Yin G, Toyomura K, Kono S, et al. Soy food and isoflavone intake and colorectal cancer risk: The Fukuoka Colorectal Cancer Study. Scandinavian Journal of Gastroenterology. 2011;46(2):165-72.

57. Chan S-g, Ho SC, Kreiger N, Darlington G, So KF, Chong PYY. Dietary Sources and Determinants of Soy Isoflavone Intake among Midlife Chinese Women in Hong Kong12. The Journal of Nutrition. 2007;137(11):2451-5.

58. Chei C-L, Sawada N, Khankari NK, Iwasaki M, Yamaji T, Cai H, et al. Isoflavone and soy food intake and risk of lung cancer in never smokers: report from prospective studies in Japan and China. European Journal of Nutrition. 2023;62(1):125-37.

59. Cordova R, Viallon V, Fontvieille E, Peruchet-Noray L, Jansana A, Wagner K-H, et al. Consumption of ultra-processed foods and risk of multimorbidity of cancer and cardiometabolic diseases: a multinational cohort study. The Lancet Regional Health - Europe. 2023;35:100771.

60. Dabravolskaj J, Veugelers PJ, Amores A, Leatherdale ST, Patte KA, Maximova K. The impact of 12 modifiable lifestyle behaviours on depressive and anxiety symptoms in middle adolescence: prospective analyses of the Canadian longitudinal COMPASS study. International Journal of Behavioral Nutrition and Physical Activity. 2023;20(1):45.

61. Dicken SJ, Dahm CC, Ibsen DB, Olsen A, Tjønneland A, Louati-Hajji M, et al. Food consumption by degree of food processing and risk of type 2 diabetes mellitus: a prospective cohort analysis of the European Prospective Investigation into Cancer and Nutrition (EPIC). The Lancet Regional Health - Europe. 2024;46:101043.

62. Dorjgochoo T, Kallianpur A, Gao Y-T, Cai H, Yang G, Li H, et al. Dietary and lifestyle predictors of age at natural menopause and reproductive span in the Shanghai Women's Health Study. Menopause. 2008;15(5).

63. do Rosario VA, Schoenaker DAJM, Kent K, Weston-Green K, Charlton K. Association between flavonoid intake and risk of hypertension in two cohorts of Australian women: a longitudinal study. European Journal of Nutrition. 2021;60(5):2507-19.

64. Esterle L, Jehan F, Sabatier JP, Garabedian M. Higher Milk Requirements for Bone Mineral Accrual in Adolescent Girls Bearing Specific Caucasian Genotypes in the VDR Promoter1*. Journal of Bone and Mineral Research. 2009;24(8):1389-97.

65. Gao M, Wang H. Frequent milk and soybean consumption are high risks for uterine leiomyoma: A prospective cohort study. Medicine. 2018;97(41).

66. Gates M, Hanning RM, Gates A, Martin ID, Tsuji LJS. Intakes of milk and alternatives among on-reserve First Nations youth in northern and southern Ontario, Canada. Public Health Nutrition. 2013;16(3):515-23.

67. Gonzalez-Soto M, Abdelmagid SA, Ma DWL, El-Sohemy A, Mutch DM. Soy Consumption, but Not Dairy Consumption, Is Inversely Associated with Fatty Acid Desaturase Activity in Young Adults. Nutrients [Internet]. 2021; 13(8).

68. Guthrie JR, Ball M, Murkies A, Dennerstein L. Dietary phytoestrogen intake in mid-life Australian-born women: relationship to health variables. Climacteric. 2000;3(4):254-61.

69. Han D-fM, Jun; Zhou, Xin; Qiu, Hui; Fang, Li; Huang, Shu. A case-control study on the risk of female breast cancer in Wuhan area. Chin J Epidemiol. 2004;25(3):256-60.

70. Han Y, Zong X, Li Y, Colditz GA, Toriola AT. Milk intake and mammographic density in premenopausal women. Breast Cancer Research and Treatment. 2019;174(1):249-55.

71. Hasnain SR, Singer MR, Bradlee ML, Moore LL. Beverage Intake in Early Childhood and Change in Body Fat from Preschool to Adolescence. Childhood Obesity. 2014;10(1):42-9.

72. Hedelin M, Löf M, Andersson TML, Adlercreutz H, Weiderpass E. Dietary Phytoestrogens and the Risk of Ovarian Cancer in the Women's Lifestyle and Health Cohort Study. Cancer Epidemiology, Biomarkers & Prevention. 2011;20(2):308-17.

73. Herber-Gast G-CM, Biesbroek S, Verschuren WMM, Stehouwer CDA, Gansevoort RT, Bakker SJL, et al. Association of dietary protein and dairy intakes and change in renal function: results from the population-based longitudinal Doetinchem cohort study12. The American Journal of Clinical Nutrition. 2016;104(6):1712-9.

74. Hoang T, Cho S, Choi J-Y, Kang D, Shin A. Genetically predicted dietary intake and risks of colorectal cancer: a Mendelian randomisation study. BMC Cancer. 2024;24(1):1153.

75. Ho SC, Chan SG, Yip YB, Chan CSY, Woo JLF, Sham A. Change in bone mineral density and its determinants in pre- and perimenopausal Chinese women: the Hong Kong perimenopausal women osteoporosis study. Osteoporosis International. 2008;19(12):1785-96.

76. Ho SC, Chan SG, Yi Q, Wong E, Leung PC. Soy Intake and the Maintenance of Peak Bone Mass in Hong Kong Chinese Women. Journal of Bone and Mineral Research. 2001;16(7):1363-9.

77. Hsu W-L, Pan W-H, Chien Y-C, Yu KJ, Cheng Y-J, Chen J-Y, et al. Lowered Risk of Nasopharyngeal Carcinoma and Intake of Plant Vitamin, Fresh Fish, Green Tea and Coffee: A Case-Control Study in Taiwan. PLOS ONE. 2012;7(7):e41779.

78. Hwang J, Kim H, Kwon O. Dietary patterns and the risk of diabetes in Korean adults: A cross-sectional and prospective cohort study. Nutrition. 2024;125:112491.

79. Hwang S, Ha AW. Intakes of Dairy and Soy Products and 10-Year Coronary Heart Disease Risk in Korean Adults. Nutrients [Internet]. 2024; 16(17).

80. Ibsen DB, Steur M, Imamura F, Overvad K, Schulze MB, Bendinelli B, et al. Replacement of Red and Processed Meat With Other Food Sources of Protein and the Risk of Type 2 Diabetes in European Populations: The EPIC-InterAct Study. Diabetes Care. 2020;43(11):2660-7.

81. Irastorza I, Ibañez B, Delgado-Sanzonetti L, Maruri N, Vitoria JC. Cow's-Milk–free Diet as a Therapeutic Option in Childhood Chronic Constipation. Journal of Pediatric Gastroenterology and Nutrition. 2010;51(2):171-6.

82. Rupert W. Jakes SWD, Fook-Cheong Ng, Fei Gao, Eng-Hen Ng, Adeline Seow, Hin-Peng Lee, Mimi C. Yu. Mammographic Parenchymal Patterns and Self-reported Soy Intake in Singapore Chinese Women. Cancer Epidemiology, Biomarkers & Prevention. 2002;11:608-13.

83. Jikumaru M, Sato R, Sasaki Y, Hori D, Katayama T, Yabuuchi K, et al. A prospective study for the correlation between cognitive function and soy intake in OITA. Journal of the Neurological Sciences. 2017;381:665-6.

84. Jo G, Oh H, Singh GM, Park D, Shin M-J. Impact of dietary risk factors on cardiometabolic and cancer mortality burden among Korean adults: results from nationally representative repeated cross-sectional surveys 1998–2016. Nutr Res Pract. 2020;14(4):384-400.

85. Jo G, Kwak S, Cho Y, Shin M-J. Abstract MP038: Soft Drink Consumption Increases the Risk of Hypertension in Korean Adults: A Prospective Cohort Study. Circulation. 2017;135(suppl_1):AMP038-AMP.

86. Katagiri R, Sawada N, Goto A, Yamaji T, Iwasaki M, Noda M, et al. Association of soy and fermented soy product intake with total and cause specific mortality: prospective cohort study. BMJ. 2020;368:m34.

87. Khankari NK, Yang JJ, Sawada N, Wen W, Yamaji T, Gao J, et al. Soy Intake and Colorectal Cancer Risk: Results from a Pooled Analysis of Prospective Cohort Studies Conducted in China and Japan. The Journal of Nutrition. 2020;150(9):2442-50.

88. Koh W-P, Wu AH, Wang R, Ang L-W, Heng D, Yuan J-M, et al. Gender-specific Associations Between Soy and Risk of Hip Fracture in the Singapore Chinese Health Study. American Journal of Epidemiology. 2009;170(7):901-9.

89. Kojima N, Kim M, Saito K, Yoshida H, Yoshida Y, Hirano H, et al. Lifestyle-Related Factors Contributing to Decline in Knee Extension Strength among Elderly Women: A Cross-Sectional and Longitudinal Cohort Study. PLOS ONE. 2015;10(7):e0132523.

90. Kojima N, Kim M, Saito K, Yoshida Y, Hirano H, Obuchi S, et al. Effects of Daily Consumption of Soy Products on Basic/Instrumental Activities of Daily Living in Community-Dwelling Japanese Women Aged 75 Years and Older: A 4-Year Cohort Study. Women's Health Reports. 2023;4(1):232-40.

91. Kokubo Y, Iso H, Ishihara J, Okada K, Inoue M, Tsugane S. Association of Dietary Intake of Soy, Beans, and Isoflavones With Risk of Cerebral and Myocardial Infarctions in Japanese Populations. Circulation. 2007;116(22):2553-62.

92. Ko K-P, Kim S-W, Ma SH, Park B, Ahn Y, Lee JW, et al. Dietary intake and breast cancer among carriers and noncarriers of BRCA mutations in the Korean Hereditary Breast Cancer Study123. The American Journal of Clinical Nutrition. 2013;98(6):1493-501.

93. Ko K-P, Park SK, Yang JJ, Ma SH, Gwack J, Shin A, et al. Intake of Soy Products and Other Foods and Gastric Cancer Risk: A Prospective Study. Journal of Epidemiology. 2013;23(5):337-43.

94. Konishi K, Wada K, Yamakawa M, Goto Y, Mizuta F, Koda S, et al. Dietary Soy Intake Is Inversely Associated with Risk of Type 2 Diabetes in Japanese Women but Not in Men. The Journal of Nutrition. 2019;149(7):1208-14.

95. Kurahashi N, Iwasaki M, Sasazuki S, Otani T, Inoue M, Tsugane S, et al. Soy Product and Isoflavone Consumption in Relation to Prostate Cancer in Japanese Men. Cancer Epidemiology, Biomarkers & Prevention. 2007;16(3):538-45.

96. Langsetmo L, Barr SI, Berger C, Kreiger N, Rahme E, Adachi JD, et al. Associations of protein intake and protein source with bone mineral density and fracture risk: A population-based cohort study. The Journal of nutrition, health and aging. 2015;19(8):861-8.

97. Lazarova SV, Jessri M. Associations between dietary patterns and cardiovascular disease risk in Canadian adults: a comparison of partial least squares, reduced rank regression, and the simplified dietary pattern technique. The American Journal of Clinical Nutrition. 2022;116(2):362-77.

98. Lee AH, Su D, Pasalich M, Tang L, Binns CW, Qiu L. Soy and isoflavone intake associated with reduced risk of ovarian cancer in southern Chinese women. Nutrition Research. 2014;34(4):302-7.

99. Lee S-A, Shu X-O, Li H, Yang G, Cai H, Wen W, et al. Adolescent and adult soy food intake and breast cancer risk: results from the Shanghai Women’s Health Study23. The American Journal of Clinical Nutrition. 2009;89(6):1920-6.

100. Leo QJ, Ollberding NJ, Wilkens LR, Kolonel LN, Henderson BE, Le Marchand L, et al. Nutritional factors and non-Hodgkin lymphoma survival in an ethnically diverse population: the Multiethnic Cohort. Eur J Clin Nutr. 2016;70(1):41-6.

101. Leung YY, Jin A, Tan KB, Ang L-W, Yuan J-M, Koh W-P. Food sources of dietary fibre and risk of total knee replacement related to severe osteoarthritis, the Singapore Chinese Health Study. RMD Open. 2021;7(2):e001602.

102. Luo X, Sui J, Yang W, Sun Q, Ma Y, Simon TG, et al. Type 2 Diabetes Prevention Diet and Hepatocellular Carcinoma Risk in US Men and Women. Official journal of the American College of Gastroenterology | ACG. 2019;114(12).

103. Lutsey PL, Steffen LM, Stevens J. Dietary Intake and the Development of the Metabolic Syndrome. Circulation. 2008;117(6):754-61.

104. Maskarinec G, Aylward AG, Erber E, Takata Y, Kolonel LN. Soy intake is related to a lower body mass index in adult women. European Journal of Nutrition. 2008;47(3):138-44.

105. McCreadie RG. Diet, smoking and cardiovascular risk in people with schizophrenia: Descriptive study. British Journal of Psychiatry. 2003;183(6):534-9.

106. Meyer HE, Pedersen JI, LØken EB, Tverdal A. Dietary Factors and the Incidence of Hip Fracture in Middle-aged Norwegians: A Prospective Study. American Journal of Epidemiology. 1997;145(2):117-23.

107. Mínguez-Alarcón L, Afeiche MC, Chiu YH, Vanegas JC, Williams PL, Tanrikut C, et al. Male soy food intake was not associated with in vitro fertilization outcomes among couples attending a fertility center. Andrology. 2015;3(4):702-8.

108. Morimoto Y, Steinbrecher A, Kolonel LN, Maskarinec G. Soy consumption is not protective against diabetes in Hawaii: the Multiethnic Cohort. European Journal of Clinical Nutrition. 2011;65(2):279-82.

109. Murai U, Sawada N, Charvat H, Inoue M, Yasuda N, Yamagishi K, et al. Soy product intake and risk of incident disabling dementia: the JPHC Disabling Dementia Study. European Journal of Nutrition. 2022;61(8):4045-57.

110. Murphy N, Norat T, Ferrari P, Jenab M, Bueno-de-Mesquita B, Skeie G, et al. Consumption of Dairy Products and Colorectal Cancer in the European Prospective Investigation into Cancer and Nutrition (EPIC). PLOS ONE. 2013;8(9):e72715.

111. Nagarathna R, Anand A, Nanda S, Patil SS, Singh A, Rajesh SK, et al. Is the Indian Dietary Pattern Associated WithType 2 Diabetes? A Pan-India Randomized Cluster Sample Study. Annals of Neurosciences. 2020;27(3-4):175-82.

112. Nagata C, Takatsuka N, Shimizu H. Soy and Fish Oil Intake and Mortality in a Japanese Community. American Journal of Epidemiology. 2002;156(9):824-31.

113. Nagata C, Takatsuka N, Kawakami N, Shimizu H. Soy product intake and premenopausal hysterectomy in a follow-up study of Japanese women. European Journal of Clinical Nutrition. 2001;55(9):773-7.

114. Nagata C, Takatsuka N, Kawakami N, Shimizu H. Soy Product Intake and Hot Flashes in Japanese Women: Results from a Community-based Prospective Study. American Journal of Epidemiology. 2001;153(8):790-3.

115. Nagata C, Takatsuka N, Kawakami N, Shimizu H. A prospective cohort study of soy product intake and stomach cancer death. British Journal of Cancer. 2002;87(1):31-6.

116. Nagata C, Takatsuka N, Kurisu Y, Shimizu H. Decreased Serum Total Cholesterol Concentration Is Associated with High Intake of Soy Products in Japanese Men and Women1,2. The Journal of Nutrition. 1998;128(2):209-13.

117. Nagata CT, N.; Norito, K.; Shimizu, H. Soy product intake and premenopausal hysterectomy in a follow up study of Japanese women. JOURNAL OF NUTRITION. 2002;132(3).

118. Nagata C, Wada K, Tamura T, Konishi K, Goto Y, Koda S, et al. Dietary soy and natto intake and cardiovascular disease mortality in Japanese adults: the Takayama study1. The American Journal of Clinical Nutrition. 2017;105(2):426-31.

119. Nakamoto M, Otsuka R, Nishita Y, Tange C, Tomida M, Kato Y, et al. Soy food and isoflavone intake reduces the risk of cognitive impairment in elderly Japanese women. European Journal of Clinical Nutrition. 2018;72(10):1458-62.

120. Nakamoto M, Otsuka R, Tange C, Nishita Y, Tomida M, Imai T, et al. Intake of isoflavones reduces the risk of all-cause mortality in middle-aged Japanese. European Journal of Clinical Nutrition. 2021;75(12):1781-91.

121. Nanri A, Mizoue T, Shimazu T, Ishihara J, Takachi R, Noda M, et al. Dietary patterns and all-cause, cancer, and cardiovascular disease mortality in Japanese men and women: The Japan public health center-based prospective study. PLOS ONE. 2017;12(4):e0174848.

122. Nanri A, Mizoue T, Takahashi Y, Kirii K, Inoue M, Noda M, et al. Soy Product and Isoflavone Intakes Are Associated with a Lower Risk of Type 2 Diabetes in Overweight Japanese Women12. The Journal of Nutrition. 2010;140(3):580-6.

123. Nguyen HN, Miyagawa N, Miura K, Okuda N, Yoshita K, Arai Y, et al. Dietary tofu intake and long-term risk of death from stroke in a general population. Clinical Nutrition. 2018;37(1):182-8.

124. Nozue M, Shimazu T, Charvat H, Mori N, Mutoh M, Sawada N, et al. Fermented soy products intake and risk of cardiovascular disease and total cancer incidence: The Japan Public Health Center-based Prospective study. European Journal of Clinical Nutrition. 2021;75(6):954-68.

125. Nozue M, Shimazu T, Sasazuki S, Charvat H, Mori N, Mutoh M, et al. Fermented Soy Product Intake Is Inversely Associated with the Development of High Blood Pressure: The Japan Public Health Center-Based Prospective Study. The Journal of Nutrition. 2017;147(9):1749-56.

126. Oba S, Nagata C, Shimizu N, Shimizu H, Kametani M, Takeyama N, et al. Soy Product Consumption and the Risk of Colon Cancer: A Prospective Study in Takayama, Japan. Nutrition and Cancer. 2007;57(2):151-7.

127. Ollberding NJ, Lim U, Wilkens LR, Setiawan VW, Shvetsov YB, Henderson BE, et al. Legume, Soy, Tofu, and Isoflavone Intake and Endometrial Cancer Risk in Postmenopausal Women in the Multiethnic Cohort Study. JNCI: Journal of the National Cancer Institute. 2012;104(1):67-76.

128. Ozawa M, Ninomiya T, Ohara T, Doi Y, Uchida K, Shirota T, et al. Dietary patterns and risk of dementia in an elderly Japanese population: the Hisayama Study123. The American Journal of Clinical Nutrition. 2013;97(5):1076-82.

129. Park S-Y, Murphy SP, Wilkens LR, Henderson BE, Kolonel LN. Legume and isoflavone intake and prostate cancer risk: The Multiethnic Cohort Study. International Journal of Cancer. 2008;123(4):927-32.

130. Paul P, Koh W-P, Jin A, Michel A, Waterboer T, Pawlita M, et al. Soy and tea intake on cervical cancer risk: the Singapore Chinese Health Study. Cancer Causes & Control. 2019;30(8):847-57.

131. Rai SK, Wang S, Hu Y, Hu FB, Wang M, Choi HK, et al. Adherence to Healthy and Unhealthy Plant-Based Diets and the Risk of Gout. JAMA Network Open. 2024;7(5):e2411707-e.

132. Sakauchi F, Khan MMH, Mori M, Kubo T, Fujino Y, Suzuki S, et al. Dietary Habits and Risk of Ovarian Cancer Death in a Large-Scale Cohort Study (JACC Study) in Japan. Nutrition and Cancer. 2007;57(2):138-45.

133. Sawada N, Iwasaki M, Yamaji T, Shimazu T, Inoue M, Tsugane S, et al. Soy and isoflavone consumption and subsequent risk of prostate cancer mortality: the Japan Public Health Center-based Prospective Study. International Journal of Epidemiology. 2020;49(5):1553-61.

134. Schepp M, Freuer D, Wawro N, Peters A, Heier M, Teupser D, et al. Association of the habitual dietary intake with the fatty liver index and effect modification by metabotypes in the population-based KORA-Fit study. Lipids in Health and Disease. 2024;23(1):99.

135. Seely S. Diet and coronary arterial disease: a statistical study. International Journal of Cardiology. 1988;20(2):183-92.

136. Seow A, Koh W-P, Wang R, Lee H-P, Yu MC. Reproductive Variables, Soy Intake, and Lung Cancer Risk among Nonsmoking Women in the Singapore Chinese Health Study. Cancer Epidemiology, Biomarkers & Prevention. 2009;18(3):821-7.

137. Seow A, Poh W-T, Teh M, Eng P, Wang Y-T, Tan W-C, et al. Diet, reproductive factors and lung cancer risk among Chinese women in Singapore: Evidence for a protective effect of soy in nonsmokers. International Journal of Cancer. 2002;97(3):365-71.

138. Shannon J, Ray R, Wu C, Nelson Z, Gao DL, Li W, et al. Food and Botanical Groupings and Risk of Breast Cancer: A Case-Control Study in Shanghai, China. Cancer Epidemiology, Biomarkers & Prevention. 2005;14(1):81-90.

139. Shikany JM, Jacobs DR, Lewis CE, Steffen LM, Sternfeld B, Carnethon MR, et al. Associations between food groups, dietary patterns, and cardiorespiratory fitness in the Coronary Artery Risk Development in Young Adults study123. The American Journal of Clinical Nutrition. 2013;98(6):1402-9.

140. Shimizu HN, C.; Takatsuka, N.; Hirokawa, K.; Morita, N. A prospective cohort study of soy product intake and total mortality in a community of Japan. JOURNAL OF NUTRITION. 2002;132.

141. Shin A, Lee J, Lee J, Park MS, Park JW, Park SC, et al. Isoflavone and Soyfood Intake and Colorectal Cancer Risk: A Case-Control Study in Korea. PLOS ONE. 2015;10(11):e0143228.

142. Shirai Y, Sakuma M, Nagasaka Y, Takeda N, Matsui K, Nakamura M. Association between functional foods and cardiometabolic health in a real-life setting: a longitudinal observational study using objective diet records from an electronic purchase system. Food & Function. 2022;13(4):1751-61.

143. Shirai YS, M.; Nakamura, M. Association between soy products intake and low-density lipoprotein cholesterol in a real-life setting: A longitudinal observational study among male workers in Japan. ANNALS OF NUTRITION AND METABOLISM. 2023;79.

144. Shi Z, Zhao Z, Zhu P, An C, Zhang K. Types of milk consumed and risk of essential hypertension: A 2-sample Mendelian randomization analysis. Journal of Dairy Science. 2023;106(7):4516-23.

145. Shi Z, Ganji V. Dietary patterns and cardiovascular disease risk among Chinese adults: a prospective cohort study. European Journal of Clinical Nutrition. 2020;74(12):1725-35.

146. Shi Z, Papier K, Yiengprugsawan V, Kelly M, Seubsman S-a, Sleigh AC. Dietary patterns associated with hypertension risk among adults in Thailand: 8-year findings from the Thai Cohort Study. Public Health Nutrition. 2019;22(2):307-13.

147. Shu XO, Jin F, Dai Q, Wen W, Potter JD, Kushi LH, et al. Soyfood Intake during Adolescence and Subsequent Risk of Breast Cancer among Chinese Women1. Cancer Epidemiology, Biomarkers & Prevention. 2001;10(5):483-8.

148. Silva S, Severo M, Lopes C. Association between calcium intake from different food sources during childhood and cardiometabolic risk on adolescence: The Generation XXI birth cohort. Pediatric Obesity. 2024;19(10):e13158.

149. Smith JD, Hou T, Ludwig DS, Rimm EB, Willett W, Hu FB, et al. Changes in intake of protein foods, carbohydrate amount and quality, and long-term weight change: results from 3 prospective cohorts23. The American Journal of Clinical Nutrition. 2015;101(6):1216-24.

150. Song Y, Paik HY, Joung H. Soybean and soy isoflavone intake indicate a positive change in bone mineral density for 2 years in young Korean women. Nutrition Research. 2008;28(1):25-30.

151. Sonoda T, Nagata Y, Mori M, Miyanaga N, Takashima N, Okumura K, et al. A case-control study of diet and prostate cancer in Japan: possible protective effect of traditional Japanese diet. Cancer Science. 2004;95(3):238-42.

152. Steffen LM, Kroenke CH, Yu X, Pereira MA, Slattery ML, Van Horn L, et al. Associations of plant food, dairy product, and meat intakes with 15-y incidence of elevated blood pressure in young black and white adults: the Coronary Artery Risk Development in Young Adults (CARDIA) Study2. The American Journal of Clinical Nutrition. 2005;82(6):1169-77.

153. Struijk EA, Fung TT, Rodríguez-Artalejo F, Bischoff-Ferrari HA, Hu FB, Willett WC, et al. Protein intake and risk of frailty among older women in the Nurses' Health Study. Journal of Cachexia, Sarcopenia and Muscle. 2022;13(3):1752-61.

154. Stuber JM, Vissers LET, Verschuren WMM, Boer JMA, van der Schouw YT, Sluijs I. Substitution among milk and yogurt products and the risk of incident type 2 diabetes in the EPIC-NL cohort. Journal of Human Nutrition and Dietetics. 2021;34(1):54-63.

155. Svensson T, Sawada N, Mimura M, Nozaki S, Shikimoto R, Tsugane S. Midlife Intakes of the Isoflavone Genistein and Soy and the Risk of Late-life Cognitive Impairment: The JPHC Saku Mental Health Study. Journal of Epidemiology. 2023;33(7):342-9.

156. Takata Y, Yang JJ, Yu D, Smith-Warner SA, Blot WJ, White E, et al. Calcium Intake and Lung Cancer Risk: A Pooled Analysis of 12 Prospective Cohort Studies. The Journal of Nutrition. 2023;153(7):2051-60.

157. Talaei M, Feng L, Yuan J-M, Pan A, Koh W-P. Dairy, soy, and calcium consumption and risk of cognitive impairment: the Singapore Chinese Health Study. European Journal of Nutrition. 2020;59(4):1541-52.

158. Talaei M, Koh W-P, van Dam RM, Yuan J-M, Pan A. Dietary Soy Intake Is Not Associated with Risk of Cardiovascular Disease Mortality in Singapore Chinese Adults. The Journal of Nutrition. 2014;144(6):921-8.

159. Tan M-M, Ho W-K, Yoon S-Y, Mariapun S, Hasan SN, Lee DS-C, et al. A case-control study of breast cancer risk factors in 7,663 women in Malaysia. PLOS ONE. 2018;13(9):e0203469.

160. Liyuan T, Zheng X, Tao H. Dietary diversity and all-cause mortality among Chinese adults aged 65 or older: A community-based cohort study. Asia Pacific Journal of Clinical Nutrition. 2020;29(1):152-60.

161. Teng GG, Pan A, Yuan J-M, Koh W-P. Food Sources of Protein and Risk of Incident Gout in the Singapore Chinese Health Study. Arthritis & Rheumatology. 2015;67(7):1933-42.

162. Thiébaut AC, Clavel-Chapelon F. [Fat consumption and breast cancer: preliminary results from the E3N-Epic cohort]. Bull Cancer. 2001;88(10):954-8.

163. Travis RC, Allen NE, Appleby PN, Spencer EA, Roddam AW, Key TJ. A prospective study of vegetarianism and isoflavone intake in relation to breast cancer risk in British women. International Journal of Cancer. 2008;122(3):705-10.

164. Vanegas JC, Afeiche MC, Gaskins AJ, Mínguez-Alarcón L, Williams PL, Wright DL, et al. Soy food intake and treatment outcomes of women undergoing assisted reproductive technology. Fertility and Sterility. 2015;103(3):749-55.e2.

165. Vitolins MZ, Case LD, Morgan TM, Miller MA, Burke GL. Soy use and vasomotor symptoms: Soy Estrogen Alternative follow-up study. Int J Womens Health. 2010;2:381-6.

166. Wada K, Tsuji M, Tamura T, Konishi K, Goto Y, Mizuta F, et al. Soy Isoflavone Intake and Bladder Cancer Risk in Japan: From the Takayama Study. Cancer Epidemiology, Biomarkers & Prevention. 2018;27(11):1371-5.

167. Wada K, Nakamura K, Tamai Y, Tsuji M, Kawachi T, Hori A, et al. Soy isoflavone intake and breast cancer risk in Japan: From the Takayama study. International Journal of Cancer. 2013;133(4):952-60.

168. Wada K, Tsuji M, Tamura T, Konishi K, Kawachi T, Hori A, et al. Soy isoflavone intake and stomach cancer risk in Japan: From the Takayama study. International Journal of Cancer. 2015;137(4):885-92.

169. Wang C, Yang Y, Xu W, Yu D, Wu J, Cai Q, et al. Legume Consumption and Gut Microbiome in Elderly Chinese Men and Women. The Journal of Nutrition. 2021;151(8):2399-408.

170. Wilunda C, Sawada N, Goto A, Yamaji T, Iwasaki M, Tsugane S, et al. Soy food and isoflavones are not associated with changes in serum lipids and glycohemoglobin concentrations among Japanese adults: a cohort study. European Journal of Nutrition. 2020;59(5):2075-87.

171. Woo HW, Hong S, Shin MH, Koh SB, Kim HC, Kim YM, et al. Sex-specific associations between dietary legume subtypes and type 2 diabetes in a prospective cohort study. Epidemiol Health. 2024;46:e2024083.

172. Woo HW, Kim MK, Lee Y-H, Shin DH, Shin M-H, Choi BY. Sex-specific associations of habitual intake of soy protein and isoflavones with risk of type 2 diabetes. Clinical Nutrition. 2021;40(1):127-36.

173. Woo HW, Kim MK, Lee Y-H, Shin DH, Shin M-H, Choi BY. Habitual consumption of soy protein and isoflavones and risk of metabolic syndrome in adults ≥ 40 years old: a prospective analysis of the Korean Multi-Rural Communities Cohort Study (MRCohort). European Journal of Nutrition. 2019;58(7):2835-50.

174. Wu AH, Koh WP, Wang R, Lee HP, Yu MC. Soy intake and breast cancer risk in Singapore Chinese Health Study. British Journal of Cancer. 2008;99(1):196-200.

175. Wu AHS, F. Z.; Seow, A.; Lee, H. P.; Yu, M. C. Soy intake and other lifestyle determinants of serum estrogen levels among postmenopausal chinese women in Singapore. CANCER EPIDEMIOLOGY BIOMARKERS & PREVENTION. 2002;11(9).

176. Wu H, Quan J, Wang X, Gu Y, Zhang S, Meng G, et al. Soy Food Consumption Is Inversely Associated with Handgrip Strength: Results from the TCLSIH Cohort Study. Nutrients [Internet]. 2023; 15(2).

177. Xiong J, Xu Y, Liu X, Wang X, Shan S, Crabbe MJC, et al. Prospective association of dietary soy and fibre intake with puberty timing: a cohort study among Chinese children. BMC Medicine. 2022;20(1):145.

178. Xue T, Wen J, Wan Q, Qin G, Yan L, Wang G, et al. Association of soy food with cardiovascular outcomes and all-cause mortality in a Chinese population: a nationwide prospective cohort study. European Journal of Nutrition. 2022;61(3):1609-20.

179. Yamasaki K, Kayaba K, Ishikawa S. Soy and Soy Products Intake, All-Cause Mortality, and Cause-Specific Mortality in Japan: The Jichi Medical School Cohort Study. Asia Pacific Journal of Public Health. 2014;27(5):531-41.

180. Yan F, Eshak ES, Shirai K, Dong J-Y, Muraki I, Tamakoshi A, et al. Soy Intake and Risk of Type 2 Diabetes Among Japanese Men and Women: JACC Study. Frontiers in Nutrition. 2022;Volume 8 - 2021.

181. Yang G, Shu XO, Chow W-H, Zhang X, Li H-L, Ji B-T, et al. Soy Food Intake and Risk of Lung Cancer: Evidence From the Shanghai Women's Health Study and a Meta-Analysis. American Journal of Epidemiology. 2012;176(10):846-55.

182. Yang G, Shu X-O, Jin F, Zhang X, Li H-L, Li Q, et al. Longitudinal study of soy food intake and blood pressure among middle-aged and elderly Chinese women2. The American Journal of Clinical Nutrition. 2005;81(5):1012-7.

183. Yang G, Shu X-O, Li H, Chow W-H, Cai H, Zhang X, et al. Prospective cohort study of soy food intake and colorectal cancer risk in women2. The American Journal of Clinical Nutrition. 2009;89(2):577-83.

184. Yu D, Zhang X, Xiang Y-B, Yang G, Li H, Fazio S, et al. Association of soy food intake with risk and biomarkers of coronary heart disease in Chinese men. International Journal of Cardiology. 2014;172(2):e285-e7.

185. Zhang S, Kumari S, Gu Y, Wu X, Li X, Meng G, et al. Soy Food Intake Is Inversely Associated with Newly Diagnosed Nonalcoholic Fatty Liver Disease in the TCLSIH Cohort Study. The Journal of Nutrition. 2020;150(12):3280-7.

186. Zhang T, Jiang G, Li F, Gu X, Zhai Y, Xu L, et al. Soy product consumption and the risk of major depressive disorder in older adults: Evidence from a cohort study. Frontiers in Psychiatry. 2022;Volume 13 - 2022.

187. Zhang X, Shu XO, Yang G, Zheng W, Gao Y-T, Li Q, et al. Soy Food Consumption Is Associated with Lower Risk of Coronary Heart Disease in Chinese Women. The Journal of Nutrition. 2003;133(9):2874-8.

188. Zhang X, Shu X-O, Li H, Yang G, Li Q, Gao Y-T, et al. Prospective Cohort Study of Soy Food Consumption and Risk of Bone Fracture Among Postmenopausal Women. Archives of Internal Medicine. 2005;165(16):1890-5.

189. Zhuanping ZR, Liao; Qing, Chen; Sidong, Chen. The Association between <ovid:i>SIRT1</ovid:i> Genetic Variation and Type 2 Diabetes Mellitus Is Influenced by Dietary Intake in Elderly Chinese. Iran J Public Health. 2018;47(9):1272-80.

190. Z. Movassagh E, Kontulainen S, Baxter-Jones ADG, Whiting S, Szafron M, Papadimitropoulos M, et al. Are milk and alternatives and fruit and vegetable intakes during adolescence associated with cortical and trabecular bone structure, density, and strength in adulthood? Osteoporosis International. 2017;28(2):609-19.

191. Nishio K, Niwa Y, Toyoshima H, Tamakoshi K, Kondo T, Yatsuya H, et al. Consumption of soy foods and the risk of breast cancer: findings from the Japan Collaborative Cohort (JACC) Study. Cancer Causes Control. 2007;18(8):801-8.

192. Abe SK, Sawada N, Ishihara J, Takachi R, Mori N, Yamaji T, et al. Comparison between the impact of fermented and unfermented soy intake on the risk of liver cancer: the JPHC Study. European Journal of Nutrition. 2021;60(3):1389-401.

193. Cao Y, Taylor AW, Zhen S, Adams R, Appleton S, Shi Z. Soy Isoflavone Intake and Sleep Parameters over 5 Years among Chinese Adults: Longitudinal Analysis from the Jiangsu Nutrition Study. Journal of the Academy of Nutrition and Dietetics. 2017;117(4):536-44.e2.

194. Chen Z, Qian F, Hu Y, Voortman T, Li Y, Rimm EB, et al. Dietary phytoestrogens and total and cause-specific mortality: results from 2 prospective cohort studies. The American Journal of Clinical Nutrition. 2023;117(1):130-40.

195. Deng Z, Xie D, Cai J, Jiang J, Pan D, Liao H, et al. Different types of milk consumption and the risk of dementia: Analysis from a large-scale cohort study. Clinical Nutrition. 2023;42(10):2058-67.

196. Ding M, Pan A, Manson JE, Willett WC, Malik V, Rosner B, et al. Consumption of soy foods and isoflavones and risk of type 2 diabetes: a pooled analysis of three US cohorts. European Journal of Clinical Nutrition. 2016;70(12):1381-7.

197. Dunneram Y, Chung H-F, Cade JE, Greenwood DC, Dobson AJ, Mitchell ES, et al. Soy intake and vasomotor menopausal symptoms among midlife women: a pooled analysis of five studies from the InterLACE consortium. European Journal of Clinical Nutrition. 2019;73(11):1501-11.

198. Beavers KM, Serra MC, Beavers DP, Cooke MB, Willoughby DS. Soy and the exercise-induced inflammatory response in postmenopausal women. Applied Physiology, Nutrition, and Metabolism. 2010;35(3):261-9.

199. Chiang F-YI, Chen J-R, Lee W-J, Yang S-C. Effects of Milk or Soy Milk Combined with Mild Resistance Exercise on the Muscle Mass and Muscle Strength in Very Old Nursing Home Residents with Sarcopenia. Foods [Internet]. 2021; 10(11).

200. Dettmer M, Alekel DL, Lasrado JA, Messina M, Carriquiry A, Heiberger K, et al. The Effect of Soy Protein Beverages on Serum Cell Adhesion Molecule Concentrations in Prehypertensive/Stage 1 Hypertensive Individuals. Journal of the American College of Nutrition. 2012;31(2):100-10.

201. Faghih S, Hedayati M, Abadi A, Kimiagar SM. Comparison of the effects of cows milk, fortified soy milk, and calcium supplement on plasma adipocytokines in overweight or obese women. 2010.

202. Fernandez-Raudales D, Hoeflinger JL, Bringe NA, Cox SB, Dowd SE, Miller MJ, et al. Consumption of different soymilk formulations differentially affects the gut microbiomes of overweight and obese men. Gut Microbes. 2012;3(6):490-500.

203. Fournier LR, Ryan Borchers TA, Robison LM, Wiediger M, Park JS, Chew BP, et al. The effects of soy milk and isoflavone supplements on cognitive performance in healthy, postmenopausal women. J Nutr Health Aging. 2007;11(2):155-64.

204. Nourozi M, Haghollahi F, Ramezanzadeh F, Hanachi P. Effect of Soy Milk Consumption on Quality of Life in Iranian Postmenopausal Women. J Family Reprod Health. 2015;9(2):93-100.

205. Ryan-Borchers TA, Park JS, Chew BP, McGuire MK, Fournier LR, Beerman KA. Soy isoflavones modulate immune function in healthy postmenopausal women2. The American Journal of Clinical Nutrition. 2006;83(5):1118-25.

206. Ryan-Borchers T, Chew B, Park JS, McGuire M, Fournier L, Beerman K. Effects of Dietary and Supplemental Forms of Isoflavones on Thyroid Function in Healthy Postmenopausal Women. Topics in Clinical Nutrition. 2008;23(1).

207. Serra MC, Beavers KM, Beavers DP, Willoughby DS. Effects of 28 days of dairy or soy ingestion on skeletal markers of inflammation and proteolysis in post-menopausal women. Nutrition and Health. 2012;21(2):117-30.

208. NCT05191160. The Soy Treatment Evaluation for Metabolic Health (STEM) Trial. 2021.

209. Rosendahl-Riise H. The Difference in Health Outcomes After Drinking Cow's Milk Compared to Oat Milk - a Pilot Randomized Controlled Study. University of Bergen. 2025. Available online: <https://clinicaltrials.gov/study/NCT06764173> (last accessed: 31.10.2025).

210. Campbell B. Clinical Trial to Study the Effects of Dietary Flax Beverage on Memory and Cognition. St. Boniface Hospital. 2025. Available online: <https://clinicaltrials.gov/study/NCT04645927>. (Last accessed: 31.10.2025).

211. ChiCTR-IPR-16008140. Randomized controlled trial of lactose intolerance patients treated with milk lactose hydrolysis, soymilk on bone health in postmenopausal women. 2016.

212. IUNS 22nd International Congress of Nutrition – Abstracts. Annals of Nutrition and Metabolism. 2023;79(Suppl. 1):14-1172.

213. JPRN-UMIN000027032. Effects of long-term ingestion of protein-rich milk on muscle strength in university students. 2017.

214. Abstracts of Oral Communications. Climacteric. 2014;17(sup1):22-43.

215. NCT06173271. The Effect of a Multi-intervention Program and Protein Food Sources on Preventing and Mitigating Sarcopenia. 2023.

216. Shin M-H, Hwang S-M, Bae J-M, Lee M-S, Kim D-H, Li Z-M, et al. Abstract 4807: Soy and salty food intake and risk of stomach cancer in Seoul Male Cohort. Cancer Research. 2013;73(8_Supplement):4807-.

217. 15TH INTERNATIONAL THYROID CONGRESS PROGRAM AND MEETING ABSTRACTS. Thyroid®. 2015;25(S1):P-1-A-337.

218. TCTR20230425003. Effectiveness and safety of hemp seed drink in stress or anxiety patients: a randomized double-blinded controlled trial. 2023.

219. JPRN-UMIN000041850. Effects of nutritional condition, body composition and physical ability by continuous ingestion of soy-protein drink in athletes. 2020.

220. Chilibeck P. Milk Versus a Pea-based Beverage for Bone and Muscle Health in Young Athletes. University of Saskatchewan. 2022. Available online: <https://ctv.veeva.com/study/milk-versus-a-pea-based-beverage-for-bone-and-muscle-health-in-young-athletes> (last accessed: 31.10.2025).

221. Beavers KM, Serra MC, Beavers DP, Hudson GM, Willoughby DS. The lipid-lowering effects of 4 weeks of daily soymilk or dairy milk ingestion in a postmenopausal female population. J Med Food. 2010;13(3):650-6.

222. Faghih S, Abadi AR, Hedayati M, Kimiagar SM. Comparison of the effects of cows' milk, fortified soy milk, and calcium supplement on weight and fat loss in premenopausal overweight and obese women. Nutr Metab Cardiovasc Dis. 2011;21(7):499-503.

223. Gui JC, Brašić JR, Liu XD, Gong GY, Zhang GM, Liu CJ, et al. Bone mineral density in postmenopausal Chinese women treated with calcium fortification in soymilk and cow's milk. Osteoporos Int. 2012;23(5):1563-70.

224. Mitchell JH, Collins AR. Effects of a soy milk supplement on plasma cholesterol levels and oxidative DNA damage in men--a pilot study. Eur J Nutr. 1999;38(3):143-8.

225. Onning G, Akesson B, Oste R, Lundquist I. Effects of consumption of oat milk, soya milk, or cow's milk on plasma lipids and antioxidative capacity in healthy subjects. Ann Nutr Metab. 1998;42(4):211-20.

226. Rivas M, Garay RP, Escanero JF, Cia P, Jr., Cia P, Alda JO. Soy milk lowers blood pressure in men and women with mild to moderate essential hypertension. J Nutr. 2002;132(7):1900-2.

227. Azadbakht L, Nurbakhsh S. Effect of soy drink replacement in a weight reducing diet on anthropometric values and blood pressure among overweight and obese female youths. Asia Pac J Clin Nutr. 2011;20(3):383-9.

228. Bricarello LP, Kasinski N, Bertolami MC, Faludi A, Pinto LA, Relvas WG, et al. Comparison between the effects of soy milk and non-fat cow milk on lipid profile and lipid peroxidation in patients with primary hypercholesterolemia. Nutrition. 2004;20(2):200-4.

229. Gardner CD, Messina M, Kiazand A, Morris JL, Franke AA. Effect of two types of soy milk and dairy milk on plasma lipids in hypercholesterolemic adults: a randomized trial. J Am Coll Nutr. 2007;26(6):669-77.

230. Keshavarz SA, Nourieh Z, Attar MJ, Azadbakht L. Effect of Soymilk Consumption on Waist Circumference and Cardiovascular Risks among Overweight and Obese Female Adults. Int J Prev Med. 2012;3(11):798-805.

231. Onning G, Wallmark A, Persson M, Akesson B, Elmståhl S, Oste R. Consumption of oat milk for 5 weeks lowers serum cholesterol and LDL cholesterol in free-living men with moderate hypercholesterolemia. Ann Nutr Metab. 1999;43(5):301-9.

232. Sirtori CR, Bosisio R, Pazzucconi F, Bondioli A, Gatti E, Lovati MR, et al. Soy milk with a high glycitein content does not reduce low-density lipoprotein cholesterolemia in type II hypercholesterolemic patients. Ann Nutr Metab. 2002;46(2):88-92.

233. Sirtori CR, Pazzucconi F, Colombo L, Battistin P, Bondioli A, Descheemaeker K. Double-blind study of the addition of high-protein soya milk v. cows' milk to the diet of patients with severe hypercholesterolaemia and resistance to or intolerance of statins. Br J Nutr. 1999;82(2):91-6.

234. Steele M. The effect on serum cholesterol levels of substituting milk with a soya beverage. 1992.

235. Brignardello-Petersen R, Bonner A, Alexander PE, Siemieniuk RA, Furukawa TA, Rochwerg B, et al. Advances in the GRADE approach to rate the certainty in estimates from a network meta-analysis. J Clin Epidemiol. 2018;93:36-44.

236. Izcovich A, Chu DK, Mustafa RA, Guyatt G, Brignardello-Petersen R. A guide and pragmatic considerations for applying GRADE to network meta-analysis. BMJ. 2023;381:e074495.

237. Erlich MN, Ghidanac D, Blanco Mejia S, Khan TA, Chiavaroli L, Zurbau A, et al. A systematic review and meta-analysis of randomized trials of substituting soymilk for cow’s milk and intermediate cardiometabolic outcomes: understanding the impact of dairy alternatives in the transition to plant-based diets on cardiometabolic health. BMC Med. 2024;22(1):336.

238. Langsted A, Freiberg JJ, Nordestgaard BG. Fasting and nonfasting lipid levels: influence of normal food intake on lipids, lipoproteins, apolipoproteins, and cardiovascular risk prediction. Circulation. 2008;118(20):2047-56.

239. Zhou S, Cheng F, He J, Xu T, Zhang X, Wan S, et al. Effects of high-quality protein supplementation on cardiovascular risk factors in individuals with metabolic diseases: A systematic review and meta-analysis of randomized controlled trials. Clin Nutr. 2024;43(8):1740-50.

240. Wei M, Gibbons LW, Mitchell TL, Kampert JB, Stern MP, Blair SN. Low fasting plasma glucose level as a predictor of cardiovascular disease and all-cause mortality. Circulation. 2000;101(17):2047-52.
